# Prior infections are associated with smaller hippocampal volume in older women

**DOI:** 10.3389/frdem.2024.1297193

**Published:** 2024-02-07

**Authors:** Vladimir A. Popov, Svetlana Ukraintseva, Hongzhe Duan, Konstantin G. Arbeev, Anatoliy I. Yashin

**Affiliations:** Biodemography of Aging Research Unit, Social Science Research Institute, Duke University, Durham, NC, United States

**Keywords:** hippocampus, infection, verbal, visual-spatial, memory, aging, Alzheimer's disease, dementia

## Abstract

Accumulating evidence suggests that infections may play a major role in Alzheimer's disease (AD), however, mechanism is unclear, as multiple pathways may be involved. One possibility is that infections could contribute to neurodegeneration directly by promoting neuronal death. We explored relationships between history of infections and brain hippocampal volume (HV), a major biomarker of neurodegeneration, in a subsample of the UK Biobank (UKB) participants. Infectious disease diagnoses were based on ICD10 codes. The left/right HV was measured by the magnetic resonance imaging (MRI) in cubic millimeters and normalized. Analysis of variance (ANOVA), Welch test, and regression were used to examine statistical significance. We found that HV was significantly lower in women aged 60–75, as well as 65–80, years, with history of infections, compared to same age women without such history. The effect size increased with age faster for the left vs. right HV. Results for males didn't reach statistical significance. Results of our study support a major role of adult infections in neurodegeneration in women. The detrimental effect of infections on HV became stronger with age, in line with declining resilience and increasing brain vulnerability to stressors due to aging. The faster increase in the effect size observed for the left vs. right HV may indicate that female verbal memory degrades faster over time than visual-spatial memory. The observed sex difference may reflect a higher vulnerability of female brain to infection-related factors, which in turn may contribute to a higher risk of AD in women compared to men.

## 1 Introduction

Alzheimer's disease (AD) is currently incurable, progressive degenerative disease which first affects the part of the brain associated with learning and memory. AD is the most common form of dementia comprising about 60–70% of the about 55 million of its worldwide cases. This number is expected to rise to 78 million by 2030 and 139 million by 2050 (World Alzheimer Report and Alzheimers Disease International, 2022; World Health Organization, 2022). An estimated 6.2 million Americans age of 65 and older are living with AD today. This number could grow to 13.8 million by 2060 making the development of efficient medications and preventive measures against AD a high priority problem. The solution of this problem could be substantially facilitated if the mechanisms involved in the AD regulation were better understood (2022 Alzheimer's disease facts figures, 2022).

The hippocampus is a brain region critical for learning and memory, and it is one of the most affected areas in AD (van der Flier and Scheltens, 2009; Rao et al., 2022). The hippocampal volume (HV) naturally declines with aging (Harman, 2001; López-Otín et al., 2013), and is adversely affected by a range of conditions. Large hippocampal size is closely linked with good memory and cognitive function; conversely, atrophy of the hippocampus is associated with the development of dementia (Fotuhi et al., 2012). In patients with mild cognitive impairment (MCI), a high rate of decline in hippocampal size strongly heralds conversion to AD. Accelerated progression of atrophy is also associated with rapid cognitive decline in both MCI and AD. Significant positive correlations between bilateral hippocampal volumes and both verbal [Hopkins Verbal Learning Test-Revised (Benedict et al., 1998)] and non-verbal [Brief Visuospatial Memory Test-Revised (Benedict et al., 1996)] memory measures were found (Bonner-Jackson et al., 2015). Study (Ezzati et al., 2016) provides further evidence of divergent functional specialization of the right and left hippocampus. The right hippocampus plays a critical role in spatial memory in older adults, while the role of verbal memory is more prominent in the left hippocampus (Ezzati et al., 2016). Overall, reduced HV (as measured by the magnetic resonance imaging, MRI) is considered a major biomarker of neurodegeneration at early stages of AD (van der Flier and Scheltens, 2009; Rao et al., 2022).

Accumulating evidence suggests that infections may play a major role in AD, however, exact mechanism is unclear, as multiple pathways may be involved (Fülöp et al., 2020; Vigasova et al., 2021). One possibility is that infections might contribute to neurodegeneration directly by promoting neuronal death. If true, then the negative impact of exposure to infectious diseases on HV can be expected. In this study, we explore associations between the relatively recent history of adult infections and the brain hippocampal volume in a subsample of the UK Biobank older participants.

## 2 Material and methods

### 2.1 Data and phenotypes

This study was performed using a sample of the UK Biobank (UKB) data (UK Biobank, 2023). The UKB is a population-based study with extensive genetic and phenotypic data for approximately 500,000 individuals from across the UK. Data for the study were obtained (October 2022) from the UKB database (UK Biobank, 2023). Written informed consent was obtained by the UKB from the participants in accordance with the UK national legislation and the UKB requirements. The latest (at the time of calculations) available information on participants' withdrawal in UKB was taken into account. All analyses were performed on a subset of the database with individuals recruited starting from 2006 and those having data regarding infectious and parasitic disease. Below, the term “infectious” will be used instead of “infectious/parasitic” for conciseness. The terms “infectious disease” and “infection” were considered interchangeable.

The infectious diseases with the following International Classification of Diseases 10th Revision (ICD10) codes occurring during the period from January 1, 2009 to October 15, 2014 (selected to ensure that infectious diseases occurred before MRI visit) were used for the analysis (International Statistical Classification of Diseases Related Health Problems 10th Revision, 2019; UK Biobank, 2023): Chapter I: certain infectious and parasitic diseases (A00-B99); Chapter IX: acute pericarditis (I30), acute/subacute endocarditis (I33), acute myocarditis (I40); Chapter X: influenza and pneumonia (J09–J18); Chapter X: other acute lower respiratory infections (J20–J22); Chapter XI: acute appendicitis (K35), acute pancreatitis (K85); Chapter XII: acute lymphadenitis (L04).

Among the subjects that had information on these ICD10 codes, participants aged between 60 and 80 years at time of the neuroimaging visit (between January 15, 2015 and October 31, 2019) were selected. We created two partially overlapping age groups (60–75 and 65–80 years), with younger and older mean age and age range, to see how the HV changes with increasing age of the group. Note that the aim of this study was to investigate the relationship between recent history of infections and HV. So, the temporal interval for infectious events and MRI visit was chosen in such a way that gave, on average, 5 years duration between the time when infectious disease was diagnosed and the time when HV was measured. Thus, in this study, the 5 years duration specified the meaning of “recent” history of adult infections (or simply, for brevity: history of infections).

The image-derived left and right HV was measured in cubic millimeters (mm^3^), and respective information was obtained from the UKB data-fields 25,019 and 25,020, respectively. To normalize for head size, these measurements were multiplied by the head size scaling factor obtained from the UKB data-field 25,000 (Smith et al., 2022; Supplementary material, MRI measurements). Individuals, included in the analysis and stratified by women and men (Table 1), were divided in two groups according to their history of infections before the neuroimaging visit: *Infs*: those who had one or more ICD10 codes for infectious diseases, and *noInfs*: those who had not any such code.

**Table 1:** Characteristics of the UK Biobank sample used in this study.

**Group/subjects**	**Females, age 60–75**	**Males, age 60–75**	**Females, age 65–80**	**Males, age 65–80**
History of infections	407	330	321	277
No history of infections	5,285	4,974	4,009	4,462
All	5,692	5,304	4,330	4,739

The list of covariates used in this study includes potential confounders that may be relevant to both brain volume and vulnerability to infections, such as age, smoking, drinking, education status, bodyweight, body mass index (BMI), depression, stroke, hypertension, diabetes, cancer (all sites), sleep disorders, hematocrit, *T*-cell count, and exposure to air pollution represented by particulate matter with particle diameter ≤ 2.5 micrometers (PM2.5) or ≤10 micrometer (PM10), nitric oxide (NO), and nitrogen dioxide (NO2) (Fotuhi et al., 2012; de Brouwer et al., 2018). We also considered rs429853 (C), a proxy single nucleotide polymorphism (SNP) for *APOE4*, as well as SNPs rs6859 (A) and rs2075650 (G) in *NECTIN2* and *TOMM40* genes, respectively, which are major genetic risk factors for AD that may also be involved in brain vulnerability to infections (Yashin et al., 2018; Akushevich et al., 2023; Ukraintseva et al., 2023; Yuan et al., 2023). Additional details about these covariates are provided in Supplementary Tables 1, 2.

### 2.2 Analytic approach

Analysis of variance (ANOVA), Tukey's test, and the Welch test (Welch, 1947; Chambers et al., 1992; Yandell, 1997) were utilized. We considered a set consisting of all basic regression models having HV as a response variable and independent variables for age (*Age)* and history of infections (*infs*) with linear terms and their pairwise interactions:

HV = *Intercept* + *b*_1_*Age + *b*_2_*infs + *b*_12_*Age*infs
HV = *Intercept* + *b*_1_*Age + *b*_12_*Age*infs
HV = *Intercept* + *b*_2_*infs + *b*_12_*Age*infs
HV = *Intercept* + *b*_12_*Age*infs
HV = *Intercept* + *b*_1_*Age +*b*_2_* *infs*
HV = *Intercept* + *b*_1_*Age
HV = *Intercept* + *b*_2_*infs
HV = *Intercept*,

where *Intercept* is a constant called the bias term (or intercept term), *b*_1_, *b*_2_, *b*_12_ are the regression coefficients corresponding to the Age, infs, Age^*^infs terms in the regression model. The regression models were evaluated using the Akaike information criterion (AIC) (Akaike, 1973). The optimal, with respect to the minimal AIC criteria, significant regression model was found for the regression set described above. R standard software packages (version 3.6.3), along with *glmulti* package (Calcagno, 2022), were utilized.

We also compared proportions (%) of covariates relevant to AD, and (where applicable) mean values of covariates, among individuals with history of infection (*Infs*) and without such history (*noInfs*). The list of covariates is provided in *Data and phenotypes* subsection of Material and Methods, and in Supplementary Tables 1, 2.

## 3 Results

### 3.1 Groups comparison

**Figure 1:**
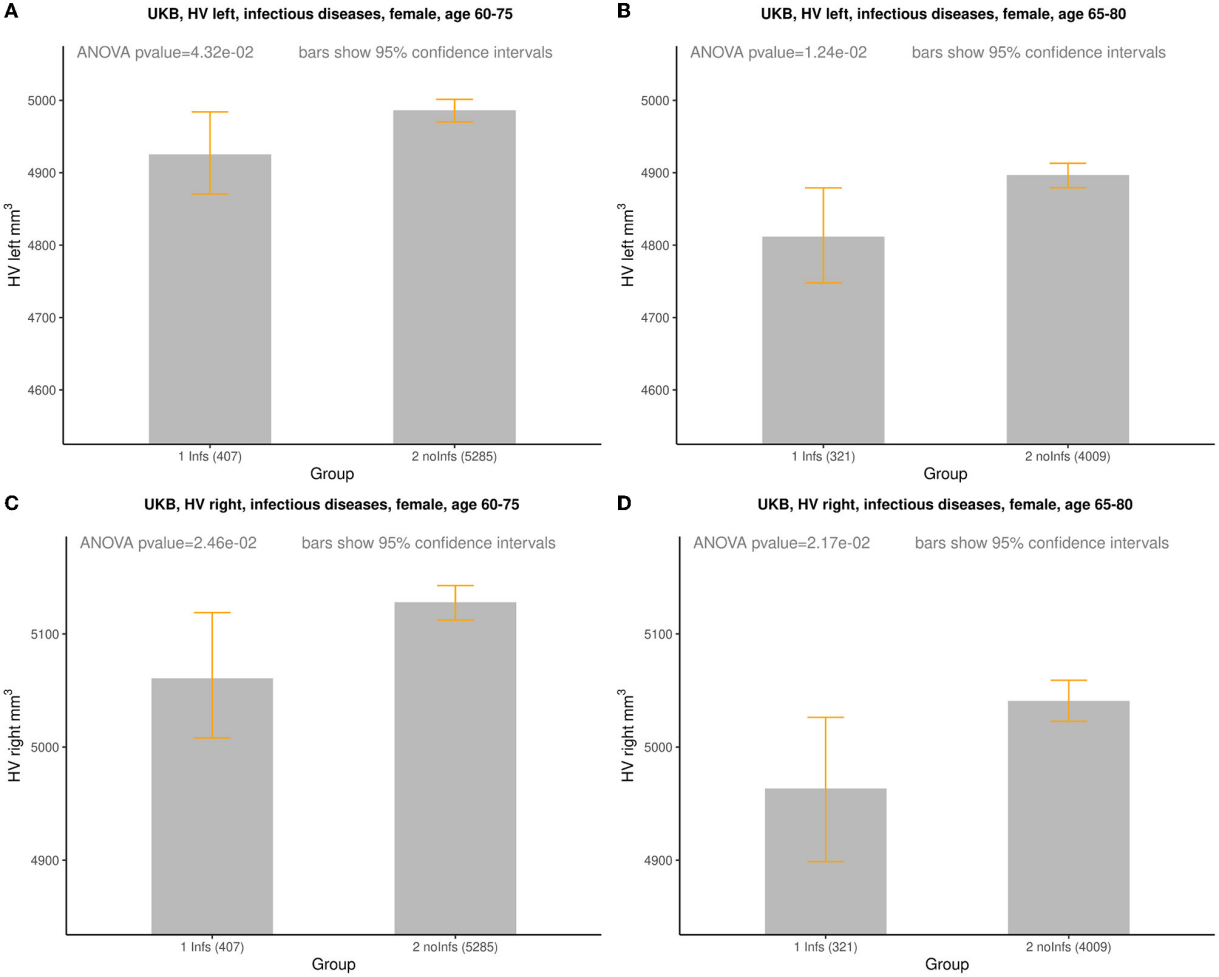
Comparison of HV (mm^3^) between females with (*Infs*) and without (*noInfs*) history of infectious diseases. Age is age at time of MRI scan. Descriptive statistics for respective groups are shown in subplots: **(A)** UKB, left HV, females, age 60–75. *Infs* [HV: min = 2,460, max = 6,832, mean (*m*) = 4,925, standard deviation (sd) = 588]; *noInfs* (HV: min = 1,709, max = 10,121, *m* = 4,986, sd = 584). **(B)** UKB, left HV, females, age 65–80. *Infs* (HV: min = 2,460, max = 6,832, *m* = 4,812, sd = 591); *noInfs* (HV: min = 1,709, max = 9,645, *m* = 4,897, sd = 587). **(C)** UKB, right HV, females, age 60–75. *Infs* (HV: min = 2,119, max = 7,287, *m* = 5,061, sd = 558); *noInfs* (HV: min = 1,947, max = 10,063, *m* = 5,128, sd = 583). **(D)** UKB right, females, age 65–80. *Infs* (HV: min = 2,119, max = 7,287, *m* = 4,963, sd = 566); *noInfs* (HV: min = 1,947, max = 8,785, *m* = 5,041, sd = 583).

In order to more clearly represent the raw data, it was divided into age groups with subgroups with and without history of infection. One can notice that, on average, the HV values for females with the history of infection tend to be smaller than for those without of history of infection (Supplementary Figure 1; Supplementary Tables 3–6) while for males with the history of infection the HV values lie higher and lower for those without of history of infection (Supplementary Figure 2; Supplementary Tables 7–10). This visual observation might indicate that there was a difference between Infs and noInfs groups. The results of rigorous comparison between groups of women with and without history of infections are presented in Figure 1, in Table 2, and in Supplementary Figure 3. The results confirmed our visual preliminary observation. We found that the left/right HV was significantly (*P*-value < 0.05) smaller in women with the history of infections, compared to women without such history, for both age intervals (60–75 and 65–80 years). The effect sizes for left HV for women aged 60–75 and 65–80 were −61 and −85 mm^3^, respectively. The effect sizes for the right HV for women aged 60–75 and 65–80 were −67 and −77 mm^3^, respectively (see Table 2). No statistically significant difference in the left/right HV was found between males with and without history of infections, aged 60–75 or 65–80 years.

**Table 2:** Comparison of HV between women with, and without, history of infections.

**Test**	***P*-value**	**95% confidence intervals**	**HV estimate (mm^3^)**
**Females, age 60–75, HV (mm** ^3^ **) left**			
ANOVA	4.32e-02		
HV, *Infs*		(4,867, 4,983)	4,925
HV, *noInfs*		(4,970, 5,001)	4,986
Effect size		(−120, −2)	−61
**Females, age 65–80, HV (mm** ^3^ **) left**			
ANOVA	1.24e-02		
HV, *Infs*		(4,748, 4,879)	4,812
HV, *noInfs*		(4,879, 4,913)	4,897
Effect size		(−152, −18)	−85
**Females, age 60–75, HV (mm** ^3^ **) right**			
ANOVA	2.46e-02		
HV, *Infs*		(5,006, 5,117)	5,061
HV, *noInfs*		(5,112, 5,143)	5,128
Effect size		(−126, −9)	−67
**Females, age 65–80, HV (mm** ^3^ **) right**			
ANOVA	2.17e-02		
HV, *Infs*		(4,898, 5,026)	4,963
HV, *noInfs*		(5,023, 5,059)	5,041
Effect size		(−144, −11)	−77
**Males, age 60–75, HV (mm** ^3^ **) left**			
ANOVA	3.26e-01		
HV, *Infs*		(4,565, 4,693)	4,630
HV, *noInfs*		(4,647, 4,680)	4,664
Effect size		(−101, 34)	−34
**Males, age 65–80, HV (mm** ^3^ **) left**			
ANOVA	8.73e-01		
HV, *Infs*		(4,473, 4,613)	4,540
HV, *noInfs*		(4,528, 4,564)	4,546
Effect size		(−67, 79)	−6
**Males, age 60–75, HV (mm** ^3^ **) right**			
ANOVA	6.74e-01		
HV, *Infs*		(4,767, 4,896)	4,837
HV, *noInfs*		(4,804, 4,839)	4,822
Effect size		(−55, 84)	15
**Males, age 65–80, HV (mm** ^3^ **) right**			
ANOVA	5.08e-01		
HV, *Infs*		(4,658, 4,807)	4,728
HV, *noInfs*		(4,683, 4,721)	4,703
Effect size		(−103, 51)	−26

The effect size equals to the difference between the left/right HV mean value in group Infs and group noInfs.

The decrease in HV became more pronounced with age in women aged 60–75 years: from 1.1% at age 60–1.6% at age 75, and from 1.0% at age 60–1.4% at age 75, for the left HV and the right HV, respectively. Similar dynamics was observed for women aged 65–80 years. The decrease in HV became more pronounced with age in this group too: from 1.3% at age 65–1.7% at age 80 and from 1.1% at age 60–1.5% at age 75, for the left HV and the right HV, respectively.

Since adult HV, on average, declines with age (Harman, 2001; Fotuhi et al., 2012; López-Otín et al., 2013), and is naturally larger in younger than in older individuals, we had to ensure that the observed detrimental effect of the history of infections on HV was not due to potentially younger mean age of *noInfs* vs. *Infs* group. We, therefore, additionally compared the age distributions between Infs and noInfs groups (Supplementary Tables 12, 13), and found that the difference between mean ages in Infs and noInfs groups was not significant for males and females aged 60–75 and 65–80 years. Thus, the detrimental effect of the history of infections on HV (averaged by age) was not due to age differences between the *Infs* and *noInfs* groups.

### 3.2 Regression analysis

Results of the regression analysis (Table 3) showed that the left and the right HV decreased with age in women (men) aged 60–75 years, losing about 29 (32) mm^3^/year, and 28 (31) mm^3^/year, respectively. For women (men) aged 65–80 years, the left HV and the right HV also diminished with age, losing about 34 (41) mm^3^/year, and 32 (38) mm^3^/year, respectively. The decrease in HV accelerated with age. The HV values were assessed using basic linear regression set including pairwise interactions of independent variables *Age, infs* (eight models described in Section 2). The optimal, with min (AIC) criteria, significant model was determined per each group of female participants age 60–75 and 65–80 and left/right HV values (Table 3). Results for males did not reach a statistical significance.

**Table 3:** Regression analysis, females, age 60–75 and age 65–80.

**Model/term**	**Estimate**	**Std. error**	***P*-value**
**Best model for HV (mm** ^3^ **) left, female 60–75**			
Intercept	6,909.22 (mm^3^)	133.37	< 1.00e-50
*Age*	−28.75 (mm^3^/year)	2.00	6.75e-46
*Age* ^*^ *infs*	−0.99 (mm^3^/year)	0.45	3.39e-02
**Best model for HV (mm** ^3^ **) left, female 65–80**			
Intercept	7,278.30 (mm^3^)	184.81	< 1.00e-50
*Age*	−33.91 (mm^3^/year)	2.64	5.66e-37
*infs*	−86.42 (mm^3^)	35.13	1.39e-02
**Best model for HV (mm** ^3^ **) right, female 60–75**			
Intercept	7,018.88 (mm^3^)	132.29	< 1.00e-50
*Age*	−28.31 (mm^3^/year)	1.98	2.98e-45
Age^*^i*nfs*	−0.91 (mm^3^/year)	0.46	4.99e-02
**Best model for HV (mm** ^3^ **) right, female 65–80**			
Intercept	7,274.61 (mm^3^)	185.67	< 1.00e-50
*Age*	−31.88 (mm^3^/year)	2.65	1.11e-32
*infs*	−69.69 (mm^3^)	35.29	4.84e-02
**Best model for HV (mm** ^3^ **) left, male 60–75**			
Intercept	6,814.96 (mm^3^)	149.78	< 1.00e-50
*Age*	−31.84 (mm^3^/year)	2.22	1.45e-45
**Best model for HV (mm** ^3^ **) left, male 65–80**			
Intercept	7,426.24 (mm^3^)	189.40	< 1.00e-50
*Age*	−40.68 (mm^3^/year)	2.68	1.79e-50
**Best model for HV (mm** ^3^ **) right, male 60–75**			
Intercept	6,934.84 (mm^3^)	152.59	< 1.00e-50
*Age*	−31.26 (mm^3^/year)	2.26	1.60e-42
**Best model for HV (mm** ^3^ **) right, male 65–80**			
Intercept	7,426.48 (mm^3^)	194.34	< 1.00e-50
*Age*	−38.41 (mm^3^/year)	2.75	3.44e-43

Here, significance means that all regression coefficient were significant (*P*-value < 0.05) in a specific model, non-significance means the opposite. The numbers in the Estimate column correspond to the coefficients for the regression terms shown in the Model/Term column. As an example, for HV = HV left and females aged 60–75, all regression models are presented in ascending order by AIC value in the Supplementary Table 11.

Response variable HV = HV (mm^3^) left and HV = HV (mm^3^) right, independent variables: *infs*, 1 (with history of infections); *infs*, 0 (without history of infections); *Age*, age at the time attending assessment center during imaging visit. Per four regression sets, each corresponding to one age groups (60–75 or age 65–80) and HV values (left or right HV), all 8 models (with linear terms and their pairwise interactions) in each regression set were analyzed and the best model (significant and with minimal AIC value) is presented in this table.

To ensure that the observed effects for female HV were not due to differences in proportions of diseases and other covariates of a potential relevance to AD between the *Infs* and *noInfs* groups, we estimated respective proportions. The full list of covariates is provided in Section 2.1, and in Supplementary Tables 1, 2. We did not find significant differences in proportions (%) of covariates relevant to AD, or in mean values of covariates, between women aged 60–75 years with history of infection (*Infs*) and without such history (*noInfs*) (Supplementary Table 1). The proportion of smokers was slightly higher among women aged 65–80 years with history of infection than without such history (*p*-value = 0.04) (Supplementary Table 2). Since smoking may potentially affect HV (Linli et al., 2023), we extended regression analysis by adding the variable about smoking status: *smoker* = 1 (if the subject was ever smoker), *smoker* = 0 (if the subject was a non-smoker), and analyzed all linear regression models listed above with response variable left HV and right HV, and independent variables *Age, infs, smoker* (including their pairwise interactions). This analysis showed that for the left and right HV, best models with three independent variables *Age, infs, smoker* (with minimal AIC value) were the same (Supplementary Tables 14, 15), as it was found using only two independent variables *Age, infs*, without taking into account smoking status of females aged 65–80 years (Table 3; Supplementary Table 16).

Lastly, let us make a note regarding stronger association of infections with HV in older females. Based on Table 3, consider the term K^*^Age^*^infs for the “Best model for HV left, female age 60–75”, where *K* = −0.99 mm^3^/year. Here, independent variable *Age* is age at the time attending assessment center during imaging visit, independent variable *infs* means: *infs* = 1 (with history of infections), *infs* = 0 (without history of infections). Let us make a rough estimate of the mean of the term K^*^Age^*^infs for females in the age interval 60–75 with *infs* = 1: mean (K^*^Age^*^infs) = (K^*^60^*^1 + K^*^75^*^1)/2 = (−0.99^*^60 + −0.99^*^75)/2 = −66.825 (mm^3^). The obtained value mean (K^*^Age^*^infs) may be interpreted as an average decrease in the left HV due to the variable *infs* only (with variable *infs* going from 0 to 1). Compare this decrease −66.825 mm^3^ with the coefficient for the term *infs* for the “Best model for HV (mm^3^) left, female 65–80”: −86.42 mm^3^. This value is interpreted as an average decrease in the left HV due to the variable *infs*. Value −86.42 mm^3^ is <-66.825 mm^3^ (by around 20 mm^3^ that is about 30%), which shows an increasing with age impact of diminishing in the left HV due to the variable *infs*. It might indicate more sensitivity to infections with age. Similarly, based on Table 3, for the case HV right we got the following: mean (K^*^Age^*^infs) = −61.425 mm^3^ (*K*= −0.91 (mm^3^/year) for females aged 60–75 and the coefficient for the term *infs* for females aged 65–80 equals −69.69 mm^3^. Value −69.69 mm^3^ is <-61.425 mm^3^ (by −8.265 mm^3^ that is about 16%), which shows an increasing with age impact of diminishing in the right HV due to the variable *infs*. It might indicate more sensitivity to infections with age. Thus, association of infections with the left/right HV in our study was stronger in older females.

### 3.3 Other infection types

Four additional types were considered and added for analysis: acute infections, Influenza and Pneumonia, herpesviral infections, and Mycoses (Supplementary material, Infection types; Supplementary Tables 17, 18). We found that difference in the left HV between women aged 65–80 years at the time of MRI imaging with and without history of acute infections: with *p*-value = 3.68e-02, estimated difference equals −106 mm^3^, and 95% confidence interval (CI): (−206, −7) (mm^3^) (Supplementary Figure 4; Supplementary Tables 19–21). Notice that the effect size for acute infections −106 mm^3^ is bigger by absolute value than effect size for all infections −85 mm^3^ in left HV for women aged 65–80 (Table 2). No statistically significant difference in the left/right HV was found between males aged 60–75 or 65–80 years with and without history of other infection types and for other cases for women except for the case of left HV in women aged 65–80 (for example, Supplementary Figures 5–7; Supplementary Tables 20–22).

### 3.4 Infections and APOE4

Recently, the emerging relationship between *APOE* and viral infection has been reported (Chen et al., 2023). So, in our study, five infection types were considered along with *APOE4* for regression analysis: infections (Table 1), acute infections, Influenza and Pneumonia, herpesviral infections, and Mycoses (Supplementary material, Infection types; Supplementary Tables 17, 18). Note that for herpesviral infection and mycoses there was not enough data for analysis: the number of *APOE4* carrier subjects with the history of infection was too small.

We found that the following four groups Infs_APOE4 (subjects who belong to Infs and have APOE4), Infs_noAPOE4 (subjects who belong toInfs and don't have APOE4), noInfs_APOE4 (subjects who do not belong to Infs and have APOE4), and noInfs_noAPOE4 (subjects who do not belong to Infs and don't have APOE4) had different (with ANOVA *p*-value = 2.79e-02) left HV in women aged 65–80 (Supplementary Figure 8B). Though, comparison between groups (Tukey's test) did not reach statistical significance (Supplementary Table 25). For all other cases, no statistically significant difference (ANOVA for four groups) was found (see, for example, Supplementary Figures 8–11; Supplementary Tables 23–30). We additionally compared the age distributions between *Infs_APOE4, Infs_noAPOE4, noInfs_APOE4*, and *noInfs_noAPOE4* groups (Supplementary Tables 31, 32) and found that the differences between mean ages in groups may be and may not be significant for males and females aged 60–75 and 65–80 years. Females aged 65–80 were about 1 year older in the group *Infs_noAPOE4* than in the group *noInfs_APOE4*, 8 months older in the group *Infs_noAPOE4* than in the group *noInfs_noAPOE4*, and 5 months younger in the group *Infs_noAPOE4* than in the group *noInfs_noAPOE4*. Note that age differences might decrease the HV in *Infs_noAPOE4* group compared with *noInfs_APOE4* group, might decrease the HV in *Infs_noAPOE4* group compared with *noInfs_noAPOE4* group, might increase the HV in *noInfs_APOE4* group compared with *noInfs_noAPOE4* group.

For the case of all infections, the result of regression analysis for the left HV for women aged 60–75 (note that ANOVA *p*-value = 9.92e-02, see Supplementary Figure 8A) is presented in Supplementary Table 33 The best significant model did not indicate any dependency on history of infections. The regression coefficients showed that the left HV for women aged 60–75 decreased with age only (Supplementary Table 34). The result of regression analysis for the left HV for women aged 65–80 (note that ANOVA *p*-value = 2.79e-02, see Supplementary Figure 8B) is presented in Supplementary Table 35. The best significant model did not indicate any dependency on history of infections. The regression coefficients showed that the left HV for women aged 65–80 decreased with age and for APOE4 carriers (Supplementary Table 36). One of the reasons that the best regression model did not depend on history of infections could be related to diminishing of the number of subjects by 16% when taking *APOE4* data into consideration. See also the result of regressions analysis for the left HV for men aged 60–75 and 65–80 (Supplementary Tables 37–40).

Overall, for five infection types considered in this study, the result of regression analysis for females/males aged 60–75 showed that the left/right HV depended only on age. The result of regression analysis for males aged 65–80 showed that the left/right HV depended only on age. The result of regression analysis for females aged 65–80 showed that the left/right HV depended on both age and *APOE4* status. Our regression analysis results showed the relationship between the HV and *APOE4* status in older women aged 65+, which was reported in literature (see, for example, Veldsman et al., 2021).

## 4 Discussion

Our study found significant differences in HV between women with and without history of infectious diseases, in both age groups (60–75 and 65–80 years). Women, who were diagnosed with infectious disease(s) between 2009 and 2014, had significantly lower HV later in life, compared to women of the same ages without respective diagnoses. These results support a major role of infections in neurodegeneration in females. For men, the associations between infections and HV did not reach a statistical significance. The observed sex difference may reflect a higher vulnerability of female's brain to infection-related factors, which potentially could contribute to the higher risk of AD in women compared to men. This deserves future investigation.

Note that we included only individuals aged 60 years and older in the analysis. A recent study by Muzambi et al. (2022), which also used UK Biobank data, did not find a significant association between prior infections and HV. However, comparing to our study, they selected a substantially younger cohort with neuroimaging measures for their analysis (55 years, both mean and median age, with 90% aged between 40 and 65, and ~70% between 40 and 60 years, at time of neuroimaging visit). This potentially could contribute to the lack of association. Indeed, younger people have generally better immunity, and their brains may be less vulnerable to infection-related damage, comparing to older adults, whose brains are more vulnerable to infections due to immunosenescence (Pawelec et al., 2020). Considering this possibility, we focused our analysis on older adults aged 60+. Notably, association of infections with HV in our study was stronger for older participants. The larger effect of prior infections on HV seen in older individuals (the last section in Section 3.2) is in line with decreasing brain resilience to stressors (including infection-related) during aging (Ukraintseva et al., 2021).

According to study (Schuff et al., 1999), where healthy subjects (cognitively normal for their age and had no signs of mild cognitive impairment or early dementia) aged 36–85 participated, the estimated HV loss per year was about 14.6 mm^3^/year. In contrast with Schuff et al. (1999), in this study, elderly subjects aged 60–75 were considered, including both healthy and unhealthy subjects. These two factors, age and healthy/unhealthy status, might increase the loss rate of HV up to about 30 mm^3^/year as estimated in this study. One must also remark that different head-size correction (normalization) strategies are not interchangeable and may yield various volumetric results (Arndt et al., 1991; Mathalon et al., 1993; Goldstein et al., 1999; Seidman et al., 1999; Sanfilipo et al., 2004; Barnes et al., 2010; O'Brien et al., 2011; Voevodskaya et al., 2014).

In our study, the effect size increased with age faster for the left vs. right HV. This fact deserves additional discussion. In AD, neuropathological changes generally produce disorders of memory, executive function and other functions, such as language, semantic knowledge, abstract thinking, attention, and visuospatial abilities. Based on Bonner-Jackson et al. (2015) and Ezzati et al. (2016), the observed difference between the left and the right HV might be interpreted as a more accelerated degradation of verbal memory compared with visual-spatial memory in older women. In contrast, it is reported that normal aging—for participants having no present/past history of any neurological, psychological problems and or sensory deficits—affects both the verbal and visual working memory in a relatively similar way (Kumar and Priyadarshi, 2013). The hippocampus participates in the encoding, consolidation, and retrieval of memories, including working memory (Gazzaniga et al., 2014). So, based on this study, the presence of infectious diseases might be one of the factors that changes the pattern of the “verbal versus visual-spatial” decline with aging.

One should note that though atrophy in the hippocampus is one of the key factors in the process of age-related memory loss and dementia, it might not be solely attributable to AD-related pathology. The extension of the association between infections and HV size, confirmed in this study, to the case of AD is not straightforward because the association between infections and AD is complex *per se* (Maheshwari and Eslick, 2015). Using a plausible solution of treating infections using antibiotics (hence, supposedly, treating AD) is conflicting (Loeb et al., 2004; Molloy et al., 2013). At the same time, mounting evidence suggests that neurodegenerative disorder (such as AD) could be caused by inflammatory immune responses to pathogens that afflict brain tissue (Kinney et al., 2018; Hussein, 2021). With hypothesis of a sustained immune response in the brain, gradually emerging as a third core pathology in AD (along with amyloid-β plaques and neurofibrillary tangles), the link between infections and HV/AD might help narrowing down the research. Conducting large-scale analysis comprising data for hundreds of thousands of individuals, including UKB (UK Biobank, 2023) used in this study) makes HV estimates more precise, reliable, and statistically based.

We acknowledge several study limitations. First, although we investigated about twenty covariates relevant to AD (Supplementary Tables 1, 2), the set of such covariates may be further expanded based on new emerging evidence from the literature. Next, our analysis considered only two age intervals (60–75, and 65–80 years) due to limited UKB subsample of eligible older participants with MRI data. Also, in this study, we evaluated regression models using the Akaike information criterion. One should note, however, that there is no universal procedure by which one can determine the “best model”. In our study, we applied the AIC approach calculating goodness-of-fit and model variability in order to select the most parsimonious model (Burnham and Anderson, 2002; Anderson, 2008; Burnham et al., 2011). Finally, the UK Biobank is volunteer-based study, and so it may not represent general population, therefore, results obtained using this sample should not be extrapolated to the entire UK population, or to other populations, and need further confirmation in additional research.

## 5 Conclusion

In summary, our findings support connection between infections and neurodegeneration in older women. In our study, women aged between 60 and 80 years, with recent history of infectious diseases, had a significantly lower HV, compared to women without such history. The effect size increased with age, in line with increasing brain vulnerability to stressors due to aging-related decline in resilience. Results for males didn't reach a statistical significance. The observed sex difference might reflect a higher vulnerability of female brain to infection-related factors, which, in turn, might contribute to a higher risk of AD observed in women compared to men, which deserves further investigation.

## Data availability statement

Publicly available datasets were analyzed in this study. This data can be found here: The UK Biobank database was used in this study (available online at https://www.ukbiobank.ac.uk).

## Ethics statement

The studies involving humans were approved by Duke University Health System Institutional Review Board. The studies were conducted in accordance with the local legislation and institutional requirements. Written informed consent was obtained by the UKB from the participants in accordance with the UK national legislation and the UKB requirements. The latest (at the time of calculations) available information on participants' withdrawal in UKB was taken into account.

## Author contributions

VP: Conceptualization, Formal analysis, Investigation, Methodology, Software, Visualization, Writing – original draft, Writing – review & editing, Validation. SU: Conceptualization, Formal analysis, Investigation, Methodology, Supervision, Writing – review & editing, Writing – original draft, Project administration. HD: Data curation, Investigation, Writing – review & editing, Validation. KA: Investigation, Methodology, Writing – review & editing. AY: Methodology, Writing – review & editing, Investigation.
