# Supplementary material for: Prior infections are associated with smaller hippocampal volume in older women
Source: Front Dement. Author manuscript; Available in PMC 2024 May 3. (PMC11067727; doi:10.3389/frdem.2024.1297193)
Supplement: Supplement1 [file NIHMS1988418-supplement-Supplement1.docx]

Supplementary Material

# Supplementary Figures


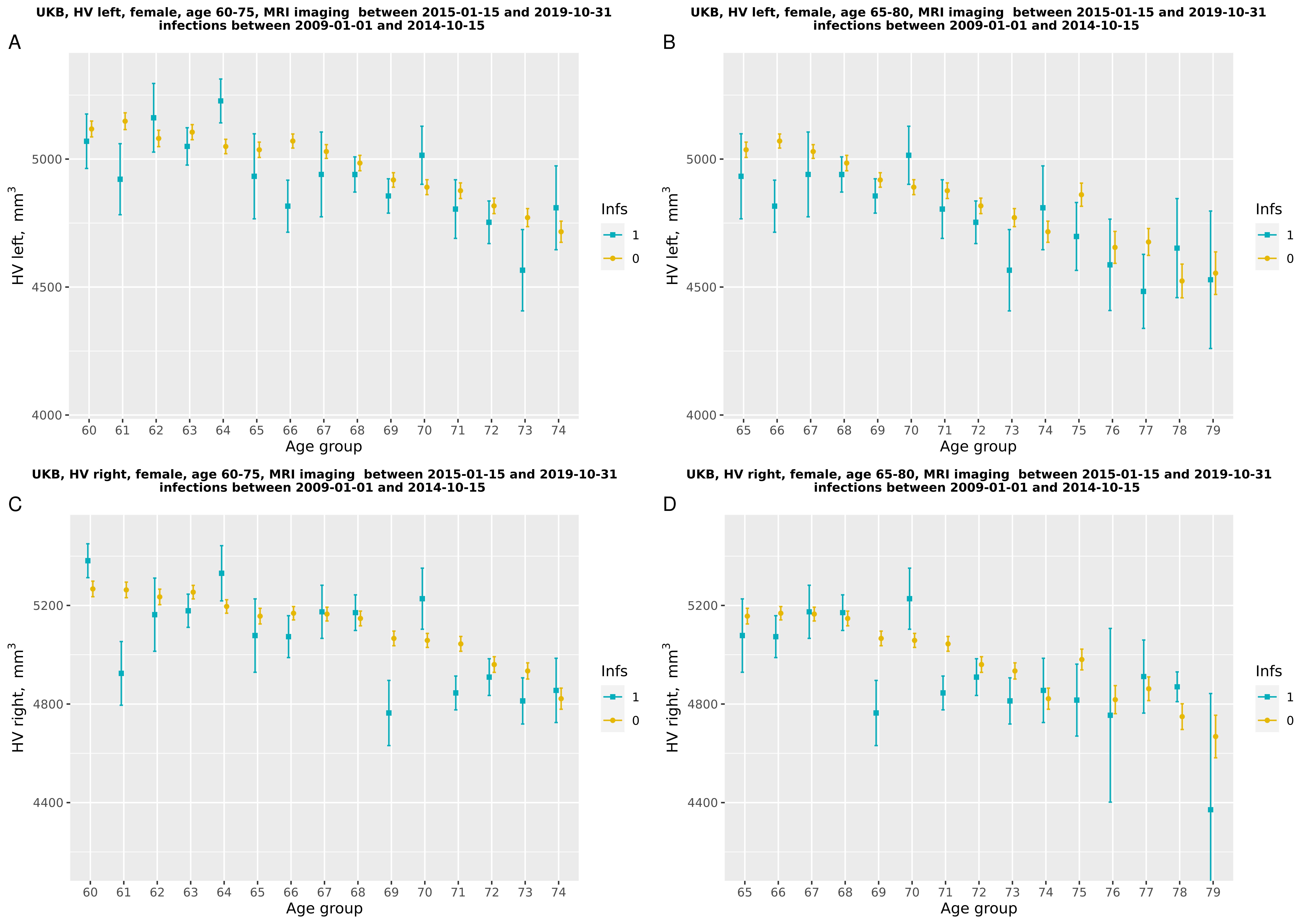


**Supplementary Figure 1**. The left and right HV for females age 60-75 and age 65-80 are shown by one year age groups with (Infs=1) and without history of infection (Infs=0). The HV value presents the mean and the bar presents the standard error in each age group. More detailed information about descriptive statistics of raw data for **(A)**, **(B)**, **(C)**, and **(D)** figures can be found in the Supplementary Table 3, in the Supplementary Table 4, in the Supplementary Table 5, and in the Supplementary Table 6 respectively.


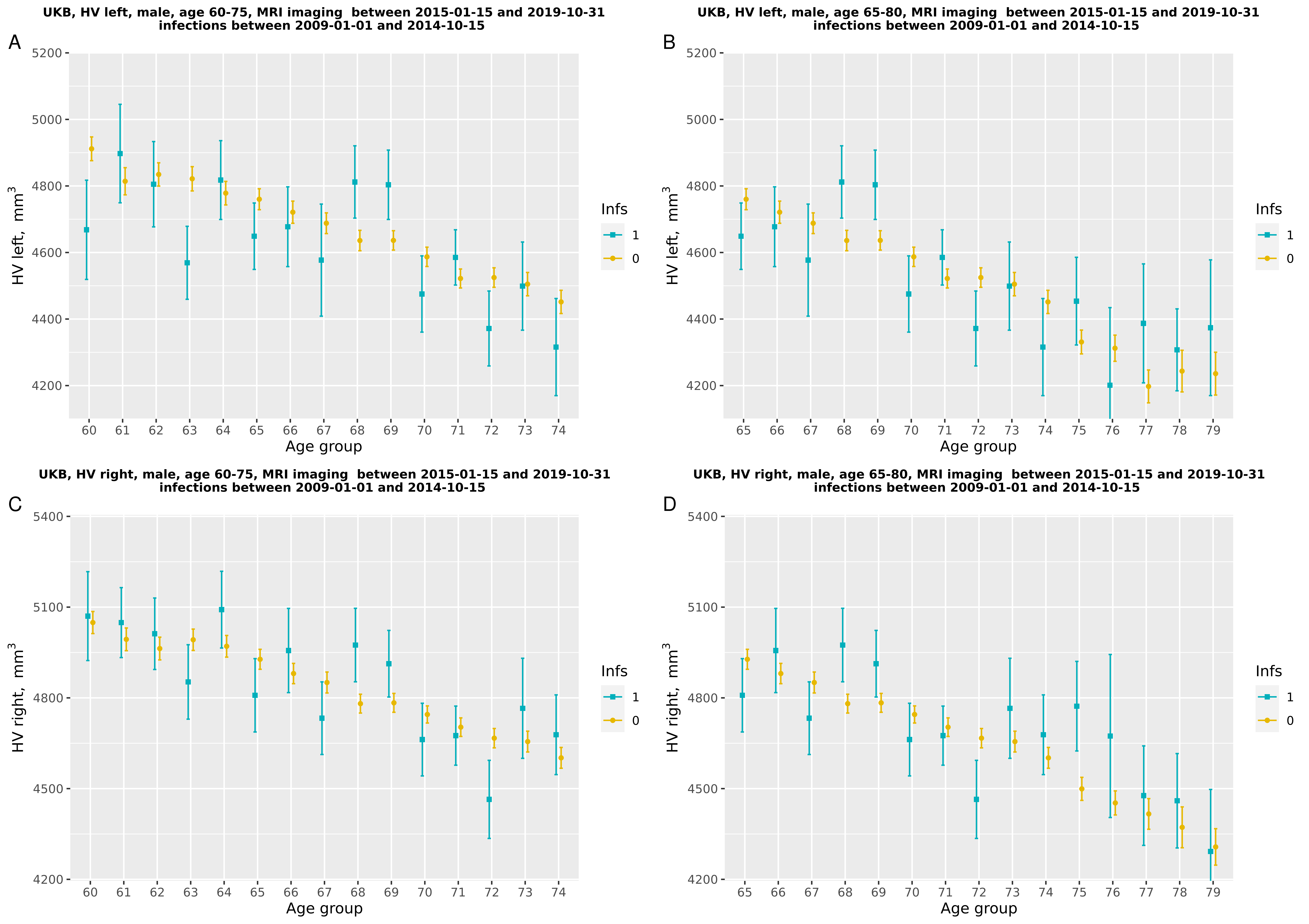


**Supplementary Figure 2**. The left and right HV for males age 60-75 and age 65-80 are shown by one year age groups with (Infs=1) and without history of infection (Infs=0). The HV value presents the mean and the bar presents the standard error in each age group. More detailed information about descriptive statistics of raw data for **(A)**, **(B)**, **(C)**, and **(D)** figures can be found in the Supplementary Table 7, in the Supplementary Table 8, in the Supplementary Table 9, and in the Supplementary Table 10 respectively.


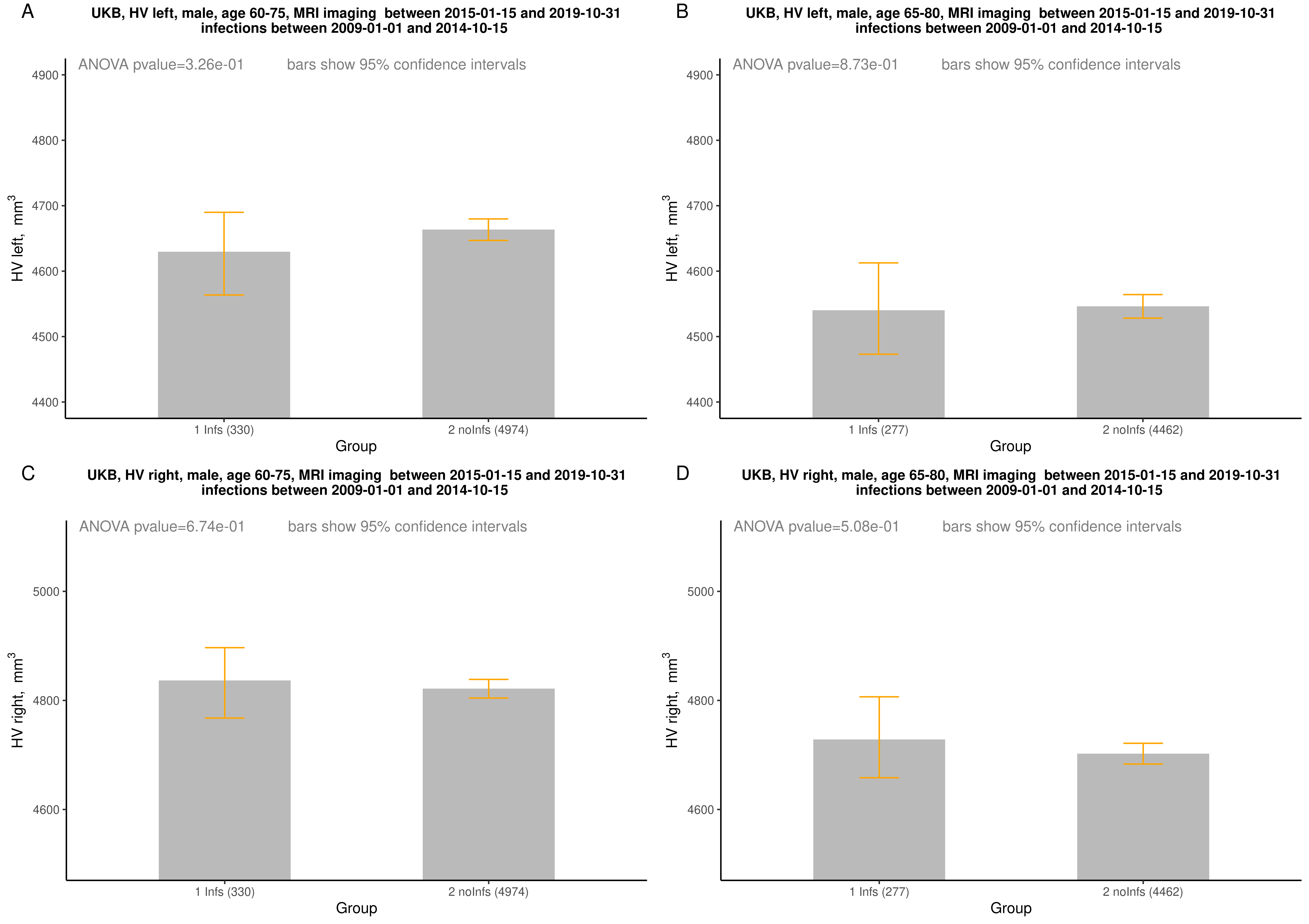


**Supplementary Figure 3**. Comparison of HV (mm^3^) between males with (*Infs*) and without (*noInfs*) history of infectious diseases. Age is age at time of MRI scan. Descriptive statistics for respective groups are shown in subplots: **(A)** UKB, left HV, males, age 60-75. *Infs* (HV: min=2870, max=6169, m=4630, sd=580); no*Infs* (HV: min=1766, max=8108, m=4664, sd=607); **(B)** UKB, left HV, males, age 65-80. *Infs* (HV: min=2870, max=6169, m=4540, sd=571); no*Infs* (HV: min=1636, max=8108, m=4546, sd=611); **(C)** UKB, right HV, males, age 60-75. *Infs* (HV: min=2390, max=7287, m=4837, sd=611); no*Infs* (HV: min=1834, max=8558, m=4822, sd=624); **(D)** UKB, right HV, males, age 65-80. *Infs* (HV: min=2390, max=7287, m=4728, sd=611); no*Infs* (HV: min=1834, max=8558, m=4703, sd=634). For more detailed statistics about **(A)**, **(B)**, **(C)**, and **(D)**, see related to males results in Table 2.


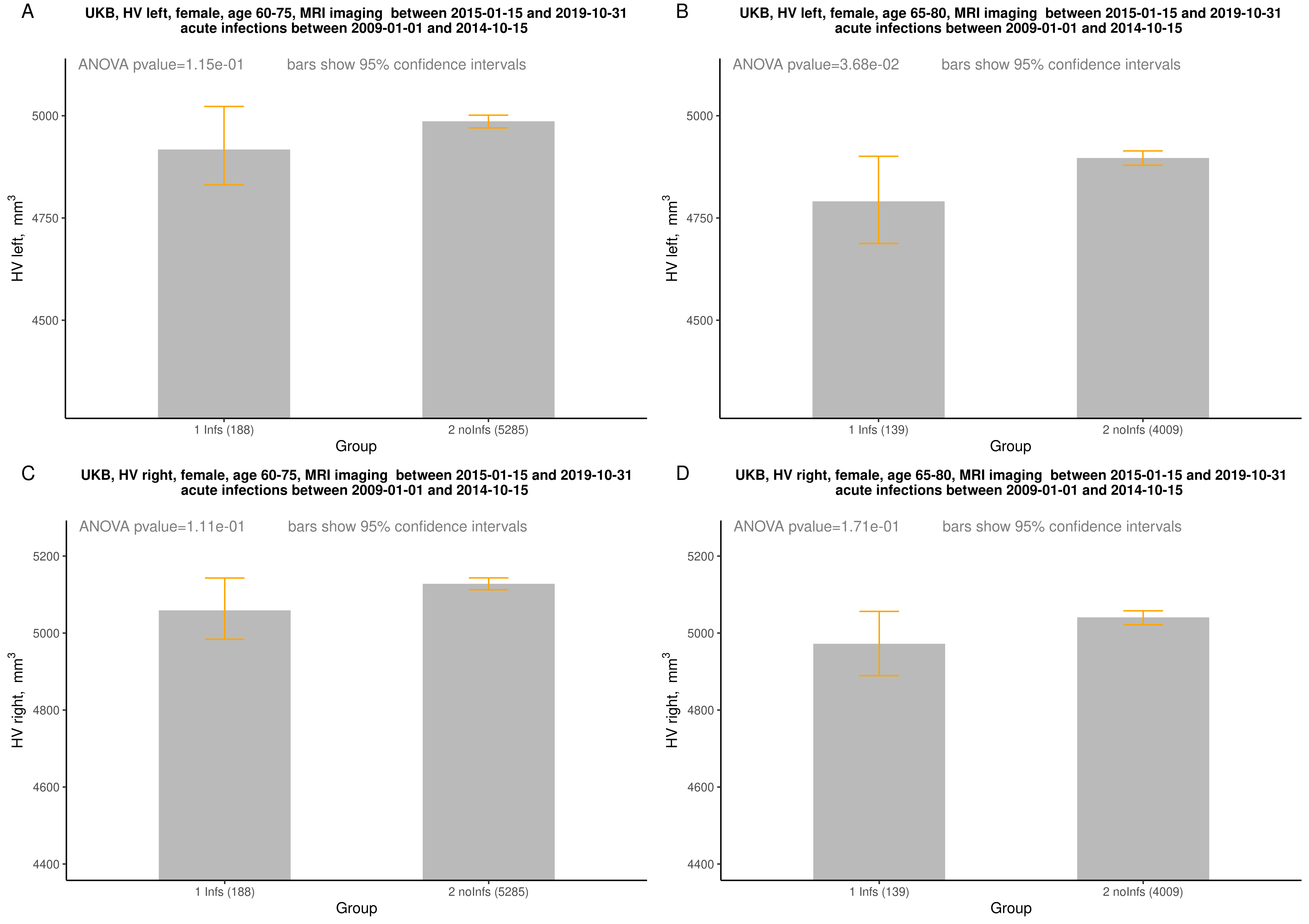


**Supplementary Figure 4.** Comparison of HV (mm^3^) between females with (*Infs*) and without (*noInfs*) history of acute infectious diseases. Age is age at time of MRI scan. Descriptive statistics for respective groups are shown in subplots: **(A)** UKB, left HV, females, age 60-75. Infs (HV: min=2460, max=6238, m=4918, sd=622); noInfs (HV: min=1709, max=10121, m=4986, sd=584); **(B)** UKB, left HV, females, age 65-80. Infs (HV: min=2460, max=6107, m=4791, sd=627); noInfs (HV: min=1709, max=9645, m=4897, sd=587); **(C)** UKB, right HV, females, age 60-75. Infs (HV: min=3228, max=6266, m=5059, sd=552); noInfs (HV: min=1947, max=10063, m=5128, sd=583); **(D)** UKB, right HV, females, age 65-80. Infs (HV: min=3228, max=6162, m=4972, sd=513); noInfs (HV: min=1947, max=8785, m=5041, sd=583). For more detailed statistics about **(A)**, **(B)**, **(C)**, and **(D)**, see related to females results in Supplementary Table 19.


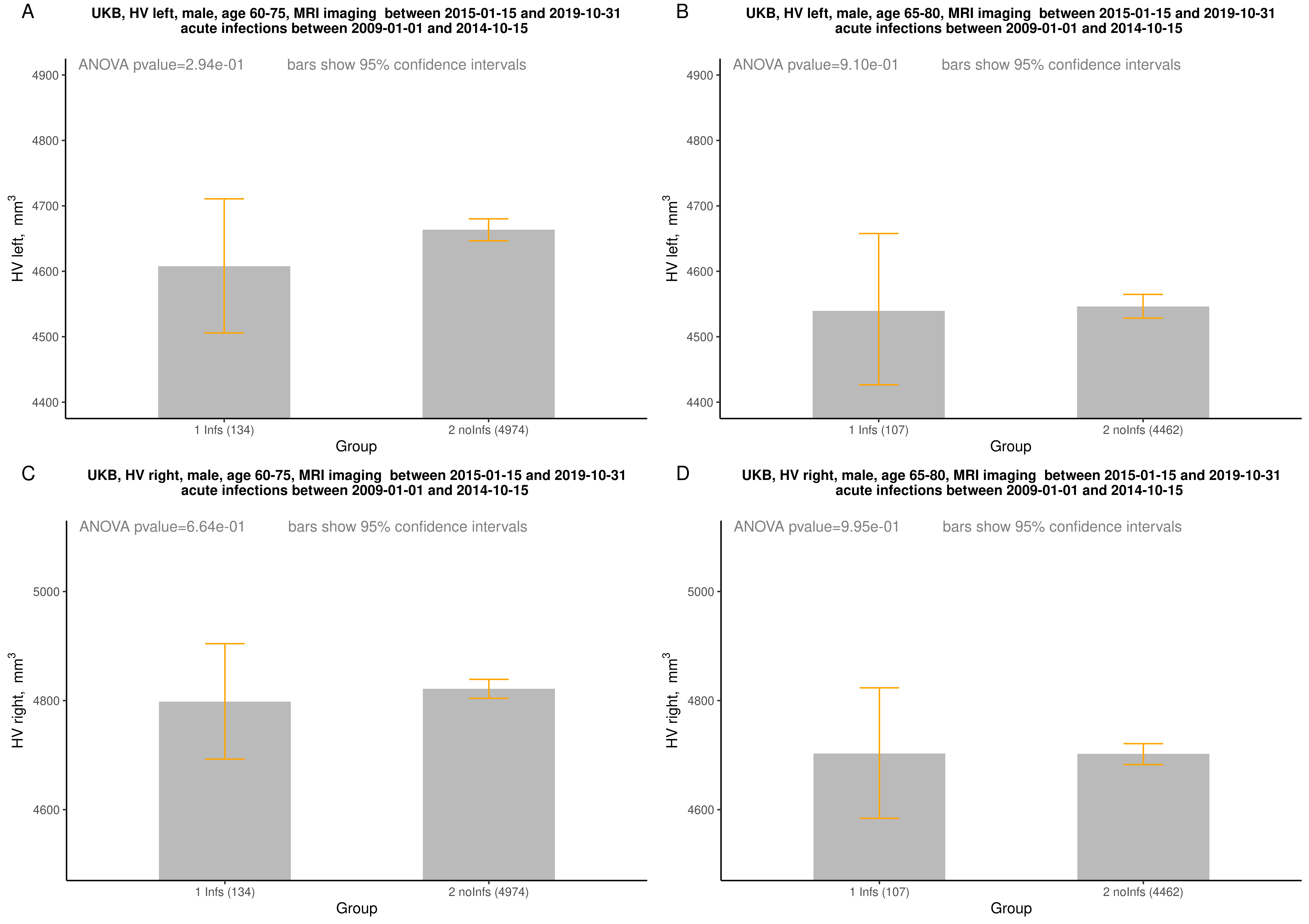


**Supplementary Figure 5**. Comparison of HV (mm^3^) between males with (*Infs*) and without (*noInfs*) history of acute infectious diseases. Age is age at time of MRI scan. Descriptive statistics for respective groups are shown in subplots: **(A)** UKB, left HV, males, age 60-75. Infs (HV: min=2870, max=5812, m=4608, sd=588); noInfs (HV: min=1766, max=8108, m=4664, sd=607); **(B)** UKB, left HV, males, age 65-80. Infs (HV: min=2870, max=5812, m=4539, sd=591); noInfs (HV: min=1636, max=8108, m=4546, sd=611); **(C)** UKB, right HV, males, age 60-75. Infs (HV: min=2783, max=6244, m=4798, sd=605); noInfs (HV: min=1834, max=8558, m=4822, sd=624); **(D)** UKB, right HV, males, age 65-80. Infs (HV: min=2783, max=6192, m=4703, sd=609); noInfs (HV: min=1834, max=8558, m=4703, sd=634). For more detailed statistics about **(A)**, **(B)**, **(C)**, and **(D)**, see related to males results in Supplementary Table 19.


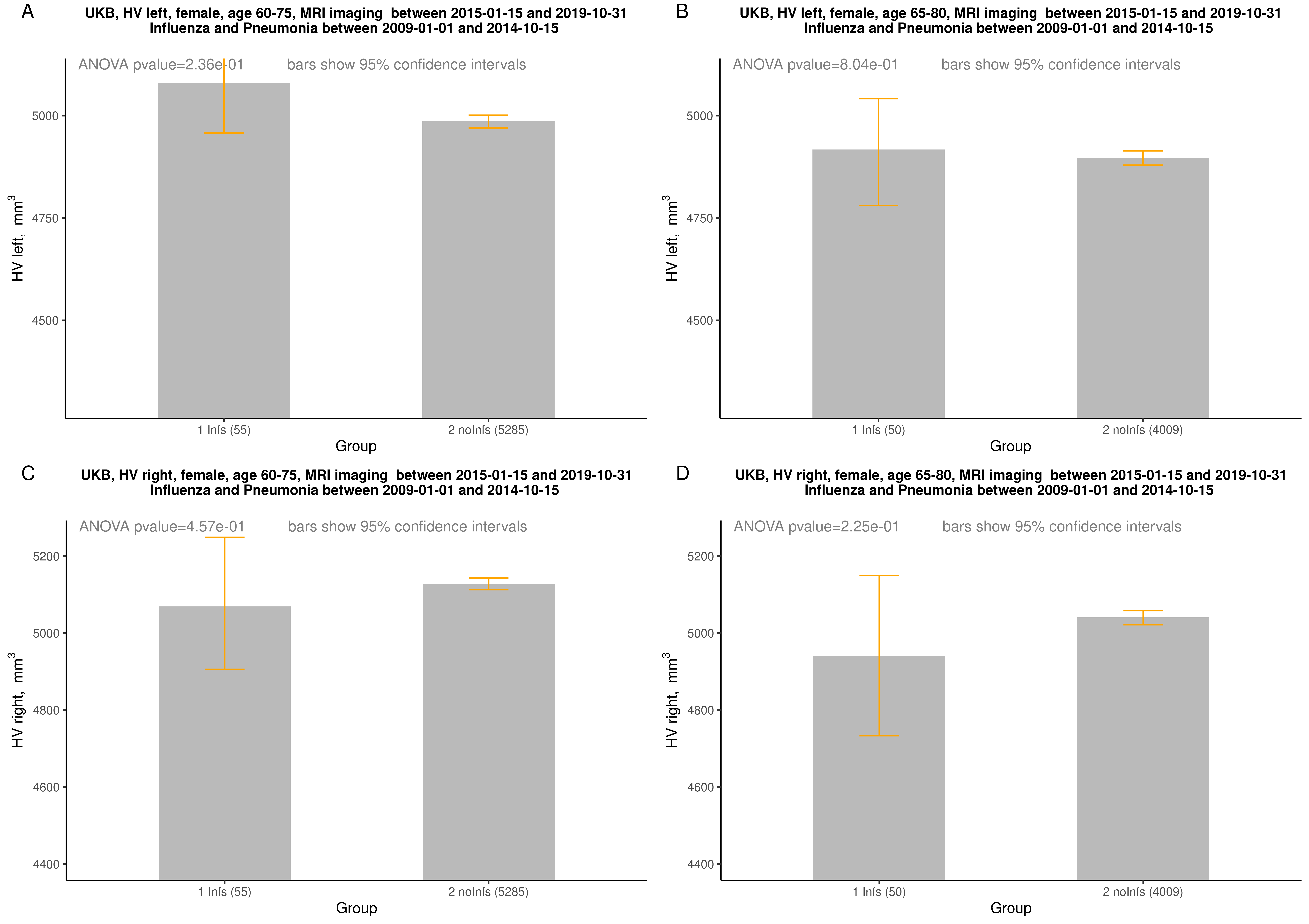


**Supplementary Figure 6**. Comparison of HV (mm^3^) between females with (*Infs*) and without (*noInfs*) history of Influenza and Pneumonia. Age is age at time of MRI scan. Descriptive statistics for respective groups are shown in subplots: **(A)** UKB, left HV, females, age 60-75. Infs (HV: min=4067, max=6045, m=5080, sd=463); noInfs (HV: min=1709, max=10121, m=4986, sd=584); noInfs (HV: min=1766, max=8108, m=4664, sd=607); **(B)** UKB, left HV, females, age 65-80. Infs (HV: min=4030, max=6031, m=4918, sd=490); noInfs (HV: min=1709, max=9645, m=4897, sd=587); noInfs (HV: min=1636, max=8108, m=4546, sd=611); **(C)** UKB, right HV, females, age 60-75. Infs (HV: min=2119, max=6325, m=5069, sd=654); noInfs (HV: min=1947, max=10063, m=5128, sd=583); **(D)** UKB, right HV, females, age 65-80. Infs (HV: min=2119, max=6535, m=4940, sd=725); noInfs (HV: min=1947, max=8785, m=5041, sd=583). For more detailed statistics about **(A)**, **(B)**, **(C)**, and **(D)**, see related to females results in Supplementary Table 22.


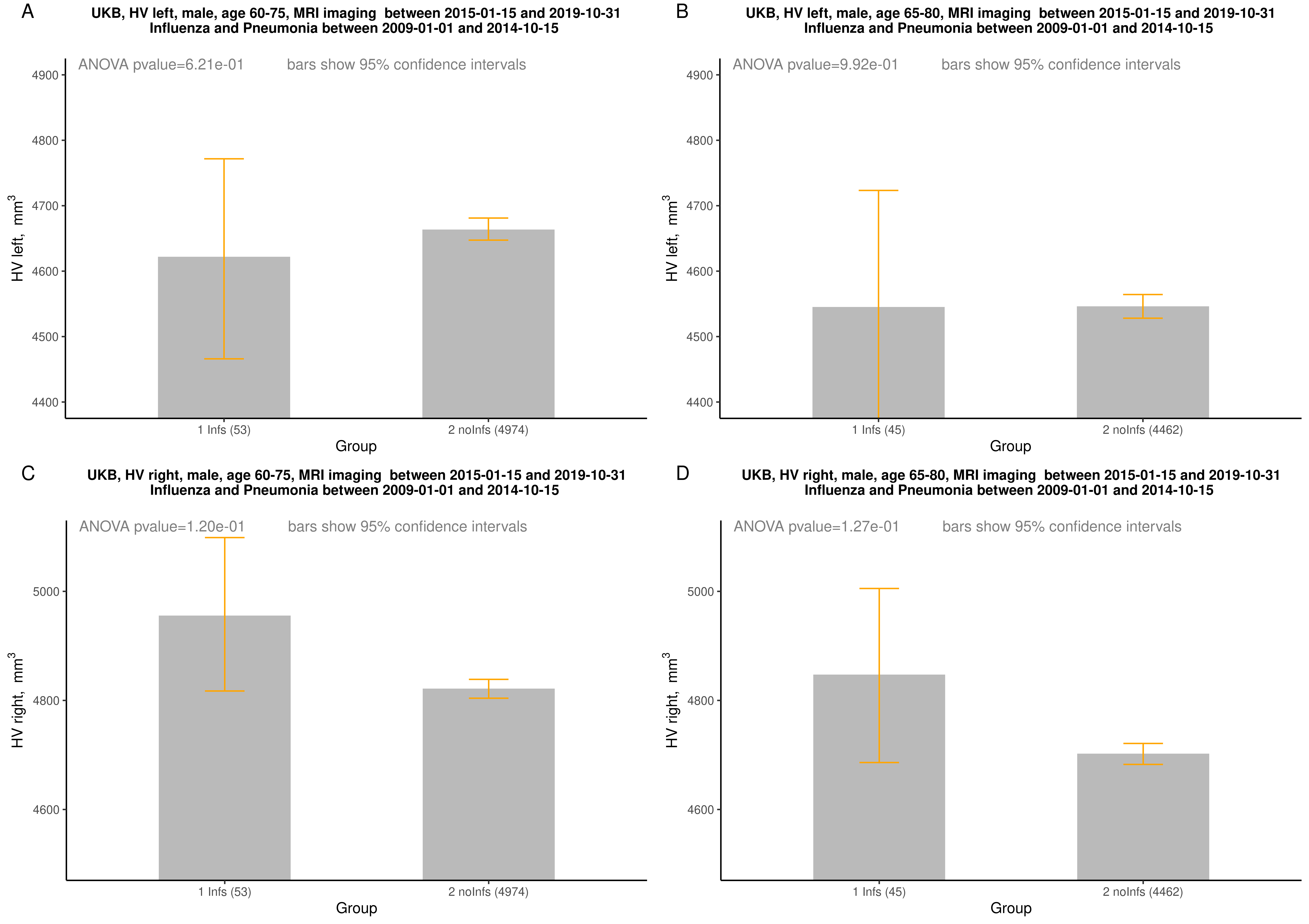


**Supplementary Figure 7**. Comparison of HV (mm^3^) between males with (*Infs*) and without (*noInfs*) history of Influenza and Pneumonia. Age is age at time of MRI scan. Descriptive statistics for respective groups are shown in subplots: **(A)** UKB, left HV, males, age 60-75. Infs (HV: min=3405, max=5554, m=4622, sd=557); noInfs (HV: min=1766, max=8108, m=4664, sd=607); **(B)** UKB, left HV, males, age 65-80. Infs (HV: min=3139, max=5537, m=4545, sd=613); noInfs (HV: min=1636, max=8108, m=4546, sd=611); **(C)** UKB, right HV, males, age 60-75. Infs (HV: min=3593, max=5998, m=4956, sd=523); noInfs (HV: min=1834, max=8558, m=4822, sd=624); **(D)** UKB, right HV, males, age 65-80. Infs (HV: min=3440, max=5998, m=4847, sd=570); noInfs (HV: min=1834, max=8558, m=4703, sd=634). For more detailed statistics about **(A)**, **(B)**, **(C)**, and **(D)**, see related to males results in Supplementary Table 22.


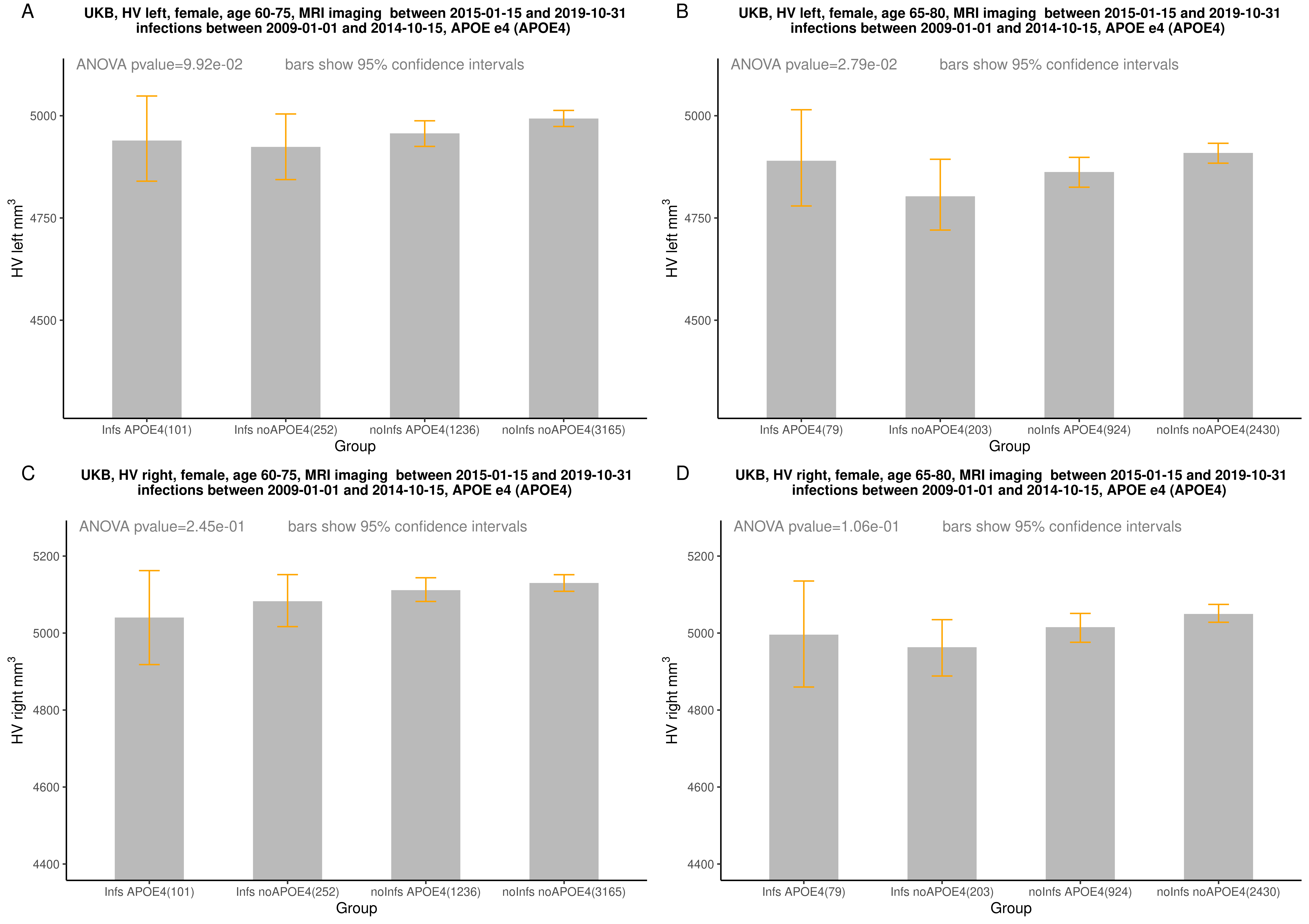


**Supplementary Figure 8.** UKB, left/right HV(mm^3^), females, age 60-75 and 65-80 years. Infs and noInfs correspond to the groups of subjects with history of infection and without history of infection; APOE4 and noAPOE4 correspond to the groups of carriers of APOE e4 allele and non-carriers of APOE e4 allele. **(A)** UKB, left HV(mm^3^), females, age 60-75 years; **(B)** UKB, left HV(mm^3^), females, age 65-80 years; **(C)** UKB, right HV(mm^3^), females, age 60-75 years; **(B)** UKB, right HV(mm^3^), females, age 65-80 years. For more detailed statistics about **(A)**, **(B)**, **(C)**, and **(D)**, see related to females results in Supplementary Tables 23, 25, 24, and 26 respectively.

**
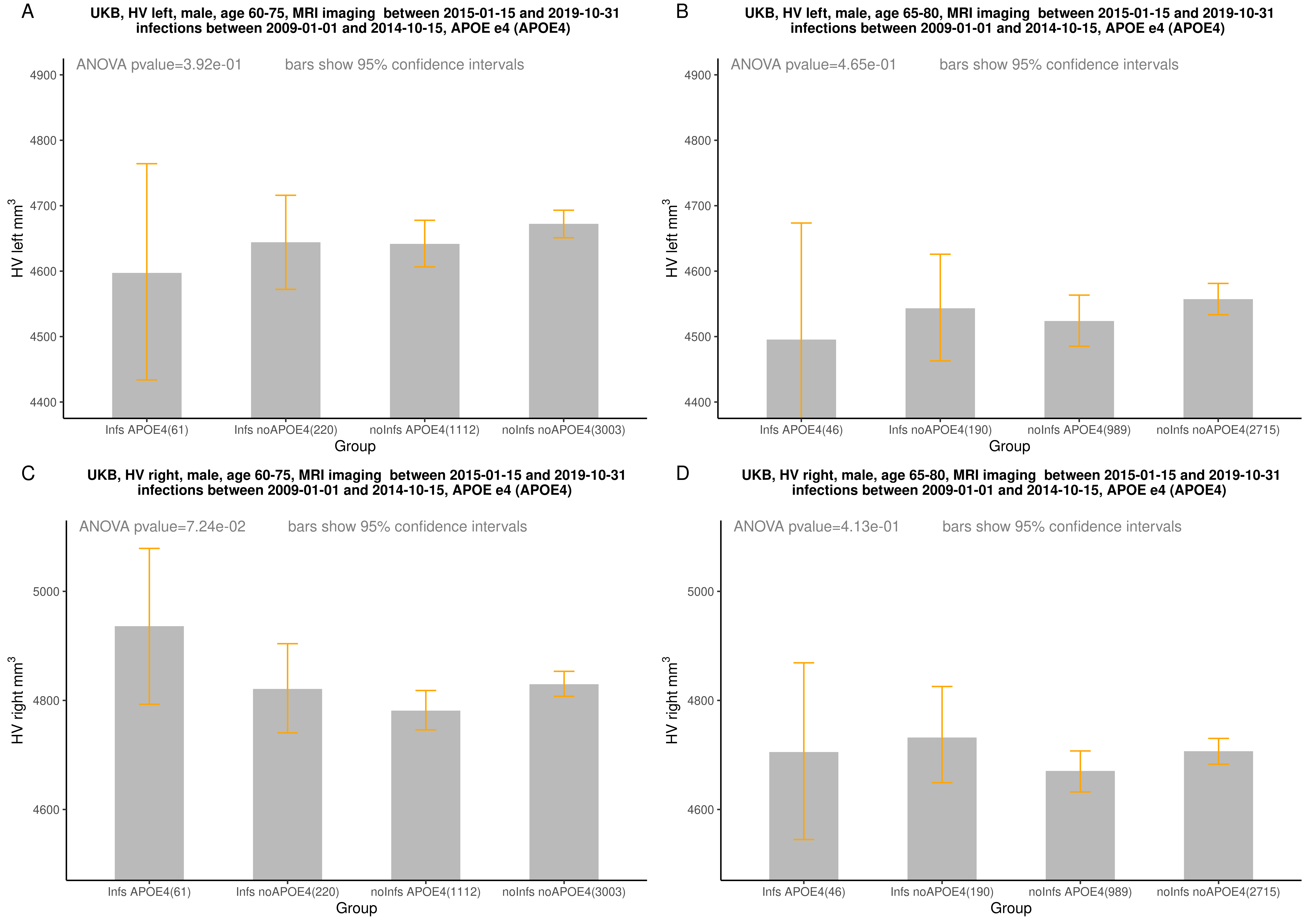
**

**Supplementary Figure 9.** UKB, left/right HV(mm^3^), males, age 60-75 and 65-80 years. Infs and noInfs correspond to the groups of subjects with history of infection and without history of infection; APOE4 and noAPOE4 correspond to the groups of carriers of APOE e4 allele and non-carriers of APOE e4 allele. **(A)** UKB, left HV(mm^3^), males, age 60-75 years; **(B)** UKB, left HV(mm^3^), males, age 65-80 years; **(C)** UKB, right HV(mm^3^), males, age 60-75 years; **(B)** UKB, right HV(mm^3^), males, age 65-80 years. For more detailed statistics about **(A)**, **(B)**, **(C)**, and **(D)**, see related to males results in Supplementary Tables 23, 25, 24, and 26 respectively.


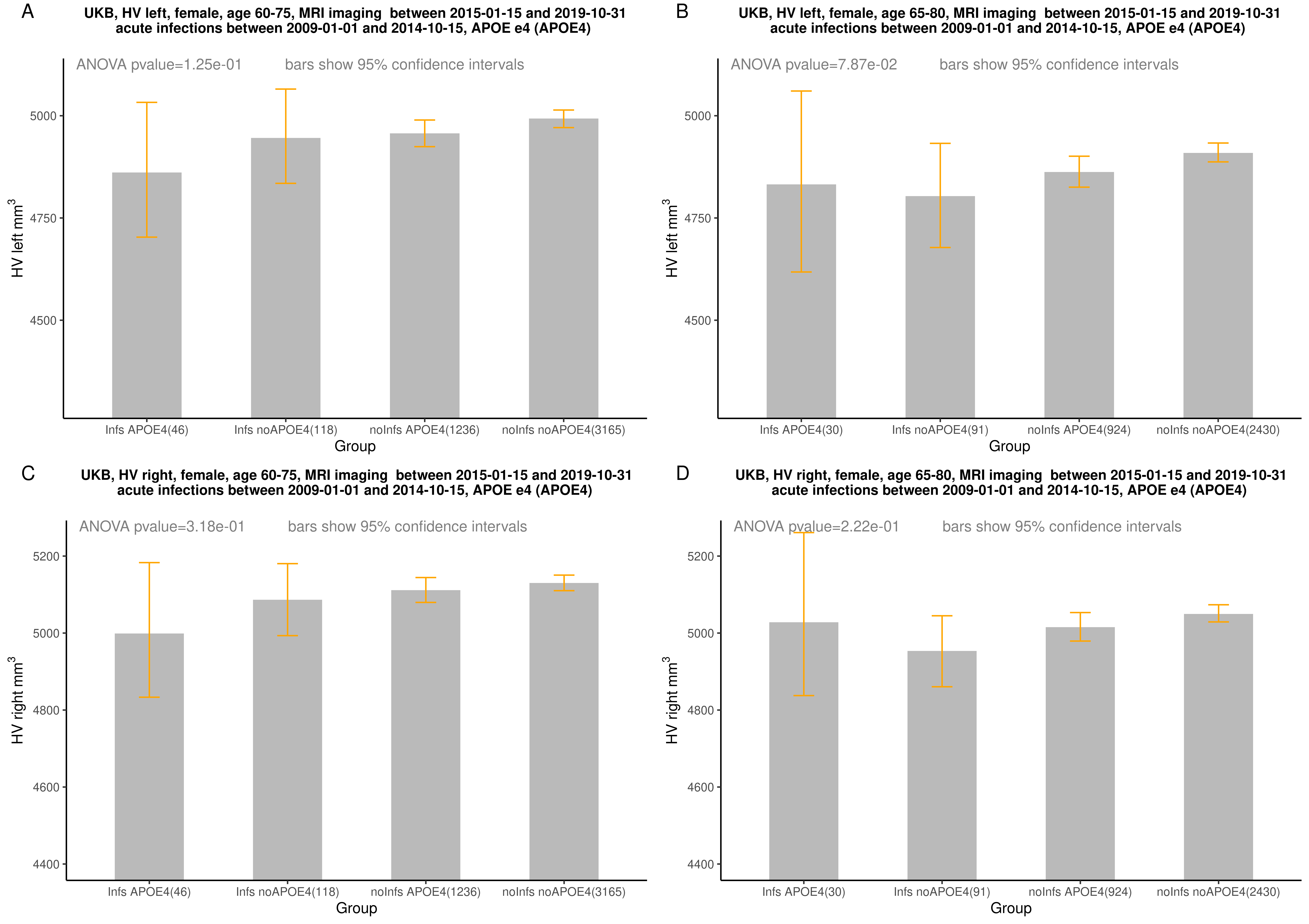


**Supplementary Figure 10.** UKB, left/right HV(mm^3^), females, age 60-75 and 65-80 years. Infs and noInfs correspond to the groups of subjects with history of acute infection and without history of acute infection; APOE4 and noAPOE4 correspond to the groups of carriers of APOE e4 allele and non-carriers of APOE e4 allele. **(A)** UKB, left HV(mm^3^), females, age 60-75 years; **(B)** UKB, left HV(mm^3^), females, age 65-80 years; **(C)** UKB, right HV(mm^3^), females, age 60-75 years; **(D)** UKB, right HV(mm^3^), females, age 65-80 years. For more detailed statistics about **(A)**, **(B)**, **(C)**, and **(D)**, see related to females results in Supplementary Tables 27, 29, 28 and 30 respectively.


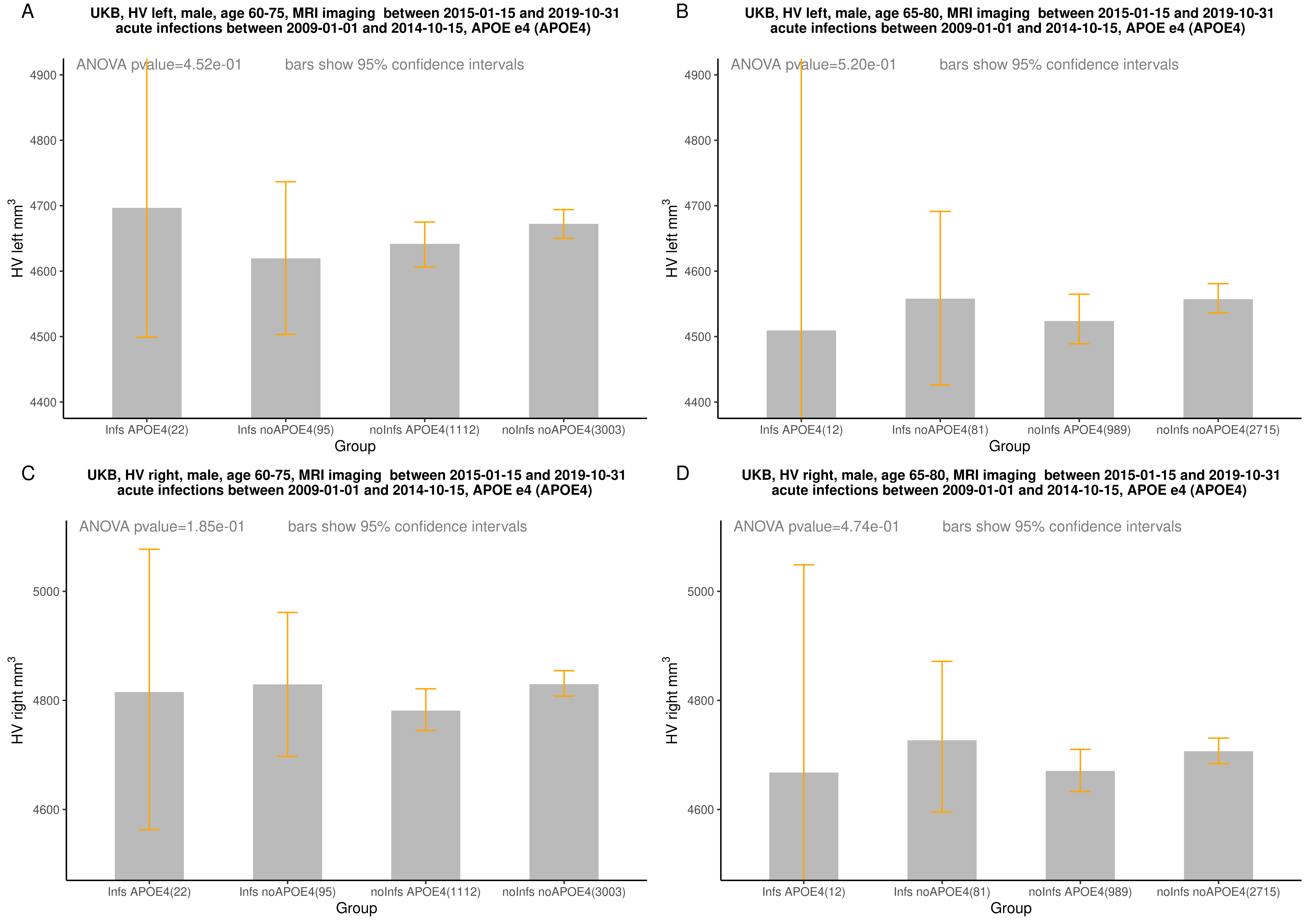


**Supplementary Figure 11.** UKB, left/right HV(mm^3^), males, age 60-75 and 65-80 years. Infs and noInfs correspond to the groups of subjects with history of acute infection and without history of acute infection; APOE4 and noAPOE4 correspond to the groups of carriers of APOE e4 allele and non-carriers of APOE e4 allele. **(A)** UKB, left HV(mm^3^), males, age 60-75 years; **(B)** UKB, left HV(mm^3^), males, age 65-80 years; **(C)** UKB, right HV(mm^3^), males, age 60-75 years; **(D)** UKB, right HV(mm^3^), males, age 65-80 years. For more detailed statistics about **(A)**, **(B)**, **(C)**, and **(D)**, see related to males results in Supplementary Tables 27, 29, 28 and 30 respectively.
