# Supplementary material for: Prior infections are associated with smaller hippocampal volume in older women
Source: Front Dement. Author manuscript; Available in PMC 2024 May 3. (PMC11067727; doi:10.3389/frdem.2024.1297193)
Supplement: Supplement2 [file NIHMS1988418-supplement-Supplement2.docx]

Supplementary Material

# MRI measurements

For all brain six imaging modalities T1, rfMRI, T2_FLAIR, dMRI, SWI, tfMRI, utilized in UK Biobank, the image processing pipeline has also been used to generate many IDPs (image-derived phenotypes). T1 modality provides IDPs primarily relating to volumes of brain tissues and structures, including subcortical structures’ volumes (in particular, hippocampal volumes). In UK Biobank, the left HV (data-field=25019) and the right HV (data-field=25020) are presented as IDPs, which are derived from a T1-weighted structural image during imaging modality T1 (T1-weighted imaging). In order to normalize an IDP for head size, the T1-based “headsize scaling factor” (data-field=25000) should be used. This scaling factor is estimated when transforming from native to standard space. So, to normalize variables for head size, one should multiply raw IDP values by “headsize scaling factor” (Smith et al., 2022). Thus, for our study, we obtained the left/right HV volumes and the “headsize scaling factor” (data-field=25000) from UK Biobank. By multiplying the HV by the “headsize scaling factor”, we normalized the HV.

# Infection types

In this study, in addition to all infections type utilized in the manuscript, we considered the following four infectious diseases types with the following International Classification of Diseases 10th Revision (ICD10) codes occurring during the period from January 1, 2009 to October 15, 2014 (selected to ensure that infectious diseases occurred before MRI visit) were used for the analysis (UK Biobank, 2023; International Statistical Classification of Diseases and Related Health Problems 10th Revision, 2019):

## *Acute infections*

A00-A09 Intestinal infectious diseases

A20-A28 Some zoonotic bacterial disease

A31-A49 Other bacterial diseases

A53-A59 Some infections with sexual mode of transmission

A75-A79 Rickettsioses

A80-A89 Viral and prion infections of the central nervous system

A90-A99 Arthropod-borne viral fevers and viral hemorrhagic fevers

B01-B06 Some of the viral infections manifested on skin and mucous membrane

B15, B16, B17 Acute viral hepatitis A, B, C, other;

B25-B30, B34 Some other viral diseases

(note that it includes:

B25 Cytomegaloviral disease, when clinically manifested

B26 Mumps

B27 - Infectious mononucleosis (mostly herpesviral)

B30 Viral conjunctivitis

B34 other Viral infection, misc. (e.g., Adenovirus))

B50-B64 Protozoal diseases

J09-J18 Influenza and pneumonia

J20-J22 Other acute lower respiratory infections

I30, I33, I40 Acute pericarditis, endocarditis, myocarditis

K35, K85, L04 Acute appendicitis, Acute pancreatitis, Acute lymphadenitis

U07* Covid-19

* In fact, Covid-19 is not relevant to our study because all data was collected before October 31, 2019

## *Influenza (Flu) and Pneumonia*

J09-J18 Influenza and pneumonia

## *Herpesviral infections*

A60 Anogenital herpesviral [herpes simplex] infections

B00 Herpesviral [herpes simplex] infections

B01 Varicella [chickenpox]

B02 Zoster [herpes zoster; shingles]

B10 Other human herpesviruses

B25 Cytomegaloviral disease

B27 Infectious mononucleosis

## Mycoses

B35-B49

# Supplementary Tables

**Supplementary Table 1**. Proportion (%) of those with covariate, or mean value of covariate (see **Note**), in case of history of acute infection (Infs) vs. no history of infection (noInfs), at time of collecting infectious disease data between January 1, 2009 to October 15, 2014, for females, who attended assessment center during imaging visit between January 15, 2015 and October 31, 2019, being aged 60-75 years.

| Covariate | *Infs* | | *noInfs* | P-value |
| --- | --- | --- | --- | --- |
| Age (year) | | 67.57 | 67.18 | 0.06 |
| Weight (kg) | | 69.82 | 69.56 | 0.70 |
| Smoker (yes) | | 64.40 | 59.80 | 0.08 |
| Drinker (yes) | | 96.80 | 97.50 | 0.50 |
| Depression (yes) | | 46.20 | 46.90 | 0.81 |
| Stroke (yes) | | 2.00 | 1.70 | 0.80 |
| Hypertension (yes) | | 27.10 | 24.60 | 0.31 |
| Diabetes (yes) | | 4.70 | 3.80 | 0.49 |
| BMI < 25 | | 45.50 | 43.90 | 0.57 |
| BMI > 30 | | 21.60 | 19.10 | 0.23 |
| HCT <= 35% | | 5.00 | 5.00 | 1.00 |
| T-cell count > 200 | | 100.00 | 100.00 | NA |
| rs2075650(G) | | 27.50 | 27.30 | 0.98 |
| rs6859(A) | | 67.80 | 66.80 | 0.74 |
| rs429358(C) | | 28.6 | 28.1 | 0.88 |
| Education (no, see Note 2) | | 8.40 | 7.40 | 0.57 |
| Sleep disorder (yes) | | 75.50 | 74.20 | 0.75 |
| CancerAll (yes) | | 22.10 | 18.70 | 0.11 |
| PM2.5 (microgram/meter^3^) | | 9.93 | 9.92 | 0.79 |
| PM10 (microgram/meter^3^) | | 16.02 | 16.01 | 0.88 |
| NO (microgram/meter^3^) | | 25.84 | 25.71 | 0.73 |
| NO2 (microgram/meter^3^) | | 43.48 | 42.62 | 0.24 |

**Abbreviations**: body mass index (BMI), hematocrit test level (HCT), cancer, all sites (CancerAll), PM2.5 (particulate matter with median particle diameter ≤ 2.5 micrometer), PM10 (particulate matter with median particle diameter ≤ 10 micrometer), NO (nitric oxide), NO2 (nitrogen dioxide); rs429853 (C)*,* a proxy single nucleotide polymorphism (SNP) for APOE e4 allele; rs6859(A) and rs2075650(G) – SNP variants in *NECTIN2* and *TOMM40* genes, respectively, which are major genetic risk factors for AD and may also be involved in brain vulnerability to infections (Yashin et al. 2018; DOI: 10.1016/j.exger.2017.10.020).

**Note**: For Age (year), Weight (kg), PM2.5 (microgram/meter^3^), PM10 (microgram/meter^3^), NO (microgram/meter^3^), and NO2 (microgram/meter^3^) the corresponding means are shown. Here PM2.5, PM10, NO, NO2 are annual average air pollution concentrations estimates for the year 2010 (Environmental Exposures Metadata, 2023). The numbers are rounded to two decimal places.

**Note 2:** To explore stratification by the level of education/qualification/training, the following seven education/qualification/training levels were considered based on the following levels (Government services and information, 2023; UK Biobank, 2023): 1 - College or University degree, 2 – A-levels or AS levels or equivalent, 3 – O-levels or GCSEs or equivalent, 4 - CSEs or equivalent, 5 - NVQ or HND or HNC or equivalent, 6 - Other professional qualifications, 7 - None of the above. In this study, Education=yes means that the participant has any education/qualification/training described in the levels '1-6', Education=no means that the participant does not belong to any group corresponding to the levels '1-6'.

**Supplementary Table 2.** Proportion (%) of those with covariate, or mean value of covariate (see **Note**), in case of history of infection (*Infs*) vs. no history of infection (*noInfs*) at time of collecting infectious disease data between January 1, 2009 and October 15, 2014, for females who attended assessment center during imaging visit between January 15, 2015 and October 31, 2019, being aged 65-80 years.

| Covariate | *Infs* | no*Infs* | P-value |
| --- | --- | --- | --- |
| Age (year) | 70.85 | 70.56 | 0.16 |
| Weight (kg) | 68.46 | 68.92 | 0.52 |
| Smoker (yes) | 66.40 | 60.30 | 0.04 |
| Drinker (yes) | 96.90 | 97.20 | 0.85 |
| Depression (yes) | 41.40 | 44.00 | 0.40 |
| Stroke (yes) | 1.20 | 2.20 | 0.36 |
| Hypertension (yes) | 32.10 | 27.50 | 0.10 |
| Diabetes (yes) | 5.00 | 4.60 | 0.85 |
| BMI < 25 | 46.70 | 43.90 | 0.35 |
| BMI > 30 | 18.40 | 17.70 | 0.81 |
| HCT <= 35% | 5.10 | 4.20 | 0.50 |
| T-cell count > 200 | 100.00 | 100.00 | NA |
| rs2075650(G) | 27.50 | 26.90 | 0.85 |
| rs6859(A) | 68.70 | 66.20 | 0.40 |
| rs429358(C) | 28.00 | 27.50 | 0.92 |
| Education (no, see Note 2) | 9.00 | 9.30 | 0.96 |
| Sleep disorder (yes) | 75.50 | 72.60 | 0.49 |
| CancerAll (yes) | 21.50 | 20.90 | 0.84 |
| PM2.5 (microgram/meter^3^) | 9.88 | 9.90 | 0.80 |
| PM10 (microgram/meter^3^) | 15.99 | 15.99 | 0.99 |
| NO (microgram/meter^3^) | 25.36 | 25.58 | 0.59 |
| NO2 (microgram/meter^3^) | 42.44 | 42.43 | 0.99 |

**Abbreviations**: body mass index (BMI), hematocrit test level (HCT), cancer, all sites (CancerAll), PM2.5 (particulate matter with median particle diameter ≤ 2.5 micrometer), PM10 (particulate matter with median particle diameter ≤ 10 micrometer), NO (nitric oxide), NO2 (nitrogen dioxide); rs429853 (C)*,* a proxy single nucleotide polymorphism (SNP) for APOE e4 allele; rs6859(A) and rs2075650(G) – SNP variants in *NECTIN2* and *TOMM40* genes, respectively, which are major genetic risk factors for AD and may also be involved in brain vulnerability to infections (Yashin et al. 2018; DOI: 10.1016/j.exger.2017.10.020).

**Note**: For Age (year), Weight (kg), PM2.5 (microgram/meter^3^), PM10 (microgram/meter^3^), NO (microgram/meter^3^), and NO2 (microgram/meter^3^) the corresponding means are shown. Here PM2.5, PM10, NO, NO2 are annual average air pollution concentrations estimates for the year 2010 (Environmental Exposures Metadata, 2023). The numbers are rounded to two decimal places.

**Note 2:** To explore stratification by the level of education/qualification/training, the following seven education/qualification/training levels were considered based on the following levels (Government services and information, 2023; UK Biobank, 2023): 1 - College or University degree, 2 – A-levels or AS levels or equivalent, 3 – O-levels or GCSEs or equivalent, 4 - CSEs or equivalent, 5 - NVQ or HND or HNC or equivalent, 6 - Other professional qualifications, 7 - None of the above. In this study, Education=yes means that the participant has any education/qualification/training described in the levels '1-6', Education=no means that the participant does not belong to any group corresponding to the levels '1-6'.

**Supplementary Table 3**. Descriptive statistics for the left HV(mm^3^) values in females for fifteen age groups (60<=age<61, 61<=age<62, … , 74<=age<=75), each divided by two subgroups with (Infs=1) and without history of infection (Infs=0). The data in this table is presented in the Supplementary Figure 1 (A).

| age(years) | Infs | min(mm^3^) | max(mm^3^) | mean(mm^3^) | sd(mm^3^) | stderror(mm^3^) | number |
| --- | --- | --- | --- | --- | --- | --- | --- |
|  |  |  |  |  |  |  |  |
| 60 | 0 | 3130.51 | 6552.47 | 5117.68 | 583.75 | 30.98 | 355 |
| 60 | 1 | 4150.97 | 5916.67 | 5069.72 | 520.99 | 106.35 | 24 |
| 61 | 0 | 2874.72 | 10120.93 | 5148.07 | 629.62 | 32.73 | 370 |
| 61 | 1 | 3517.34 | 5562.09 | 4921.36 | 604.87 | 138.77 | 19 |
| 62 | 0 | 2372.76 | 6844.11 | 5080.66 | 585.55 | 31.99 | 335 |
| 62 | 1 | 3104.12 | 6237.54 | 5161.41 | 656.95 | 134.10 | 24 |
| 63 | 0 | 2775.10 | 6506.31 | 5105.22 | 551.41 | 29.47 | 350 |
| 63 | 1 | 4130.43 | 5973.89 | 5049.53 | 425.28 | 72.94 | 34 |
| 64 | 0 | 2435.35 | 6722.70 | 5049.20 | 559.77 | 28.20 | 394 |
| 64 | 1 | 3939.15 | 6045.35 | 5227.17 | 461.34 | 85.67 | 29 |
| 65 | 0 | 2699.96 | 7452.56 | 5036.39 | 580.57 | 29.94 | 376 |
| 65 | 1 | 3055.17 | 6106.87 | 4932.72 | 779.16 | 166.12 | 22 |
| 66 | 0 | 2432.15 | 6199.62 | 5070.53 | 557.87 | 27.52 | 411 |
| 66 | 1 | 3296.92 | 5791.87 | 4815.98 | 557.15 | 101.72 | 30 |
| 67 | 0 | 3299.77 | 6704.87 | 5029.54 | 517.25 | 26.89 | 370 |
| 67 | 1 | 2460.15 | 6031.47 | 4940.12 | 776.87 | 165.63 | 22 |
| 68 | 0 | 3128.95 | 9644.65 | 4984.49 | 585.13 | 30.10 | 378 |
| 68 | 1 | 3979.14 | 6063.05 | 4939.87 | 429.38 | 68.76 | 39 |
| 69 | 0 | 3036.18 | 7368.22 | 4918.44 | 568.69 | 28.29 | 404 |
| 69 | 1 | 4084.90 | 5753.32 | 4855.91 | 379.56 | 67.10 | 32 |
| 70 | 0 | 2803.43 | 8907.76 | 4890.25 | 582.19 | 29.18 | 398 |
| 70 | 1 | 4187.62 | 6832.22 | 5014.87 | 567.47 | 113.49 | 25 |
| 71 | 0 | 3025.44 | 6594.99 | 4876.47 | 567.34 | 30.46 | 347 |
| 71 | 1 | 2694.71 | 5814.61 | 4804.67 | 687.07 | 114.51 | 36 |
| 72 | 0 | 2746.56 | 6421.25 | 4817.21 | 528.51 | 30.26 | 305 |
| 72 | 1 | 3940.43 | 5986.69 | 4753.08 | 478.25 | 83.25 | 33 |
| 73 | 0 | 1709.43 | 6307.00 | 4771.33 | 576.44 | 35.28 | 267 |
| 73 | 1 | 2723.01 | 6046.89 | 4565.73 | 745.19 | 158.88 | 22 |
| 74 | 0 | 2811.04 | 6405.61 | 4716.18 | 619.86 | 41.32 | 225 |
| 74 | 1 | 3522.48 | 6104.87 | 4809.65 | 655.12 | 163.78 | 16 |
|  |  |  |  |  |  |  |  |

**Abbreviations**: age – age at the time attending assessment center during imaging visit between January 15, 2015 and October 31, 2019; min, max, mean – the highest, the smallest and the average value of the HV; sd – standard deviation; stderror – standard error; number – the number of the subjects in the subgroup. All numbers relate to the a specific age subgroup. The numbers are rounded to two decimal places.

**Supplementary Table 4.** Descriptive statistics for the left HV(mm^3^) values in females for fifteen age groups (65<=age<66, 66<=age<67, … , 79<=age<=80), each divided by two subgroups with (Infs=1) and without history of infection (Infs=0). The data in this table is presented in the Supplementary Figure 1 (B).

| age(years) | Infs | min(mm^3^) | max(mm^3^) | mean(mm^3^) | sd(mm^3^) | stderror(mm^3^) | number |
| --- | --- | --- | --- | --- | --- | --- | --- |
|  |  |  |  |  |  |  |  |
| 65 | 0 | 2699.96 | 7452.56 | 5036.39 | 580.57 | 29.94 | 376 |
| 65 | 1 | 3055.17 | 6106.87 | 4932.72 | 779.16 | 166.12 | 22 |
| 66 | 0 | 2432.15 | 6199.62 | 5070.53 | 557.87 | 27.52 | 411 |
| 66 | 1 | 3296.92 | 5791.87 | 4815.98 | 557.15 | 101.72 | 30 |
| 67 | 0 | 3299.77 | 6704.87 | 5029.54 | 517.25 | 26.89 | 370 |
| 67 | 1 | 2460.15 | 6031.47 | 4940.12 | 776.87 | 165.63 | 22 |
| 68 | 0 | 3128.95 | 9644.65 | 4984.49 | 585.13 | 30.10 | 378 |
| 68 | 1 | 3979.14 | 6063.05 | 4939.87 | 429.38 | 68.76 | 39 |
| 69 | 0 | 3036.18 | 7368.22 | 4918.44 | 568.69 | 28.29 | 404 |
| 69 | 1 | 4084.90 | 5753.32 | 4855.91 | 379.56 | 67.10 | 32 |
| 70 | 0 | 2803.43 | 8907.76 | 4890.25 | 582.19 | 29.18 | 398 |
| 70 | 1 | 4187.62 | 6832.22 | 5014.87 | 567.47 | 113.49 | 25 |
| 71 | 0 | 3025.44 | 6594.99 | 4876.47 | 567.34 | 30.46 | 347 |
| 71 | 1 | 2694.71 | 5814.61 | 4804.67 | 687.07 | 114.51 | 36 |
| 72 | 0 | 2746.56 | 6421.25 | 4817.21 | 528.51 | 30.26 | 305 |
| 72 | 1 | 3940.43 | 5986.69 | 4753.08 | 478.25 | 83.25 | 33 |
| 73 | 0 | 1709.43 | 6307.00 | 4771.33 | 576.44 | 35.28 | 267 |
| 73 | 1 | 2723.01 | 6046.89 | 4565.73 | 745.19 | 158.88 | 22 |
| 74 | 0 | 2811.04 | 6405.61 | 4716.18 | 619.86 | 41.32 | 225 |
| 74 | 1 | 3522.48 | 6104.87 | 4809.65 | 655.12 | 163.78 | 16 |
| 75 | 0 | 3044.89 | 7042.41 | 4860.85 | 589.70 | 45.23 | 170 |
| 75 | 1 | 4163.82 | 5427.79 | 4697.75 | 496.11 | 132.59 | 14 |
| 76 | 0 | 2636.68 | 6996.14 | 4655.01 | 680.67 | 62.40 | 119 |
| 76 | 1 | 4030.06 | 5212.65 | 4586.83 | 472.37 | 178.54 | 7 |
| 77 | 0 | 3217.98 | 5977.58 | 4676.19 | 542.69 | 52.46 | 107 |
| 77 | 1 | 3731.10 | 5367.07 | 4483.17 | 501.12 | 144.66 | 12 |
| 78 | 0 | 2311.72 | 5821.17 | 4523.78 | 607.79 | 65.92 | 85 |
| 78 | 1 | 3879.67 | 5251.37 | 4652.17 | 474.09 | 193.55 | 6 |
| 79 | 0 | 3079.08 | 5799.73 | 4554.26 | 570.21 | 83.17 | 47 |
| 79 | 1 | 3710.16 | 5215.62 | 4528.46 | 600.53 | 268.57 | 5 |
|  |  |  |  |  |  |  |  |

**Abbreviations**: age – age at the time attending assessment center during imaging visit between January 15, 2015 and October 31, 2019; min, max, mean – the highest, the smallest and the average value of the HV; sd – standard deviation; stderror – standard error; number – the number of the subjects in the subgroup. All numbers relate to the a specific age subgroup. The numbers are rounded to two decimal places.

**Supplementary Table 5.** Descriptive statistics for the right HV(mm^3^) values in females for fifteen age groups (60<=age<61, 61<=age<62, … , 74<=age<=75), each divided by two subgroups with (Infs=1) and without history of infection (Infs=0). The data in this table is presented in the Supplementary Figure 1 (C).

| age(years) | Infs | min(mm^3^) | max(mm^3^) | mean(mm^3^) | sd(mm^3^) | stderror(mm^3^) | number |
| --- | --- | --- | --- | --- | --- | --- | --- |
|  |  |  |  |  |  |  |  |
| 60 | 0 | 2881.76 | 6493.64 | 5267.01 | 595.19 | 31.59 | 355 |
| 60 | 1 | 4690.11 | 6130.73 | 5381.43 | 336.92 | 68.77 | 24 |
| 61 | 0 | 2628.29 | 10063.12 | 5263.06 | 607.63 | 31.59 | 370 |
| 61 | 1 | 3818.85 | 5812.36 | 4924.31 | 562.88 | 129.13 | 19 |
| 62 | 0 | 2058.93 | 7001.11 | 5234.64 | 576.95 | 31.52 | 335 |
| 62 | 1 | 3244.59 | 6136.98 | 5162.56 | 727.43 | 148.49 | 24 |
| 63 | 0 | 3500.48 | 6629.15 | 5253.96 | 514.82 | 27.52 | 350 |
| 63 | 1 | 4386.64 | 6217.49 | 5178.51 | 392.77 | 67.36 | 34 |
| 64 | 0 | 3169.46 | 6569.34 | 5195.84 | 542.61 | 27.34 | 394 |
| 64 | 1 | 3317.97 | 6265.98 | 5330.53 | 603.04 | 111.98 | 29 |
| 65 | 0 | 2431.12 | 8677.90 | 5156.60 | 615.32 | 31.73 | 376 |
| 65 | 1 | 3228.47 | 6005.75 | 5077.73 | 698.02 | 148.82 | 22 |
| 66 | 0 | 3163.98 | 6858.74 | 5168.37 | 549.20 | 27.09 | 411 |
| 66 | 1 | 3830.39 | 6178.52 | 5073.39 | 465.40 | 84.97 | 30 |
| 67 | 0 | 2435.39 | 6416.03 | 5164.99 | 538.72 | 28.01 | 370 |
| 67 | 1 | 4148.99 | 6325.44 | 5174.20 | 505.57 | 107.79 | 22 |
| 68 | 0 | 3134.11 | 8710.10 | 5147.18 | 581.04 | 29.89 | 378 |
| 68 | 1 | 4349.05 | 6061.55 | 5170.73 | 451.00 | 72.22 | 39 |
| 69 | 0 | 2168.07 | 8117.22 | 5066.22 | 596.25 | 29.66 | 404 |
| 69 | 1 | 2118.74 | 5932.15 | 4763.62 | 747.72 | 132.18 | 32 |
| 70 | 0 | 2809.47 | 8785.27 | 5058.13 | 567.06 | 28.42 | 398 |
| 70 | 1 | 4365.60 | 7287.25 | 5227.42 | 619.51 | 123.90 | 25 |
| 71 | 0 | 2857.76 | 6446.02 | 5044.14 | 558.80 | 30.00 | 347 |
| 71 | 1 | 4121.96 | 5602.83 | 4844.91 | 411.58 | 68.60 | 36 |
| 72 | 0 | 2904.17 | 6151.32 | 4960.24 | 553.19 | 31.68 | 305 |
| 72 | 1 | 4069.16 | 5754.71 | 4909.30 | 427.92 | 74.49 | 33 |
| 73 | 0 | 3006.65 | 6256.60 | 4934.22 | 536.24 | 32.82 | 267 |
| 73 | 1 | 3984.69 | 5475.45 | 4812.61 | 439.37 | 93.67 | 22 |
| 74 | 0 | 1946.56 | 5978.62 | 4821.71 | 642.62 | 42.84 | 225 |
| 74 | 1 | 3768.12 | 5659.51 | 4855.34 | 521.52 | 130.38 | 16 |
|  |  |  |  |  |  |  |  |

**Abbreviations**: age – age at the time attending assessment center during imaging visit between January 15, 2015 and October 31, 2019; min, max, mean – the highest, the smallest and the average value of the HV; sd – standard deviation; stderror – standard error; number – the number of the subjects in the subgroup. All numbers relate to the a specific age subgroup. The numbers are rounded to two decimal places.

**Supplementary Table 6.** Descriptive statistics for the right HV(mm^3^) values in females for fifteen age groups (65<=age<66, 66<=age<67, … , 79<=age<=80), each divided by two subgroups with (Infs=1) and without history of infection (Infs=0). The data in this table is presented in the Supplementary Figure 1 (D).

| age(years) | Infs | min(mm^3^) | max(mm^3^) | mean(mm^3^) | sd(mm^3^) | stderror(mm^3^) | number |
| --- | --- | --- | --- | --- | --- | --- | --- |
|  |  |  |  |  |  |  |  |
| 65 | 0 | 2431.12 | 8677.90 | 5156.60 | 615.32 | 31.73 | 376 |
| 65 | 1 | 3228.47 | 6005.75 | 5077.73 | 698.02 | 148.82 | 22 |
| 66 | 0 | 3163.98 | 6858.74 | 5168.37 | 549.20 | 27.09 | 411 |
| 66 | 1 | 3830.39 | 6178.52 | 5073.39 | 465.40 | 84.97 | 30 |
| 67 | 0 | 2435.39 | 6416.03 | 5164.99 | 538.72 | 28.01 | 370 |
| 67 | 1 | 4148.99 | 6325.44 | 5174.20 | 505.57 | 107.79 | 22 |
| 68 | 0 | 3134.11 | 8710.10 | 5147.18 | 581.04 | 29.89 | 378 |
| 68 | 1 | 4349.05 | 6061.55 | 5170.73 | 451.00 | 72.22 | 39 |
| 69 | 0 | 2168.07 | 8117.22 | 5066.22 | 596.25 | 29.66 | 404 |
| 69 | 1 | 2118.74 | 5932.15 | 4763.62 | 747.72 | 132.18 | 32 |
| 70 | 0 | 2809.47 | 8785.27 | 5058.13 | 567.06 | 28.42 | 398 |
| 70 | 1 | 4365.60 | 7287.25 | 5227.42 | 619.51 | 123.90 | 25 |
| 71 | 0 | 2857.76 | 6446.02 | 5044.14 | 558.80 | 30.00 | 347 |
| 71 | 1 | 4121.96 | 5602.83 | 4844.91 | 411.58 | 68.60 | 36 |
| 72 | 0 | 2904.17 | 6151.32 | 4960.24 | 553.19 | 31.68 | 305 |
| 72 | 1 | 4069.16 | 5754.71 | 4909.30 | 427.92 | 74.49 | 33 |
| 73 | 0 | 3006.65 | 6256.60 | 4934.22 | 536.24 | 32.82 | 267 |
| 73 | 1 | 3984.69 | 5475.45 | 4812.61 | 439.37 | 93.67 | 22 |
| 74 | 0 | 1946.56 | 5978.62 | 4821.71 | 642.62 | 42.84 | 225 |
| 74 | 1 | 3768.12 | 5659.51 | 4855.34 | 521.52 | 130.38 | 16 |
| 75 | 0 | 3664.64 | 6473.54 | 4980.64 | 549.21 | 42.12 | 170 |
| 75 | 1 | 4273.05 | 6161.84 | 4815.96 | 546.01 | 145.93 | 14 |
| 76 | 0 | 3091.34 | 8150.73 | 4817.74 | 621.35 | 56.96 | 119 |
| 76 | 1 | 3713.46 | 6534.68 | 4754.37 | 932.24 | 352.36 | 7 |
| 77 | 0 | 3761.13 | 6146.36 | 4861.96 | 497.57 | 48.10 | 107 |
| 77 | 1 | 4161.20 | 6120.83 | 4911.44 | 513.54 | 148.25 | 12 |
| 78 | 0 | 3654.66 | 5661.05 | 4748.86 | 481.04 | 52.18 | 85 |
| 78 | 1 | 4725.50 | 5087.46 | 4870.00 | 147.70 | 60.30 | 6 |
| 79 | 0 | 3141.74 | 5994.91 | 4668.18 | 592.18 | 86.38 | 47 |
| 79 | 1 | 2693.96 | 5217.29 | 4371.27 | 1053.84 | 471.29 | 5 |
|  |  |  |  |  |  |  |  |

**Abbreviations**: age – age at the time attending assessment center during imaging visit between January 15, 2015 and October 31, 2019; min, max, mean – the highest, the smallest and the average value of the HV; sd – standard deviation; stderror – standard error; number – the number of the subjects in the subgroup. All numbers relate to the a specific age subgroup. The numbers are rounded to two decimal places.

**Supplementary Table 7**. Descriptive statistics for the left HV(mm^3^) values in males for fifteen age groups (60<=age<61, 61<=age<62, … , 74<=age<=75), each divided by two subgroups with (Infs=1) and without history of infection (Infs=0). The data in this table is presented in the Supplementary Figure 2 (A).

| age(years) | Infs | min(mm^3^) | max(mm^3^) | mean(mm^3^) | sd(mm^3^) | stderror(mm^3^) | number |
| --- | --- | --- | --- | --- | --- | --- | --- |
|  |  |  |  |  |  |  |  |
| 60 | 0 | 3501.26 | 6188.32 | 4911.69 | 532.75 | 35.68 | 223 |
| 60 | 1 | 3609.51 | 5535.88 | 4668.22 | 577.53 | 149.12 | 15 |
| 61 | 0 | 2661.20 | 6106.49 | 4814.13 | 627.01 | 40.82 | 236 |
| 61 | 1 | 3816.85 | 5615.11 | 4897.40 | 573.11 | 147.98 | 15 |
| 62 | 0 | 2353.37 | 6544.63 | 4834.63 | 585.66 | 34.94 | 281 |
| 62 | 1 | 3281.32 | 5700.17 | 4805.23 | 614.71 | 128.18 | 23 |
| 63 | 0 | 2913.68 | 6863.71 | 4821.53 | 605.41 | 36.44 | 276 |
| 63 | 1 | 3308.37 | 5421.69 | 4569.06 | 525.96 | 109.67 | 23 |
| 64 | 0 | 2180.03 | 6661.03 | 4778.37 | 612.48 | 35.36 | 300 |
| 64 | 1 | 3492.49 | 5675.23 | 4817.68 | 592.38 | 118.48 | 25 |
| 65 | 0 | 2970.60 | 6375.04 | 4760.12 | 559.06 | 31.45 | 316 |
| 65 | 1 | 3731.26 | 5596.96 | 4648.94 | 446.47 | 99.83 | 20 |
| 66 | 0 | 1862.65 | 6134.51 | 4721.14 | 612.51 | 33.32 | 338 |
| 66 | 1 | 3215.10 | 5812.39 | 4677.47 | 587.25 | 119.87 | 24 |
| 67 | 0 | 2582.17 | 6370.42 | 4687.99 | 601.82 | 31.12 | 374 |
| 67 | 1 | 2869.70 | 5490.94 | 4577.10 | 714.36 | 168.38 | 18 |
| 68 | 0 | 2280.24 | 6087.52 | 4635.97 | 589.30 | 30.64 | 370 |
| 68 | 1 | 3424.65 | 5847.85 | 4812.00 | 564.66 | 108.67 | 27 |
| 69 | 0 | 2218.10 | 6101.75 | 4636.40 | 601.41 | 29.10 | 427 |
| 69 | 1 | 3984.04 | 5599.24 | 4803.58 | 429.72 | 104.22 | 17 |
| 70 | 0 | 1919.62 | 6000.40 | 4587.02 | 607.82 | 29.01 | 439 |
| 70 | 1 | 3241.01 | 5739.22 | 4475.28 | 627.51 | 114.57 | 30 |
| 71 | 0 | 1766.33 | 6220.06 | 4521.89 | 585.61 | 28.51 | 422 |
| 71 | 1 | 3764.90 | 5776.55 | 4585.26 | 462.17 | 83.01 | 31 |
| 72 | 0 | 1948.03 | 8108.20 | 4524.75 | 573.36 | 29.26 | 384 |
| 72 | 1 | 3416.23 | 5296.92 | 4371.64 | 528.05 | 112.58 | 22 |
| 73 | 0 | 1775.96 | 6128.05 | 4504.95 | 623.23 | 35.06 | 316 |
| 73 | 1 | 3118.58 | 6168.57 | 4499.04 | 607.07 | 132.47 | 21 |
| 74 | 0 | 2299.47 | 5788.42 | 4451.54 | 575.67 | 34.90 | 272 |
| 74 | 1 | 2879.13 | 5058.90 | 4315.77 | 636.22 | 145.96 | 19 |
|  |  |  |  |  |  |  |  |

**Abbreviations**: age – age at the time attending assessment center during imaging visit between January 15, 2015 and October 31, 2019; min, max, mean – the highest, the smallest and the average value of the HV; sd – standard deviation; stderror – standard error; number – the number of the subjects in the subgroup. All numbers relate to the a specific age subgroup. The numbers are rounded to two decimal places.

**Supplementary Table 8**. Descriptive statistics for the left HV(mm^3^) values in males for fifteen age groups (65<=age<66, 66<=age<67, … , 79<=age<=80), each divided by two subgroups with (Infs=1) and without history of infection (Infs=0). The data in this table is presented in the Supplementary Figure 2 (B).

| age(years) | Infs | min(mm^3^) | max(mm^3^) | mean(mm^3^) | sd(mm^3^) | stderror(mm^3^) | number |
| --- | --- | --- | --- | --- | --- | --- | --- |
|  |  |  |  |  |  |  |  |
| 65 | 0 | 2970.60 | 6375.04 | 4760.12 | 559.06 | 31.45 | 316 |
| 65 | 1 | 3731.26 | 5596.96 | 4648.94 | 446.47 | 99.83 | 20 |
| 66 | 0 | 1862.65 | 6134.51 | 4721.14 | 612.51 | 33.32 | 338 |
| 66 | 1 | 3215.10 | 5812.39 | 4677.47 | 587.25 | 119.87 | 24 |
| 67 | 0 | 2582.17 | 6370.42 | 4687.99 | 601.82 | 31.12 | 374 |
| 67 | 1 | 2869.70 | 5490.94 | 4577.10 | 714.36 | 168.38 | 18 |
| 68 | 0 | 2280.24 | 6087.52 | 4635.97 | 589.30 | 30.64 | 370 |
| 68 | 1 | 3424.65 | 5847.85 | 4812.00 | 564.66 | 108.67 | 27 |
| 69 | 0 | 2218.10 | 6101.75 | 4636.40 | 601.41 | 29.10 | 427 |
| 69 | 1 | 3984.04 | 5599.24 | 4803.58 | 429.72 | 104.22 | 17 |
| 70 | 0 | 1919.62 | 6000.40 | 4587.02 | 607.82 | 29.01 | 439 |
| 70 | 1 | 3241.01 | 5739.22 | 4475.28 | 627.51 | 114.57 | 30 |
| 71 | 0 | 1766.33 | 6220.06 | 4521.89 | 585.61 | 28.51 | 422 |
| 71 | 1 | 3764.90 | 5776.55 | 4585.26 | 462.17 | 83.01 | 31 |
| 72 | 0 | 1948.03 | 8108.20 | 4524.75 | 573.36 | 29.26 | 384 |
| 72 | 1 | 3416.23 | 5296.92 | 4371.64 | 528.05 | 112.58 | 22 |
| 73 | 0 | 1775.96 | 6128.05 | 4504.95 | 623.23 | 35.06 | 316 |
| 73 | 1 | 3118.58 | 6168.57 | 4499.04 | 607.07 | 132.47 | 21 |
| 74 | 0 | 2299.47 | 5788.42 | 4451.54 | 575.67 | 34.90 | 272 |
| 74 | 1 | 2879.13 | 5058.90 | 4315.77 | 636.22 | 145.96 | 19 |
| 75 | 0 | 1636.44 | 6080.42 | 4331.07 | 594.54 | 35.79 | 276 |
| 75 | 1 | 3139.20 | 5537.45 | 4453.83 | 510.16 | 131.72 | 15 |
| 76 | 0 | 2504.19 | 5730.41 | 4312.34 | 560.46 | 39.34 | 203 |
| 76 | 1 | 3114.91 | 4991.07 | 4201.23 | 659.31 | 233.10 | 8 |
| 77 | 0 | 2238.85 | 5493.76 | 4197.53 | 569.17 | 49.35 | 133 |
| 77 | 1 | 3634.12 | 5425.57 | 4387.01 | 592.84 | 178.75 | 11 |
| 78 | 0 | 2709.54 | 6607.66 | 4243.62 | 674.21 | 62.60 | 116 |
| 78 | 1 | 3579.38 | 4601.89 | 4307.46 | 348.19 | 123.10 | 8 |
| 79 | 0 | 2967.90 | 5585.77 | 4235.93 | 560.98 | 64.35 | 76 |
| 79 | 1 | 3800.04 | 5002.62 | 4373.91 | 499.58 | 203.95 | 6 |
|  |  |  |  |  |  |  |  |

**Abbreviations**: age – age at the time attending assessment center during imaging visit between January 15, 2015 and October 31, 2019; min, max, mean – the highest, the smallest and the average value of the HV; sd – standard deviation; stderror – standard error; number – the number of the subjects in the subgroup. All numbers relate to the a specific age subgroup. The numbers are rounded to two decimal places.

**Supplementary Table 9.** Descriptive statistics for the right HV(mm^3^) values in males for fifteen age groups (60<=age<61, 61<=age<62, … , 74<=age<=75), each divided by two subgroups with (Infs=1) and without history of infection (Infs=0). The data in this table is presented in the Supplementary Figure 2 (C).

| age(years) | Infs | min(mm^3^) | max(mm^3^) | mean(mm^3^) | sd(mm^3^) | stderror(mm^3^) | number |
| --- | --- | --- | --- | --- | --- | --- | --- |
|  |  |  |  |  |  |  |  |
| 60 | 0 | 3399.51 | 6702.40 | 5049.06 | 544.63 | 36.47 | 223 |
| 60 | 1 | 3791.26 | 5903.53 | 5070.30 | 569.08 | 146.94 | 15 |
| 61 | 0 | 1911.90 | 6444.69 | 4993.52 | 576.08 | 37.50 | 236 |
| 61 | 1 | 4266.69 | 5958.23 | 5048.83 | 447.84 | 115.63 | 15 |
| 62 | 0 | 2252.39 | 6604.59 | 4963.09 | 629.77 | 37.57 | 281 |
| 62 | 1 | 3455.61 | 6244.12 | 5011.96 | 566.57 | 118.14 | 23 |
| 63 | 0 | 2495.41 | 6264.60 | 4992.12 | 585.59 | 35.25 | 276 |
| 63 | 1 | 3656.15 | 5912.21 | 4852.65 | 589.99 | 123.02 | 23 |
| 64 | 0 | 3178.18 | 7207.52 | 4970.53 | 619.53 | 35.77 | 300 |
| 64 | 1 | 3853.00 | 6238.37 | 5091.68 | 634.07 | 126.81 | 25 |
| 65 | 0 | 2837.50 | 8558.39 | 4927.53 | 586.82 | 33.01 | 316 |
| 65 | 1 | 3423.34 | 6048.17 | 4808.50 | 542.24 | 121.25 | 20 |
| 66 | 0 | 2510.03 | 6558.99 | 4880.45 | 616.55 | 33.54 | 338 |
| 66 | 1 | 3179.19 | 6192.32 | 4956.60 | 682.01 | 139.21 | 24 |
| 67 | 0 | 1833.97 | 6889.59 | 4850.79 | 669.37 | 34.61 | 374 |
| 67 | 1 | 3809.62 | 5668.76 | 4732.80 | 509.39 | 120.07 | 18 |
| 68 | 0 | 2535.57 | 6396.60 | 4780.94 | 598.35 | 31.11 | 370 |
| 68 | 1 | 3170.04 | 5997.85 | 4974.64 | 631.44 | 121.52 | 27 |
| 69 | 0 | 1913.61 | 6922.22 | 4783.59 | 643.87 | 31.16 | 427 |
| 69 | 1 | 4026.98 | 5579.94 | 4912.94 | 453.85 | 110.07 | 17 |
| 70 | 0 | 1953.26 | 6209.07 | 4745.16 | 592.26 | 28.27 | 439 |
| 70 | 1 | 2389.89 | 5806.90 | 4662.08 | 657.85 | 120.11 | 30 |
| 71 | 0 | 2064.51 | 6121.66 | 4703.16 | 630.04 | 30.67 | 422 |
| 71 | 1 | 3324.49 | 5816.87 | 4675.13 | 543.86 | 97.68 | 31 |
| 72 | 0 | 2438.92 | 8283.26 | 4666.76 | 626.60 | 31.98 | 384 |
| 72 | 1 | 2783.17 | 5334.32 | 4464.38 | 605.64 | 129.12 | 22 |
| 73 | 0 | 2366.46 | 6232.10 | 4655.50 | 611.55 | 34.40 | 316 |
| 73 | 1 | 3519.52 | 7286.71 | 4765.53 | 758.00 | 165.41 | 21 |
| 74 | 0 | 2381.43 | 6245.53 | 4601.71 | 566.90 | 34.37 | 272 |
| 74 | 1 | 3040.88 | 5601.87 | 4678.09 | 574.54 | 131.81 | 19 |
|  |  |  |  |  |  |  |  |

**Abbreviations**: age – age at the time attending assessment center during imaging visit between January 15, 2015 and October 31, 2019; min, max, mean – the highest, the smallest and the average value of the HV; sd – standard deviation; stderror – standard error; number – the number of the subjects in the subgroup. All numbers relate to the a specific age subgroup. The numbers are rounded to two decimal places.

**Supplementary Table 10**. Descriptive statistics for the right HV(mm^3^) values in males for fifteen age groups (65<=age<66, 66<=age<67, … , 79<=age<=80), each divided by two subgroups with (Infs=1) and without history of infection (Infs=0). The data in this table is presented in the Supplementary Figure 2 (D).

| age(years) | Infs | min(mm^3^) | max(mm^3^) | mean(mm^3^) | sd(mm^3^) | stderror(mm^3^) | number |
| --- | --- | --- | --- | --- | --- | --- | --- |
|  |  |  |  |  |  |  |  |
| 65 | 0 | 2837.50 | 8558.39 | 4927.53 | 586.82 | 33.01 | 316 |
| 65 | 1 | 3423.34 | 6048.17 | 4808.50 | 542.24 | 121.25 | 20 |
| 66 | 0 | 2510.03 | 6558.99 | 4880.45 | 616.55 | 33.54 | 338 |
| 66 | 1 | 3179.19 | 6192.32 | 4956.60 | 682.01 | 139.21 | 24 |
| 67 | 0 | 1833.97 | 6889.59 | 4850.79 | 669.37 | 34.61 | 374 |
| 67 | 1 | 3809.62 | 5668.76 | 4732.80 | 509.39 | 120.07 | 18 |
| 68 | 0 | 2535.57 | 6396.60 | 4780.94 | 598.35 | 31.11 | 370 |
| 68 | 1 | 3170.04 | 5997.85 | 4974.64 | 631.44 | 121.52 | 27 |
| 69 | 0 | 1913.61 | 6922.22 | 4783.59 | 643.87 | 31.16 | 427 |
| 69 | 1 | 4026.98 | 5579.94 | 4912.94 | 453.85 | 110.07 | 17 |
| 70 | 0 | 1953.26 | 6209.07 | 4745.16 | 592.26 | 28.27 | 439 |
| 70 | 1 | 2389.89 | 5806.90 | 4662.08 | 657.85 | 120.11 | 30 |
| 71 | 0 | 2064.51 | 6121.66 | 4703.16 | 630.04 | 30.67 | 422 |
| 71 | 1 | 3324.49 | 5816.87 | 4675.13 | 543.86 | 97.68 | 31 |
| 72 | 0 | 2438.92 | 8283.26 | 4666.76 | 626.60 | 31.98 | 384 |
| 72 | 1 | 2783.17 | 5334.32 | 4464.38 | 605.64 | 129.12 | 22 |
| 73 | 0 | 2366.46 | 6232.10 | 4655.50 | 611.55 | 34.40 | 316 |
| 73 | 1 | 3519.52 | 7286.71 | 4765.53 | 758.00 | 165.41 | 21 |
| 74 | 0 | 2381.43 | 6245.53 | 4601.71 | 566.90 | 34.37 | 272 |
| 74 | 1 | 3040.88 | 5601.87 | 4678.09 | 574.54 | 131.81 | 19 |
| 75 | 0 | 2147.39 | 6706.31 | 4499.18 | 633.01 | 38.10 | 276 |
| 75 | 1 | 3440.32 | 5442.89 | 4772.53 | 573.42 | 148.06 | 15 |
| 76 | 0 | 2055.32 | 6115.85 | 4452.66 | 565.85 | 39.71 | 203 |
| 76 | 1 | 3230.47 | 5753.05 | 4673.90 | 762.21 | 269.48 | 8 |
| 77 | 0 | 2885.57 | 5738.47 | 4416.31 | 584.01 | 50.64 | 133 |
| 77 | 1 | 3540.27 | 5236.29 | 4476.78 | 545.37 | 164.44 | 11 |
| 78 | 0 | 2530.26 | 6952.22 | 4372.02 | 727.04 | 67.50 | 116 |
| 78 | 1 | 3761.85 | 5066.44 | 4459.83 | 441.23 | 156.00 | 8 |
| 79 | 0 | 2839.65 | 5316.99 | 4307.25 | 525.07 | 60.23 | 76 |
| 79 | 1 | 3594.87 | 4846.03 | 4292.28 | 502.18 | 205.01 | 6 |
|  |  |  |  |  |  |  |  |

**Abbreviations**: age – age at the time attending assessment center during imaging visit between January 15, 2015 and October 31, 2019; min, max, mean – the highest, the smallest and the average value of the HV; sd – standard deviation; stderror – standard error; number – the number of the subjects in the subgroup. All numbers relate to the a specific age subgroup. The numbers are rounded to two decimal places.

**Supplementary Table** **11.** Regression models, female/male, age 60-75 and 65-80. Response variable HV=HV (mm^3^) left/right, independent variables: *Age* – age at the time attending assessment center during imaging visit, *infs*=1 (with history of infections), *infs*=0 (without history of infections). For every regression set among all 8 sets (each set corresponding to one of the cases: female/male, age groups 60-75 and 65-80 and left/right HV values), all 8 models (with linear terms and their pairwise interactions) in each regression set were analyzed and presented in ascending order by AIC value. Signf=1 means that all regression coefficient were significant (P-value<0.05) in a specific model, Signf=0 means the opposite. For regression model a short notation used. For instance, HV ~ 1 + *Age* + *infs***Age* denotes a standard regression HV = Intercept + b_1_**Age* + b_2_**infs* + b_12_**Age***infs* where Intercept is a constant called the bias term (or intercept term), b_1_, b_2_, b_12_ are the regression coefficients corresponding to the *Age*, *infs*, *Age***infs* terms in the regression model. The numbers are rounded to two decimal places.

| Regression Model | AIC | Signf |
| --- | --- | --- |
|  |  |  |
| **Females, age 60-75, HV (mm^3^) left** |  |  |
| HV ~ 1 + *Age* + *infs***Age* | 73643.28 | 1 |
| HV ~ 1 + *Age* + *infs* | 73643.37 | 1 |
| HV ~ 1 + *Age* + *infs* + *infs***Age* | 73645.18 | 0 |
| HV ~ 1 + *Age* | 73645.78 | 1 |
| HV ~ 1 + *infs* + *infs***Age* | 73828.68 | 1 |
| HV ~ 1 + *infs***Age* | 73843.67 | 1 |
| HV ~ 1 + *infs* | 73845.09 | 1 |
| HV ~ 1 | 73849.29 | 1 |
| **Females, age 60-75, HV (mm^3^) right** |  |  |
| HV ~ 1 + *Age* + *infs***Age* | 73565.94 | 1 |
| HV ~ 1 + *Age* + *infs* | 73566.07 | 0 |
| HV ~ 1 + *Age* + *infs* + *infs***Age* | 73567.70 | 0 |
| HV ~ 1 + *Age* | 73567.78 | 1 |
| HV ~ 1 + *infs* + *infs***Age* | 73747.22 | 1 |
| HV ~ 1 + *infs***Age* | 73763.37 | 1 |
| HV ~ 1 + *infs* | 73764.74 | 1 |
| HV ~ 1 | 73768.12 | 1 |
| **Females, age 65-80, HV (mm^3^) left** |  |  |
| HV ~ 1 + *Age* + *infs* | 54796.06 | 1 |
| HV ~ 1 + *Age* + *infs***Age* | 54796.24 | 1 |
| HV ~ 1 + *Age* + *infs* + *infs***Age* | 54797.62 | 0 |
| HV ~ 1 + *Age* | 54800.11 | 1 |
| HV ~ 1 + *infs* + *infs***Age* | 54949.32 | 1 |
| HV ~ 1 + *infs***Age* | 54954.75 | 1 |
| HV ~ 1 + *infs* | 54955.60 | 1 |
| HV ~ 1 | 54961.49 | 1 |
| **Females, age 65-80, HV (mm^3^) right** |  |  |
| HV ~ 1 + *Age* + *infs* | 54828.95 | 1 |
| HV ~ 1 + *Age* + *infs***Age* | 54829.06 | 0 |
| HV ~ 1 + *Age* + *infs* + *infs***Age* | 54830.71 | 0 |
| HV ~ 1 + *Age* | 54830.85 | 1 |
| HV ~ 1 + *infs* + *infs***Age* | 54962.87 | 1 |
| HV ~ 1 + *infs***Age* | 54968.14 | 1 |
| HV ~ 1 + *infs* | 54968.83 | 1 |
| HV ~ 1 | 54972.20 | 1 |
| **Males, age 60-75, HV (mm^3^) left** |  |  |
| HV ~ 1 + *Age* | 67335.34 | 1 |
| HV ~ 1 + *Age* + *infs* | 67335.36 | 0 |
| HV ~ 1 + *Age* + *infs***Age* | 67335.38 | 0 |
| HV ~ 1 + *Age* + *infs* + *infs***Age* | 67337.35 | 0 |
| HV ~ 1 + *infs* + *infs***Age* | 67523.58 | 1 |
| HV ~ 1 + *infs***Age* | 67533.88 | 0 |
| HV ~ 1 | 67534.18 | 1 |
| HV ~ 1 + *infs* | 67534.53 | 0 |
| **Males, age 60-75, HV (mm^3^) right** |  |  |
| HV ~ 1 + *Age* | 67495.38 | 1 |
| HV ~ 1 + *Age* + *infs***Age* | 67497.14 | 0 |
| HV ~ 1 + *Age* + *infs* | 67497.18 | 0 |
| HV ~ 1 + *Age* + *infs* + *infs***Age* | 67498.56 | 0 |
| HV ~ 1 + *infs* + *infs***Age* | 67665.70 | 1 |
| HV ~ 1 | 67680.27 | 1 |
| HV ~ 1 + *infs***Age* | 67681.88 | 0 |
| HV ~ 1 + *infs* | 67682.15 | 0 |
| **Males, age 65-80, HV (mm^3^) left** |  |  |
| HV ~ 1 + *Age* | 57950.28 | 1 |
| HV ~ 1 + *Age* + *infs* | 57951.42 | 0 |
| HV ~ 1 + *Age* + *infs***Age* | 57951.45 | 0 |
| HV ~ 1 + *Age* + *infs* + *infs***Age* | 57953.29 | 0 |
| HV ~ 1 + *infs* + *infs***Age* | 58163.82 | 1 |
| HV ~ 1 | 58171.65 | 1 |
| HV ~ 1 + *infs***Age* | 58172.25 | 0 |
| HV ~ 1 + *infs* | 58172.63 | 0 |
| **Males, age 65-80, HV (mm^3^) right** |  |  |
| HV ~ 1 + *Age* | 58141.77 | 1 |
| HV ~ 1 + *Age* + *infs* | 58143.67 | 0 |
| HV ~ 1 + *Age* + *infs***Age* | 58143.70 | 0 |
| HV ~ 1 + *Age* + *infs* + *infs***Age* | 58144.87 | 0 |
| HV ~ 1 + *infs* + *infs***Age* | 58327.34 | 1 |
| HV ~ 1 | 58329.74 | 1 |
| HV ~ 1 + *infs***Age* | 58331.45 | 0 |
| HV ~ 1 + *infs* | 58331.57 | 0 |
|  |  |  |

**Supplementary Table 12.** *Age* distributions at the time of collecting infectious disease data between January 1, 2009 to October 15, 2014 for those attended assessment center during imaging visit between January 15, 2015 and October 31, 2019 at age 60-75 and 65-80. The numbers are rounded to two decimal places.

| Group | min | max | mean | sd | number |
| --- | --- | --- | --- | --- | --- |
| Female, age 60-75 |  |  |  |  |  |
| *Infs* | 60.02 | 74.99 | 67.57 | 4.08 | 407 |
| no*Infs* | 60.00 | 74.99 | 67.18 | 4.11 | 5285 |
| Male, age 60-75 |  |  |  |  |  |
| *Infs* | 60.02 | 74.94 | 67.80 | 4.13 | 330 |
| no*Infs* | 60.00 | 75.00 | 68.02 | 4.04 | 4974 |
| Female, age 65-80 |  |  |  |  |  |
| *Infs* | 65.02 | 79.75 | 70.85 | 3.52 | 321 |
| no*Infs* | 65.01 | 80.00 | 70.56 | 3.60 | 4009 |
| Male, age 65-80 |  |  |  |  |  |
| *Infs* | 65.05 | 79.42 | 71.24 | 3.71 | 277 |
| no*Infs* | 65.01 | 80.00 | 71.20 | 3.66 | 4462 |

**Supplementary Table 13**. Welch Two Sample t-test for two age distributions, with infection (*Infs*) and without infection (no*Infs*), at the time of collecting infectious disease data between January 1, 2009 to October 15, 2014 for those attended assessment center during imaging visit between January 15, 2015 and October 31, 2019 at age 60-75 and 65-80. The numbers are rounded to two decimal places.

| Test | P-value | 95% Confidence interval | Estimate | stderr |
| --- | --- | --- | --- | --- |
|  |  |  |  |  |
| Female, age 60-75 | 0.06 | [-0.02,0.80] | 67.57 : 67.18 | 0.21 |
| Male, age 60-75 | 0.35 | [-0.68,0.24] | 67.80 : 68.02 | 0.23 |
| Female, age 65-80 | 0.16 | [-0.11,0.69] | 70.85 : 70.56 | 0.20 |
| Male, age 65-80 | 0.85 | [-0.41,0.49] | 71.24 : 71.20 | 0.23 |
|  |  |  |  |  |

**Supplementary Table 14.** Regression models, female, age 65-80. Response variable HV=HV (mm^3^) left, independent variables: *Age* – age at the time attending assessment center during imaging visit, *infs*=1 (with history of infections), *infs*=0 (without history of infections), *smoker*=1 (if the subject was a smoker), *smoker*=0 (if the subject was a non-smoker). Per the regression set corresponding to age groups 65-80 and left HV values, all 64 models (with linear terms and their pairwise interactions) in each regression set were analyzed and presented in ascending order by AIC value. Signf=1 means that all regression coefficients were significant (P-value<0.05) in a specific model, Signf=0 means the opposite. For regression model a short notation used. For instance, HV ~ 1 + *Age* + *infs* + *smoker* + *infs***Age* + *smoker***infs* denotes a standard regression equation HV = Intercept + b_1_**Age* + b_2_**infs* + b_3_**smoker* + b_12_**Age***infs* + b_31_**smoker***infs* where Intercept is a constant called the bias term (or intercept term), b_1_, b_2_, b_3_, b_12_, b_31_ are the regression coefficients corresponding to the *Age*, *infs*, *smoker*, *Age***infs*, *smoker***infs* terms in the regression model. The numbers are rounded to two decimal places.

| Regression Model | AIC | Signf |
| --- | --- | --- |
|  |  |  |
| HV ~ 1 + *Age* + *infs* | 54796.06 | 1 |
| HV ~ 1 + *Age* + *infs***Age* | 54796.24 | 1 |
| HV ~ 1 + *Age* + *infs* + *smoker***Age* | 54797.14 | 0 |
| HV ~ 1 + *Age* + *infs* + *smoker* | 54797.18 | 0 |
| HV ~ 1 + *Age* + *infs***Age* + *smoker***Age* | 54797.31 | 0 |
| HV ~ 1 + *Age* + *smoker* + *infs***Age* | 54797.35 | 0 |
| HV ~ 1 + *Age* + *infs* + *infs***Age* | 54797.62 | 0 |
| HV ~ 1 + *Age* + *smoker***infs* | 54797.92 | 1 |
| HV ~ 1 + *Age* + *infs* + *smoker***infs* | 54798.05 | 0 |
| HV ~ 1 + *Age* + *infs***Age* + *smoker***infs* | 54798.21 | 0 |
| HV ~ 1 + *Age* + *infs* + *infs***Age* + *smoker***Age* | 54798.72 | 0 |
| HV ~ 1 + *Age* + *infs* + *smoker* + *infs***Age* | 54798.76 | 0 |
| HV ~ 1 + *Age* + *infs* + *smoker* + *smoker***Age* | 54798.99 | 0 |
| HV ~ 1 + *Age* + *infs* + *smoker***Age* + *smoker***infs* | 54799.11 | 0 |
| HV ~ 1 + *Age* + *infs* + *smoker* + *smoker***infs* | 54799.15 | 0 |
| HV ~ 1 + *Age* + *smoker* + *infs***Age* + *smoker***Age* | 54799.17 | 0 |
| HV ~ 1 + *Age* + *infs***Age* + *smoker***Age* + *smoker***infs* | 54799.30 | 0 |
| HV ~ 1 + *Age* + *smoker* + *infs***Age* + *smoker***infs* | 54799.34 | 0 |
| HV ~ 1 + *Age* + *smoker***Age* + *smoker***infs* | 54799.47 | 0 |
| HV ~ 1 + *Age* + *smoker* + *smoker***infs* | 54799.49 | 0 |
| HV ~ 1 + *Age* + *infs* + *infs***Age* + *smoker***infs* | 54799.62 | 0 |
| HV ~ 1 + *Age* | 54800.11 | 1 |
| HV ~ 1 + *Age* + *infs* + *smoker* + *infs***Age* + *smoker***Age* | 54800.55 | 0 |
| HV ~ 1 + *Age* + *infs* + *infs***Age* + *smoker***Age* + *smoker***infs* | 54800.67 | 0 |
| HV ~ 1 + *Age* + *infs* + *smoker* + *infs***Age* + *smoker***infs* | 54800.71 | 0 |
| HV ~ 1 + *Age* + *infs* + *smoker* + *smoker***Age* + *smoker***infs* | 54800.96 | 0 |
| HV ~ 1 + *Age* + *smoker***Age* | 54801.01 | 0 |
| HV ~ 1 + *Age* + *smoker* | 54801.05 | 0 |
| HV ~ 1 + *Age* + *smoker* + *infs***Age* + *smoker***Age* + *smoker***infs* | 54801.16 | 0 |
| HV ~ 1 + *Age* + *smoker* + *smoker***Age* + *smoker***infs* | 54801.35 | 0 |
| HV ~ 1 + *Age* + *infs* + *smoker* + *infs***Age* + *smoker***Age* + *smoker***infs* | 54802.50 | 0 |
| HV ~ 1 + *Age* + *smoker* + *smoker***Age* | 54802.89 | 0 |
| HV ~ 1 + *smoker* + *infs***Age* + *smoker***Age* | 54859.58 | 1 |
| HV ~ 1 + *infs* + *smoker* + *smoker***Age* | 54859.72 | 1 |
| HV ~ 1 + *smoker* + *infs***Age* + *smoker***Age* + *smoker***infs* | 54861.30 | 0 |
| HV ~ 1 + *infs* + *smoker* + *infs***Age* + *smoker***Age* | 54861.38 | 0 |
| HV ~ 1 + *infs* + *smoker* + *smoker***Age* + *smoker***infs* | 54861.46 | 0 |
| HV ~ 1 + *infs* + *smoker* + *infs***Age* + *smoker***Age* + *smoker***infs* | 54863.16 | 0 |
| HV ~ 1 + *smoker* + *smoker***Age* + *smoker***infs* | 54863.24 | 0 |
| HV ~ 1 + *smoker* + *smoker***Age* | 54864.72 | 1 |
| HV ~ 1 + *infs* + *infs***Age* + *smoker***Age* | 54949.12 | 0 |
| HV ~ 1 + *infs* + *infs***Age* | 54949.32 | 1 |
| HV ~ 1 + *infs* + *smoker* + *infs***Age* | 54950.76 | 0 |
| HV ~ 1 + *infs* + *infs***Age* + *smoker***Age* + *smoker***infs* | 54950.98 | 0 |
| HV ~ 1 + *infs* + *infs***Age* + *smoker***infs* | 54951.31 | 0 |
| HV ~ 1 + *infs* + *smoker* + *infs***Age* + *smoker***infs* | 54952.73 | 0 |
| HV ~ 1 + *infs***Age* + *smoker***Age* | 54954.59 | 0 |
| HV ~ 1 + *infs***Age* | 54954.75 | 1 |
| HV ~ 1 + *infs* + *smoker***Age* | 54955.42 | 0 |
| HV ~ 1 + *infs* | 54955.60 | 1 |
| HV ~ 1 + *infs***Age* + *smoker***Age* + *smoker***infs* | 54955.97 | 0 |
| HV ~ 1 + *smoker* + *infs***Age* | 54956.28 | 0 |
| HV ~ 1 + *infs***Age* + *smoker***infs* | 54956.61 | 0 |
| HV ~ 1 + *infs* + *smoker***Age* + *smoker***infs* | 54957.06 | 0 |
| HV ~ 1 + *infs* + *smoker* | 54957.13 | 0 |
| HV ~ 1 + *infs* + *smoker***infs* | 54957.56 | 0 |
| HV ~ 1 + *smoker* + *infs***Age* + *smoker***infs* | 54957.95 | 0 |
| HV ~ 1 + *smoker***infs* | 54958.81 | 1 |
| HV ~ 1 + *infs* + *smoker* + *smoker***infs* | 54958.98 | 0 |
| HV ~ 1 + *smoker***Age* + *smoker***infs* | 54959.39 | 0 |
| HV ~ 1 + *smoker* + *smoker***infs* | 54960.66 | 0 |
| HV ~ 1 + *smoker***Age* | 54961.00 | 0 |
| HV ~ 1 | 54961.49 | 1 |
| HV ~ 1 + *smoker* | 54962.87 | 0 |
|  |  |  |

**Supplementary Table 15.** Regression models, female, age 65-80. Response variable HV=HV (mm^3^) right, independent variables: *Age* – age at the time attending assessment center during imaging visit, *infs*=1 (with history of infections), *infs*=0 (without history of infections), *smoker*=1 (if the subject was a smoker), *smoker*=0 (if the subject was a non-smoker). Per the regression set corresponding to age groups 65-80 and right HV values, all 64 models (with linear terms and their pairwise interactions) in each regression set were analyzed and presented in ascending order by AIC value. Signf=1 means that all regression coefficients were significant (P-value<0.05) in a specific model, Signf=0 means the opposite. For regression model a short notation used. For instance, HV ~ 1 + *Age* + *infs* + *smoker* + *infs***Age* + *smoker***infs* denotes a standard regression equation HV = Intercept + b_1_**Age* + b_2_**infs* + b_3_**smoker* + b_12_**Age***infs* + b_31_**smoker***infs* where Intercept is a constant called the bias term (or intercept term), b_1_, b_2_, b_3_, b_12_, b_31_ are the regression coefficients corresponding to the *Age*, *infs*, *smoker*, *Age***infs*, *smoker***infs* terms in the regression model. The numbers are rounded to two decimal places.

| Regression Model | AIC | Signf |
| --- | --- | --- |
|  |  |  |
| HV ~ 1 + *Age* + *infs* | 54828.95 | 1 |
| HV ~ 1 + *Age* + *infs***Age* | 54829.06 | 0 |
| HV ~ 1 + *Age* + *infs* + *infs***Age* | 54830.71 | 0 |
| HV ~ 1 + *Age* + *infs* + *smoker***infs* | 54830.82 | 0 |
| HV ~ 1 + *Age* | 54830.85 | 1 |
| HV ~ 1 + *Age* + *infs* + *smoker***Age* | 54830.90 | 0 |
| HV ~ 1 + *Age* + *infs* + *smoker* | 54830.90 | 0 |
| HV ~ 1 + *Age* + *smoker***infs* | 54830.95 | 0 |
| HV ~ 1 + *Age* + *infs***Age* + *smoker***infs* | 54830.96 | 0 |
| HV ~ 1 + *Age* + *infs***Age* + *smoker***Age* | 54831.00 | 0 |
| HV ~ 1 + *Age* + *smoker* + *infs***Age* | 54831.01 | 0 |
| HV ~ 1 + *Age* + *infs* + *infs***Age* + *smoker***infs* | 54832.54 | 0 |
| HV ~ 1 + *Age* + *infs* + *infs***Age* + *smoker***Age* | 54832.66 | 0 |
| HV ~ 1 + *Age* + *infs* + *smoker* + *infs***Age* | 54832.66 | 0 |
| HV ~ 1 + *Age* + *infs* + *smoker***Age* + *smoker***infs* | 54832.70 | 0 |
| HV ~ 1 + *Age* + *infs* + *smoker* + *smoker***infs* | 54832.70 | 0 |
| HV ~ 1 + *Age* + *smoker***Age* | 54832.76 | 0 |
| HV ~ 1 + *Age* + *smoker* | 54832.76 | 0 |
| HV ~ 1 + *Age* + *infs***Age* + *smoker***Age* + *smoker***infs* | 54832.85 | 0 |
| HV ~ 1 + *Age* + *smoker* + *infs***Age* + *smoker***infs* | 54832.85 | 0 |
| HV ~ 1 + *Age* + *infs* + *smoker* + *smoker***Age* | 54832.88 | 0 |
| HV ~ 1 + *Age* + *smoker***Age* + *smoker***infs* | 54832.95 | 0 |
| HV ~ 1 + *Age* + *smoker* + *smoker***infs* | 54832.95 | 0 |
| HV ~ 1 + *Age* + *smoker* + *infs***Age* + *smoker***Age* | 54832.98 | 0 |
| HV ~ 1 + *Age* + *infs* + *infs***Age* + *smoker***Age* + *smoker***infs* | 54834.42 | 0 |
| HV ~ 1 + *Age* + *infs* + *smoker* + *infs***Age* + *smoker***infs* | 54834.43 | 0 |
| HV ~ 1 + *Age* + *infs* + *smoker* + *infs***Age* + *smoker***Age* | 54834.63 | 0 |
| HV ~ 1 + *Age* + *infs* + *smoker* + *smoker***Age* + *smoker***infs* | 54834.67 | 0 |
| HV ~ 1 + *Age* + *smoker* + *smoker***Age* | 54834.74 | 0 |
| HV ~ 1 + *Age* + *smoker* + *infs***Age* + *smoker***Age* + *smoker***infs* | 54834.82 | 0 |
| HV ~ 1 + *Age* + *smoker* + *smoker***Age* + *smoker***infs* | 54834.93 | 0 |
| HV ~ 1 + *Age* + *infs* + *smoker* + *infs***Age* + *smoker***Age* + *smoker***infs* | 54836.39 | 0 |
| HV ~ 1 + *smoker* + *infs***Age* + *smoker***Age* | 54888.40 | 1 |
| HV ~ 1 + *infs* + *smoker* + *smoker***Age* | 54888.54 | 1 |
| HV ~ 1 + *smoker* + *infs***Age* + *smoker***Age* + *smoker***infs* | 54889.76 | 0 |
| HV ~ 1 + *infs* + *smoker* + *smoker***Age* + *smoker***infs* | 54889.94 | 0 |
| HV ~ 1 + *infs* + *smoker* + *infs***Age* + *smoker***Age* | 54890.06 | 0 |
| HV ~ 1 + *smoker* + *smoker***Age* | 54891.29 | 1 |
| HV ~ 1 + *smoker* + *smoker***Age* + *smoker***infs* | 54891.51 | 0 |
| HV ~ 1 + *infs* + *smoker* + *infs***Age* + *smoker***Age* + *smoker***infs* | 54891.54 | 0 |
| HV ~ 1 + *infs* + *infs***Age* | 54962.87 | 1 |
| HV ~ 1 + *infs* + *infs***Age* + *smoker***Age* | 54964.32 | 0 |
| HV ~ 1 + *infs* + *infs***Age* + *smoker***infs* | 54964.71 | 0 |
| HV ~ 1 + *infs* + *smoker* + *infs***Age* | 54964.87 | 0 |
| HV ~ 1 + *infs* + *infs***Age* + *smoker***Age* + *smoker***infs* | 54965.93 | 0 |
| HV ~ 1 + *infs* + *smoker* + *infs***Age* + *smoker***infs* | 54966.68 | 0 |
| HV ~ 1 + *infs***Age* | 54968.14 | 1 |
| HV ~ 1 + *infs* | 54968.83 | 1 |
| HV ~ 1 + *infs***Age* + *smoker***infs* | 54969.50 | 0 |
| HV ~ 1 + *infs***Age* + *smoker***Age* | 54969.61 | 0 |
| HV ~ 1 + *smoker* + *infs***Age* | 54970.14 | 0 |
| HV ~ 1 + *infs* + *smoker***Age* | 54970.29 | 0 |
| HV ~ 1 + *infs* + *smoker***infs* | 54970.44 | 0 |
| HV ~ 1 + *infs***Age* + *smoker***Age* + *smoker***infs* | 54970.54 | 0 |
| HV ~ 1 + *infs* + *smoker* | 54970.83 | 0 |
| HV ~ 1 + *smoker* + *infs***Age* + *smoker***infs* | 54971.44 | 0 |
| HV ~ 1 + *infs* + *smoker***Age* + *smoker***infs* | 54971.56 | 0 |
| HV ~ 1 + *smoker***infs* | 54971.93 | 0 |
| HV ~ 1 | 54972.20 | 1 |
| HV ~ 1 + *infs* + *smoker* + *smoker***infs* | 54972.41 | 0 |
| HV ~ 1 + *smoker***Age* | 54973.52 | 0 |
| HV ~ 1 + *smoker***Age* + *smoker***infs* | 54973.63 | 0 |
| HV ~ 1 + *smoker* + *smoker***infs* | 54973.89 | 0 |
| HV ~ 1 + *smoker* | 54974.19 | 0 |
|  |  |  |

**Supplementary Table 16.** In this table, regression coefficients for two best significant models among all models that are presented in ascending order by AIC value in the Supplementary Table 14 and Supplementary Table 15. The best model for HV (mm3) left, female 65-80 corresponds to the Supplementary Table 14 (number one in the list). The best model for HV (mm3) right, female 65-80 corresponds to the Supplementary Table 15 (number one in the list). Notice that both best models do not include variable smoker (though there are many models including variable smoker in the lists). It shows that including variable smoker would not improve the quality of the model with regard to AIC, which rewards goodness-of-fit and model simplicity. Scientific notation ‘e’ means that the base number is multiplied by 10 raised to the given power. The numbers are rounded to two decimal places.

| Model/Term | Estimate | Std. Error | P-value |
| --- | --- | --- | --- |
|  |  |  |  |
| **Best model for HV** **(mm^3^) left, female 65-80** |  |  |  |
| Intercept | 7278.30 (mm^3^) | 184.81 | < 1.00e-50 |
| *Age* | -33.91 (mm^3^/year) | 2.64 | 5.66e-37 |
| *infs* | -86.42 (mm^3^) | 35.13 | 1.39e-02 |
|  |  |  |  |
| **Best model for HV (mm^3^) right, female 65-80** |  |  |  |
| Intercept | 7274.61 (mm^3^) | 185.67 | < 1.00e-50 |
| *Age* | -31.88 (mm^3^/year) | 2.65 | 1.11e-32 |
| *infs* | -69.69 (mm^3^) | 35.29 | 4.84e-02 |
|  |  |  |  |

**Supplementary Table 17.** Numbers of subjects in the UK Biobank sample used in this study for four infection types: acute infections, Flue and Pneumonia, Herpesviral infections, Mycoses for females and males for two age intervals 60-75 and 65-80.

| Group/Subjects |  | Females, age 60-75 | Males, age 60-75 | Females, age 65-80 | Males, age 65-80 |
| --- | --- | --- | --- | --- | --- |
|  |  |  |  |  |  |
| **Acute infections** |  |  |  |  |  |
| History of infection |  | 188 | 134 | 139 | 107 |
| No history of infection |  | 5285 | 4974 | 4009 | 4462 |
| All |  | 5473 | 5108 | 4148 | 4569 |
|  |  |  |  |  |  |
| **Flu and Pneumonia** |  |  |  |  |  |
| History of infection |  | 55 | 53 | 50 | 45 |
| No history of infection |  | 5285 | 4974 | 4009 | 4462 |
| All |  | 5340 | 5027 | 4059 | 4507 |
|  |  |  |  |  |  |
| **Herpesviral infections** |  |  |  |  |  |
| History of infection |  | 10 | 4 | 8 | 4 |
| No history of infection |  | 5285 | 4974 | 4009 | 4462 |
| All |  | 5295 | 4978 | 417 | 4466 |
|  |  |  |  |  |  |
| **Mycoses** |  |  |  |  |  |
| History of infection |  | 22 | 20 | 18 | 19 |
| No history of infection |  | 5285 | 4974 | 4009 | 4462 |
| All |  | 5307 | 4994 | 4027 | 4481 |
|  |  |  |  |  |  |

**Supplementary Table 18**. Numbers of subjects in the UK Biobank sample used in this study for four infection types: acute infections, Flue and Pneumonia, Herpesviral infections, Mycoses for females and males for two age intervals 60-75 and 65-80. Infs and noInfs correspond to the groups of subjects with history of infection and without history of infection; APOE4 and noAPOE4 correspond to the groups of carriers of APOE e4 allele and non-carriers of APOE e4 allele.

| Group/Subjects |  | Females, age 60-75 | Males, age 60-75 | Females, age 65-80 | Males, age 65-80 |
| --- | --- | --- | --- | --- | --- |
|  |  |  |  |  |  |
| **Infections** |  |  |  |  |  |
| Infs_APOE4 |  | 101 | 61 | 79 | 46 |
| Infs_noAPOE4 |  | 252 | 220 | 203 | 190 |
| noInfs_APOE4 |  | 1236 | 1112 | 924 | 989 |
| noInfs_noAPOE4 |  | 3165 | 3003 | 2430 | 2715 |
| All |  | 4754 | 4396 | 3636 | 3940 |
|  |  |  |  |  |  |
| **Acute infections** |  |  |  |  |  |
| Infs_APOE4 |  | 46 | 22 | 30 | 12 |
| Infs_noAPOE4 |  | 118 | 95 | 91 | 81 |
| noInfs_APOE4 |  | 1236 | 1112 | 924 | 989 |
| noInfs_noAPOE4 |  | 3165 | 3003 | 2430 | 2715 |
| All |  | 4565 | 4232 | 3475 | 3797 |
|  |  |  |  |  |  |
| **Flue and Pneumonia** |  |  |  |  |  |
| Infs_APOE4 |  | 15 | 17 | 12 | 14 |
| Infs_noAPOE4 |  | 29 | 29 | 29 | 25 |
| noInfs_APOE4 |  | 1236 | 1112 | 924 | 989 |
| noInfs_noAPOE4 |  | 3165 | 3003 | 2430 | 2715 |
| All |  | 4445 | 4161 | 3395 | 3743 |
|  |  |  |  |  |  |
| **Herpesviral infections** |  |  |  |  |  |
| Infs_APOE4 |  | 2 | 0 | 1 | 1 |
| Infs_noAPOE4 |  | 7 | 4 | 6 | 3 |
| noInfs_APOE4 |  | 1236 | 1112 | 924 | 989 |
| noInfs_noAPOE4 |  | 3165 | 3003 | 2430 | 2715 |
| All |  | 4410 | 4119 | 3361 | 3708 |
|  |  |  |  |  |  |
| **Mycoses** |  |  |  |  |  |
| Infs_APOE4 |  | 2 | 4 | 2 | 3 |
| Infs_noAPOE4 |  | 15 | 14 | 11 | 15 |
| noInfs_APOE4 |  | 1236 | 1112 | 924 | 989 |
| noInfs_noAPOE4 |  | 3165 | 3003 | 2430 | 2715 |
| All |  | 4418 | 4133 | 3367 | 3722 |
|  |  |  |  |  |  |

**Note:** Comparisons are considered between the following groups: Infs_APOE4 (subjects who are both Infs and APOE4) and Infs_noAPOE4 (subjects who are in Infs and are not in APOE4), Infs_APOE4 and noInfs_APOE4 (subjects who are not in Infs and are in APOE4),  Infs_APOE4 and noInfs_noAPOE4 (subjects who are not in Infs and are not in APOE4), Infs_noAPOE4 and noInfs_APOE4, Infs_noAPOE4 and noInfs_noAPOE4, noInfs_APOE4 and noInfs_noAPOE4.

**Supplementary Table 19**. Comparison of HV between women and men with, and without, history of acute infections. The 'effect size' equals to the difference between the left/right HV mean value in group Infs and group noInfs.

| Test | P-value | 95% Confidence Intervals | HV Estimate (mm^3^) |
| --- | --- | --- | --- |
|  |  |  |  |
| **Females, age 60-75, HV (mm^3^) left** |  |  |  |
| ANOVA | 1.15e-01 |  |  |
| HV, *Infs* |  | [4834,5014] | 4918 |
| HV, *noInfs* |  | [4972,5002] | 4986 |
| effect size |  | [-154,17] | -69 |
| **Females, age 65-80, HV (mm^3^) left** |  |  |  |
| ANOVA | 3.68e-02 |  |  |
| HV, *Infs* |  | [4679,4891] | 4791 |
| HV, *noInfs* |  | [4879,4916] | 4897 |
| effect size |  | [-206,-7] | -106 |
| **Females, age 60-75, HV (mm^3^) right** |  |  |  |
| ANOVA | 1.11e-01 |  |  |
| HV, *Infs* |  | [4974,5137] | 5059 |
| HV, *noInfs* |  | [5111,5143] | 5128 |
| effect size |  | [-154,16] | -69 |
| **Females, age 65-80, HV (mm^3^) right** |  |  |  |
| ANOVA | 1.71e-01 |  |  |
| HV, *Infs* |  | [4890,5052] | 4972 |
| HV, *noInfs* |  | [5023,5059] | 5041 |
| effect size |  | [-167,30] | -69 |
| **Males, age 60-75, HV (mm^3^) left** |  |  |  |
| ANOVA | 2.94e-01 |  |  |
| HV, *Infs* |  | [4505,4707] | 4608 |
| HV, *noInfs* |  | [4647,4681] | 4664 |
| effect size |  | [-160,48] | -56 |
| **Males, age 65-80, HV (mm^3^) left** |  |  |  |
| ANOVA | 9.10e-01 |  |  |
| HV, *Infs* |  | [4429,4637] | 4539 |
| HV, *noInfs* |  | [4528,4564] | 4546 |
| effect size |  | [-124,110] | -7 |
| **Males, age 60-75, HV (mm^3^) right** |  |  |  |
| ANOVA | 6.64e-01 |  |  |
| HV, *Infs* |  | [4698,4904] | 4798 |
| HV, *noInfs* |  | [4804,4839] | 4822 |
| effect size |  | [-131,83] | -24 |
| **Males, age 65-80, HV (mm^3^) right** |  |  |  |
| ANOVA | 9.95e-01 |  |  |
| HV, *Infs* |  | [4592,4807] | 4703 |
| HV, *noInfs* |  | [4684,4721] | 4703 |
| effect size |  | [-121,122] | 0 |
|  |  |  |  |

**Supplementary Table 20.** *Age* distributions at the time of collecting acute infectious disease data between January 1, 2009 to October 15, 2014 for those attended assessment center during imaging visit between January 15, 2015 and October 31, 2019 at age 60-75 and 65-80. The numbers are rounded to two decimal places.

| Group | min | max | mean | sd | number |
| --- | --- | --- | --- | --- | --- |
| Female, age 60-75 |  |  |  |  |  |
| *Infs* | 60.08 | 74.77 | 67.29 | 4.09 | 188 |
| no*Infs* | 60.00 | 74.99 | 67.18 | 4.11 | 5285 |
| Male, age 60-75 |  |  |  |  |  |
| *Infs* | 60.15 | 74.73 | 67.58 | 4.06 | 134 |
| no*Infs* | 60.00 | 75.00 | 68.02 | 4.04 | 4974 |
| Female, age 65-80 |  |  |  |  |  |
| *Infs* | 65.13 | 79.75 | 70.92 | 3.51 | 139 |
| no*Infs* | 65.01 | 80.00 | 70.56 | 3.60 | 4009 |
| Male, age 65-80 |  |  |  |  |  |
| *Infs* | 65.05 | 79.30 | 70.85 | 3.66 | 107 |
| no*Infs* | 65.01 | 80.00 | 71.20 | 3.66 | 4462 |

**Supplementary Table 21.** Welch Two Sample t-test for two age distributions, with acute infection (*Infs*) and without acute infection (no*Infs*), at the time of collecting infectious disease data between January 1, 2009 to October 15, 2014 for those attended assessment center during imaging visit between January 15, 2015 and October 31, 2019 at age 60-75 and 65-80. The numbers are rounded to two decimal places.

| Test | P-value | 95% Confidence interval | Estimate | stderr |
| --- | --- | --- | --- | --- |
|  |  |  |  |  |
| Female, age 60-75 | 0.73 | [-0.49,0.70] | 67.29 : 67.18 | 0.30 |
| Male, age 60-75 | 0.22 | [-1.14,0.26] | 67.58 : 68.02 | 0.36 |
| Female, age 65-80 | 0.24 | [-0.24,0.96] | 70.92 : 70.56 | 0.30 |
| Male, age 65-80 | 0.33 | [-1.06,0.36] | 70.85 : 71.20 | 0.36 |
|  |  |  |  |  |

**Supplementary Table 22.** Comparison of HV between women and men with, and without, history of Influenza and Pneumonia.The 'effect size' equals to the difference between the left/right HV mean value in group Infs and group noInfs.

| Test | P-value | 95% Confidence Intervals | HV Estimate (mm^3^) |
| --- | --- | --- | --- |
|  |  |  |  |
| **Females, age 60-75, HV (mm^3^) left** |  |  |  |
| ANOVA | 2.36e-01 |  |  |
| HV, *Infs* |  | [4955,5194] | 5080 |
| HV, *noInfs* |  | [4970,5002] | 4986 |
| effect size |  | [-61,249] | 94 |
| **Females, age 65-80, HV (mm^3^) left** |  |  |  |
| ANOVA | 0.80e-01 |  |  |
| HV, *Infs* |  | [4779,5051] | 4918 |
| HV, *noInfs* |  | [4879,4915] | 4897 |
| effect size |  | [-143,184] | 21 |
| **Females, age 60-75, HV (mm^3^) right** |  |  |  |
| ANOVA | 4.57e-01 |  |  |
| HV, *Infs* |  | [4893,5242] | 5069 |
| HV, *noInfs* |  | [5112,5144] | 5128 |
| effect size |  | [-214,96] | -59 |
| **Females, age 65-80, HV (mm^3^) right** |  |  |  |
| ANOVA | 2.25e-01 |  |  |
| HV, *Infs* |  | [4744,5155] | 4940 |
| HV, *noInfs* |  | [5023,5059] | 5041 |
| effect size |  | [-264,62] | -101 |
| **Males, age 60-75, HV (mm^3^) left** |  |  |  |
| ANOVA | 6.21e-01 |  |  |
| HV, *Infs* |  | [4479,4769] | 4622 |
| HV, *noInfs* |  | [4647,4681] | 4664 |
| effect size |  | [-206,123] | -41 |
| **Males, age 65-80, HV (mm^3^) left** |  |  |  |
| ANOVA | 9.92e-01 |  |  |
| HV, *Infs* |  | [4367,4722] | 4545 |
| HV, *noInfs* |  | [4528,4564] | 4546 |
| effect size |  | [-180,179] | -1 |
| **Males, age 60-75, HV (mm^3^) right** |  |  |  |
| ANOVA | 1.20e-01 |  |  |
| HV, *Infs* |  | [4820,5087] | 4956 |
| HV, *noInfs* |  | [4803,4840] | 4822 |
| effect size |  | [-35,303] | 134 |
| **Males, age 65-80, HV (mm^3^) right** |  |  |  |
| ANOVA | 1.27e-01 |  |  |
| HV, *Infs* |  | [4684,5011] | 4847 |
| HV, *noInfs* |  | [4684,4721] | 4703 |
| effect size |  | [-41,331] | 145 |
|  |  |  |  |

**Supplementary Table 23.** Comparison of the left HV(mm3) between females/males aged 60-75 between groups, all infections (see Note).

| Test | P-value | 95% Confidence Intervals | HV Estimate (mm^3^) |
| --- | --- | --- | --- |
| **Females, age [60-75], HV (mm^3^) left** |  |  |  |
| Tukey | 9.96e-01 |  |  |
| Infs_APOE4 |  | [4836,5043] | 4940 |
| Infs_noAPOE4 |  | [4853,5001] | 4924 |
| Infs_APOE4 - Infs_noAPOE4 |  | [-161,193] | 16 |
| **Females, age [60-75], HV (mm^3^) left** |  |  |  |
| Tukey | 9.92e-01 |  |  |
| Infs_APOE4 |  | [4836,5043] | 4940 |
| noInfs_APOE4 |  | [4928,4989] | 4957 |
| Infs_APOE4 - noInfs_APOE4 |  | [-173,138] | -18 |
| **Females, age [60-75], HV (mm^3^) left** |  |  |  |
| Tukey | 8.02e-01 |  |  |
| Infs_APOE4 |  | [4836,5043] | 4940 |
| noInfs_noAPOE4 |  | [4972,5014] | 4993 |
| Infs_APOE4 - noInfs_noAPOE4 |  | [-206,99] | -54 |
| **Females, age [60-75], HV (mm^3^) left** |  |  |  |
| Tukey | 8.43e-01 |  |  |
| Infs_noAPOE4 |  | [4853,5001] | 4924 |
| noInfs_APOE4 |  | [4928,4989] | 4957 |
| Infs_noAPOE4 - noInfs_APOE4 |  | [-137,71] | -33 |
| F**emales, age [60-75], HV (mm^3^) left** |  |  |  |
| Tukey | 2.69e-01 |  |  |
| Infs_noAPOE4 |  | [4853,5001] | 4924 |
| noInfs_noAPOE4 |  | [4972,5014] | 4993 |
| Infs_noAPOE4 - noInfs_noAPOE4 |  | [-168,29] | -69 |
| **Females, age [60-75], HV (mm^3^) left** |  |  |  |
| Tukey | 2.57e-01 |  |  |
| noInfs_APOE4 |  | [4928,4989] | 4957 |
| noInfs_noAPOE4 |  | [4972,5014] | 4993 |
| noInfs_APOE4 - noInfs_noAPOE4 |  | [-87,14] | -36 |
| **Males, age [60-75], HV (mm^3^) left** |  |  |  |
| Tukey | 9.51e-01 |  |  |
| Infs_APOE4 |  | [4439,4749] | 4597 |
| Infs_noAPOE4 |  | [4558,4718] | 4644 |
| Infs_APOE4 - Infs_noAPOE4 |  | [-273,179] | -47 |
| **Males, age [60-75], HV (mm^3^) left** |  |  |  |
| Tukey | 9.45e-01 |  |  |
| Infs_APOE4 |  | [4439,4749] | 4597 |
| noInfs_APOE4 |  | [4604,4678] | 4642 |
| Infs_APOE4 - noInfs_APOE4 |  | [-250,161] | -44 |
| **Males, age [60-75], HV (mm^3^) left** |  |  |  |
| Tukey | 7.75e-01 |  |  |
| Infs_APOE4 |  | [4439,4749] | 4597 |
| noInfs_noAPOE4 |  | [4650,4694] | 4672 |
| Infs_APOE4 - noInfs_noAPOE4 |  | [-277,127] | -75 |
| **Males, age [60-75], HV (mm^3^) left** |  |  |  |
| Tukey | 1.00e+00 |  |  |
| Infs_noAPOE4 |  | [4558,4718] | 4644 |
| noInfs_APOE4 |  | [4604,4678] | 4642 |
| Infs_noAPOE4 - noInfs_APOE4 |  | [-113,118] | 2 |
| **Males, age [60-75], HV (mm^3^) left** |  |  |  |
| Tukey | 9.10e-01 |  |  |
| Infs_noAPOE4 |  | [4558,4718] | 4644 |
| noInfs_noAPOE4 |  | [4650,4694] | 4672 |
| Infs_noAPOE4 - noInfs_noAPOE4 |  | [-137,81] | -28 |
| **Males, age [60-75], HV (mm^3^) left** |  |  |  |
| Tukey | 4.75e-01 |  |  |
| noInfs_APOE4 |  | [4604,4678] | 4642 |
| noInfs_noAPOE4 |  | [4650,4694] | 4672 |
| noInfs_APOE4 - noInfs_noAPOE4 |  | [-85,24] | -31 |
|  |  |  |  |

**Note:** Infs and noInfs correspond to the groups of subjects with history of infection and without history of infection; APOE4 and noAPOE4 correspond to the groups of carriers of APOE e4 allele and non-carriers of APOE e4 allele. Comparisons are considered between the following groups: Infs_APOE4 (subjects who are both Infs and APOE4) and Infs_noAPOE4 (subjects who are in Infs and are not in APOE4), Infs_APOE4 and noInfs_APOE4 (subjects who are not in Infs and are in APOE4), Infs_APOE4 and noInfs_noAPOE4 (subjects who are not in Infs and are not in APOE4), Infs_noAPOE4 and noInfs_APOE4, Infs_noAPOE4 and noInfs_noAPOE4, noInfs_APOE4 and noInfs_noAPOE4. In this table, the sign minus between two groups denotes the difference between the means in two groups (effect size). For instance, Infs_APOE4 - Infs_noAPOE4 equals to the difference between the left/right HV mean value in the group Infs_APOE4 and group Infs_noAPOE4. Scientific notation ‘e’ means that the base number is multiplied by 10 raised to the given power.

**Supplementary Table 24.** Comparison of the right HV(mm3) between females/males aged 60-75 between groups, all infections (see Note).

| Test | P-value | 95% Confidence Intervals | HV Estimate (mm^3^) |
| --- | --- | --- | --- |
| **Females, age [60-75], HV (mm^3^) right** |  |  |  |
| Tukey | 9.27e-01 |  |  |
| Infs_APOE4 |  | [4913,5175] | 5041 |
| Infs_noAPOE4 |  | [5021,5150] | 5083 |
| Infs_APOE4 - Infs_noAPOE4 |  | [-217,133] | -42 |
| **Females, age [60-75], HV (mm^3^) right** |  |  |  |
| Tukey | 6.37e-01 |  |  |
| Infs_APOE4 |  | [4913,5175] | 5041 |
| noInfs_APOE4 |  | [5081,5144] | 5112 |
| Infs_APOE4 - noInfs_APOE4 |  | [-225,83] | -71 |
| **Females, age [60-75], HV (mm^3^) right** |  |  |  |
| Tukey | 4.20e-01 |  |  |
| Infs_APOE4 |  | [4913,5175] | 5041 |
| noInfs_noAPOE4 |  | [5110,5152] | 5130 |
| Infs_APOE4 - noInfs_noAPOE4 |  | [-240,61] | -90 |
| **Females, age [60-75], HV (mm^3^) right** |  |  |  |
| Tukey | 8.88e-01 |  |  |
| Infs_noAPOE4 |  | [5021,5150] | 5083 |
| noInfs_APOE4 |  | [5081,5144] | 5112 |
| Infs_noAPOE4 - noInfs_APOE4 |  | [-132,74] | -29 |
| F**emales, age [60-75], HV (mm^3^) right** |  |  |  |
| Tukey | 5.94e-01 |  |  |
| Infs_noAPOE4 |  | [5021,5150] | 5083 |
| noInfs_noAPOE4 |  | [5110,5152] | 5130 |
| Infs_noAPOE4 - noInfs_noAPOE4 |  | [-145,50] | -47 |
| **Females, age [60-75], HV (mm^3^) right** |  |  |  |
| Tukey | 7.76e-01 |  |  |
| noInfs_APOE4 |  | [5081,5144] | 5112 |
| noInfs_noAPOE4 |  | [5110,5152] | 5130 |
| noInfs_APOE4 - noInfs_noAPOE4 |  | [-68,31] | -19 |
| **Males, age [60-75], HV (mm^3^) right** |  |  |  |
| Tukey | 5.83e-01 |  |  |
| Infs_APOE4 |  | [4801,5072] | 4936 |
| Infs_noAPOE4 |  | [4744,4907] | 4821 |
| Infs_APOE4 - Infs_noAPOE4 |  | [-118,349] | 115 |
| **Males, age [60-75], HV (mm^3^) right** |  |  |  |
| Tukey | 2.38e-01 |  |  |
| Infs_APOE4 |  | [4801,5072] | 4936 |
| noInfs_APOE4 |  | [4743,4821] | 4781 |
| Infs_APOE4 - noInfs_APOE4 |  | [-57,367] | 155 |
| **Males, age [60-75], HV (mm^3^) right** |  |  |  |
| Tukey | 5.54e-01 |  |  |
| Infs_APOE4 |  | [4801,5072] | 4936 |
| noInfs_noAPOE4 |  | [4807,4852] | 4830 |
| Infs_APOE4 - noInfs_noAPOE4 |  | [-102,315] | 107 |
| **Males, age [60-75], HV (mm^3^) right** |  |  |  |
| Tukey | 8.28e-01 |  |  |
| Infs_noAPOE4 |  | [4744,4907] | 4821 |
| noInfs_APOE4 |  | [4743,4821] | 4781 |
| Infs_noAPOE4 - noInfs_APOE4 |  | [-79,159] | 40 |
| **Males, age [60-75], HV (mm^3^) right** |  |  |  |
| Tukey | 9.97e-01 |  |  |
| Infs_noAPOE4 |  | [4744,4907] | 4821 |
| noInfs_noAPOE4 |  | [4807,4852] | 4830 |
| Infs_noAPOE4 - noInfs_noAPOE4 |  | [-121,104] | -9 |
| **Males, age [60-75], HV (mm3) right** |  |  |  |
| Tukey | 1.26e-01 |  |  |
| noInfs_APOE4 |  | [4743,4821] | 4781 |
| noInfs_noAPOE4 |  | [4807,4852] | 4830 |
| noInfs_APOE4 - noInfs_noAPOE4 |  | [-105,8] | -48 |
|  |  |  |  |

**Note:** Infs and noInfs correspond to the groups of subjects with history of infection and without history of infection; APOE4 and noAPOE4 correspond to the groups of carriers of APOE e4 allele and non-carriers of APOE e4 allele. Comparisons are considered between the following groups: Infs_APOE4 (subjects who are both Infs and APOE4) and Infs_noAPOE4 (subjects who are in Infs and are not in APOE4), Infs_APOE4 and noInfs_APOE4 (subjects who are not in Infs and are in APOE4), Infs_APOE4 and noInfs_noAPOE4 (subjects who are not in Infs and are not in APOE4), Infs_noAPOE4 and noInfs_APOE4, Infs_noAPOE4 and noInfs_noAPOE4, noInfs_APOE4 and noInfs_noAPOE4. In this table, the sign minus between two groups denotes the difference between the means in two groups (effect size). For instance, Infs_APOE4 - Infs_noAPOE4 equals to the difference between the left/right HV mean value in the group Infs_APOE4 and group Infs_noAPOE4. Scientific notation ‘e’ means that the base number is multiplied by 10 raised to the given power.

**Supplementary Table 25.** Comparison of the left HV(mm3) between females/males aged 65-80 between groups, all infections (see Note).

| Test | P-value | 95% Confidence Intervals | HV Estimate (mm^3^) |
| --- | --- | --- | --- |
| **Females, age [65-80], HV (mm^3^) left** |  |  |  |
| Tukey | 6.77e-01 |  |  |
| Infs_APOE4 |  | [4774,5008] | 4890 |
| Infs_noAPOE4 |  | [4719,4885] | 4803 |
| Infs_APOE4 - Infs_noAPOE4 |  | [-112,286] | 87 |
| **Females, age [65-80], HV (mm^3^) left** |  |  |  |
| Tukey | 9.79e-01 |  |  |
| Infs_APOE4 |  | [4774,5008] | 4890 |
| noInfs_APOE4 |  | [4825,4901] | 4863 |
| Infs_APOE4 - noInfs_APOE4 |  | [-149,204] | 27 |
| **Females, age [65-80], HV (mm^3^) left** |  |  |  |
| Tukey | 9.92e-01 |  |  |
| Infs_APOE4 |  | [4774,5008] | 4890 |
| noInfs_noAPOE4 |  | [4885,4934] | 4909 |
| Infs_APOE4 - noInfs_noAPOE4 |  | [-191,153] | -19 |
| **Females, age [65-80], HV (mm^3^) left** |  |  |  |
| Tukey | 5.54e-01 |  |  |
| Infs_noAPOE4 |  | [4719,4885] | 4803 |
| noInfs_APOE4 |  | [4825,4901] | 4863 |
| Infs_noAPOE4 - noInfs_APOE4 |  | [-176,57] | -60 |
| F**emales, age [65-80], HV (mm^3^) left** |  |  |  |
| Tukey | 6.25e-02 |  |  |
| Infs_noAPOE4 |  | [4719,4885] | 4803 |
| noInfs_noAPOE4 |  | [4885,4934] | 4909 |
| Infs_noAPOE4 - noInfs_noAPOE4 |  | [-216,4] | -106 |
| **Females, age [65-80], HV (mm^3^) left** |  |  |  |
| Tukey | 1.66e-01 |  |  |
| noInfs_APOE4 |  | [4825,4901] | 4863 |
| noInfs_noAPOE4 |  | [4885,4934] | 4909 |
| noInfs_APOE4 - noInfs_noAPOE4 |  | [-105,12] | -47 |
| **Males, age [65-80], HV (mm^3^) left** |  |  |  |
| Tukey | 9.63e-01 |  |  |
| Infs_APOE4 |  | [4329,4660] | 4495 |
| Infs_noAPOE4 |  | [4461,4626] | 4543 |
| Infs_APOE4 - Infs_noAPOE4 |  | [-304,208] | -48 |
| **Males, age [65-80], HV (mm^3^) left** |  |  |  |
| Tukey | 9.89e-01 |  |  |
| Infs_APOE4 |  | [4329,4660] | 4495 |
| noInfs_APOE4 |  | [4485,4563] | 4524 |
| Infs_APOE4 - noInfs_APOE4 |  | [-263,206] | -29 |
| **Males, age [65-80], HV (mm^3^) left** |  |  |  |
| Tukey | 9.02e-01 |  |  |
| Infs_APOE4 |  | [4329,4660] | 4495 |
| noInfs_noAPOE4 |  | [4534,4581] | 4557 |
| Infs_APOE4 - noInfs_noAPOE4 |  | [-293,170] | -62 |
| **Males, age [65-80], HV (mm^3^) left** |  |  |  |
| Tukey | 9.78e-01 |  |  |
| Infs_noAPOE4 |  | [4461,4626] | 4543 |
| noInfs_APOE4 |  | [4485,4563] | 4524 |
| Infs_noAPOE4 - noInfs_APOE4 |  | [-104,143] | 19 |
| **Males, age [65-80], HV (mm^3^) left** |  |  |  |
| Tukey | 9.90e-01 |  |  |
| Infs_noAPOE4 |  | [4461,4626] | 4543 |
| noInfs_noAPOE4 |  | [4534,4581] | 4557 |
| Infs_noAPOE4 - noInfs_noAPOE4 |  | [-131,103] | -14 |
| **Males, age [65-80], HV (mm^3^) left** |  |  |  |
| Tukey | 4.47e-01 |  |  |
| noInfs_APOE4 |  | [4485,4563] | 4524 |
| noInfs_noAPOE4 |  | [4534,4581] | 4557 |
| noInfs_APOE4 - noInfs_noAPOE4 |  | [-91,24] | -33 |
|  |  |  |  |

**Note:** Infs and noInfs correspond to the groups of subjects with history of infection and without history of infection; APOE4 and noAPOE4 correspond to the groups of carriers of APOE e4 allele and non-carriers of APOE e4 allele. Comparisons are considered between the following groups: Infs_APOE4 (subjects who are both Infs and APOE4) and Infs_noAPOE4 (subjects who are in Infs and are not in APOE4), Infs_APOE4 and noInfs_APOE4 (subjects who are not in Infs and are in APOE4), Infs_APOE4 and noInfs_noAPOE4 (subjects who are not in Infs and are not in APOE4), Infs_noAPOE4 and noInfs_APOE4, Infs_noAPOE4 and noInfs_noAPOE4, noInfs_APOE4 and noInfs_noAPOE4. In this table, the sign minus between two groups denotes the difference between the means in two groups (effect size). For instance, Infs_APOE4 - Infs_noAPOE4 equals to the difference between the left/right HV mean value in the group Infs_APOE4 and group Infs_noAPOE4. Scientific notation ‘e’ means that the base number is multiplied by 10 raised to the given power.

**Supplementary Table 26.** Comparison of the right HV(mm3) between females/males aged 65-80 between groups, all infections (see Note).

| Test | P-value | 95% Confidence Intervals | HV Estimate (mm^3^) |
| --- | --- | --- | --- |
| **Females, age [65-80], HV (mm^3^) right** |  |  |  |
| Tukey | 9.74e-01 |  |  |
| Infs_APOE4 |  | [4875,5130] | 4996 |
| Infs_noAPOE4 |  | [4889,5040] | 4963 |
| Infs_APOE4 - Infs_noAPOE4 |  | [-165,230] | 33 |
| **Females, age [65-80], HV (mm^3^) right** |  |  |  |
| Tukey | 9.92e-01 |  |  |
| Infs_APOE4 |  | [4875,5130] | 4996 |
| noInfs_APOE4 |  | [4978,5051] | 5015 |
| Infs_APOE4 - noInfs_APOE4 |  | [-194,155] | -19 |
| **Females, age [65-80], HV (mm^3^) right** |  |  |  |
| Tukey | 8.48e-01 |  |  |
| Infs_APOE4 |  | [4875,5130] | 4996 |
| noInfs_noAPOE4 |  | [5026,5072] | 5050 |
| Infs_APOE4 - noInfs_noAPOE4 |  | [-224,116] | -54 |
| **Females, age [65-80], HV (mm^3^) right** |  |  |  |
| Tukey | 6.55e-01 |  |  |
| Infs_noAPOE4 |  | [4889,5040] | 4963 |
| noInfs_APOE4 |  | [4978,5051] | 5015 |
| Infs_noAPOE4 - noInfs_APOE4 |  | [-167,64] | -52 |
| F**emales, age [65-80], HV (mm^3^) right** |  |  |  |
| Tukey | 1.72e-01 |  |  |
| Infs_noAPOE4 |  | [4889,5040] | 4963 |
| noInfs_noAPOE4 |  | [5026,5072] | 5050 |
| Infs_noAPOE4 - noInfs_noAPOE4 |  | [-195,22] | -87 |
| **Females, age [65-80], HV (mm^3^) right** |  |  |  |
| Tukey | 4.11e-01 |  |  |
| noInfs_APOE4 |  | [4978,5051] | 5015 |
| noInfs_noAPOE4 |  | [5026,5072] | 5050 |
| noInfs_APOE4 - noInfs_noAPOE4 |  | [-92,23] | -35 |
| **Males, age [65-80], HV (mm^3^) right** |  |  |  |
| Tukey | 9.94e-01 |  |  |
| Infs_APOE4 |  | [4538,4870] | 4706 |
| Infs_noAPOE4 |  | [4651,4819] | 4732 |
| Infs_APOE4 - Infs_noAPOE4 |  | [-295,242] | -27 |
| **Males, age [65-80], HV (mm^3^) right** |  |  |  |
| Tukey | 9.84e-01 |  |  |
| Infs_APOE4 |  | [4538,4870] | 4706 |
| noInfs_APOE4 |  | [4632,4712] | 4671 |
| Infs_APOE4 - noInfs_APOE4 |  | [-212,281] | 35 |
| **Males, age [65-80], HV (mm^3^) right** |  |  |  |
| Tukey | 1.00e+00 |  |  |
| Infs_APOE4 |  | [4538,4870] | 4706 |
| noInfs_noAPOE4 |  | [4685,4732] | 4707 |
| Infs_APOE4 - noInfs_noAPOE4 |  | [-244,242] | -1 |
| **Males, age [65-80], HV (mm^3^) right** |  |  |  |
| Tukey | 6.16e-01 |  |  |
| Infs_noAPOE4 |  | [4651,4819] | 4732 |
| noInfs_APOE4 |  | [4632,4712] | 4671 |
| Infs_noAPOE4 - noInfs_APOE4 |  | [-68,191] | 61 |
| **Males, age [65-80], HV (mm^3^) right** |  |  |  |
| Tukey | 9.51e-01 |  |  |
| Infs_noAPOE4 |  | [4651,4819] | 4732 |
| noInfs_noAPOE4 |  | [4685,4732] | 4707 |
| Infs_noAPOE4 - noInfs_noAPOE4 |  | [-97,148] | 25 |
| **Males, age [60-75], HV (mm3) right** |  |  |  |
| Tukey | 4.25e-01 |  |  |
| noInfs_APOE4 |  | [4632,4712] | 4671 |
| noInfs_noAPOE4 |  | [4685,4732] | 4707 |
| noInfs_APOE4 - noInfs_noAPOE4 |  | [-97,25] | -36 |
|  |  |  |  |

**Note:** Infs and noInfs correspond to the groups of subjects with history of infection and without history of infection; APOE4 and noAPOE4 correspond to the groups of carriers of APOE e4 allele and non-carriers of APOE e4 allele. Comparisons are considered between the following groups: Infs_APOE4 (subjects who are both Infs and APOE4) and Infs_noAPOE4 (subjects who are in Infs and are not in APOE4), Infs_APOE4 and noInfs_APOE4 (subjects who are not in Infs and are in APOE4), Infs_APOE4 and noInfs_noAPOE4 (subjects who are not in Infs and are not in APOE4), Infs_noAPOE4 and noInfs_APOE4, Infs_noAPOE4 and noInfs_noAPOE4, noInfs_APOE4 and noInfs_noAPOE4. In this table, the sign minus between two groups denotes the difference between the means in two groups (effect size). For instance, Infs_APOE4 - Infs_noAPOE4 equals to the difference between the left/right HV mean value in the group Infs_APOE4 and group Infs_noAPOE4. Scientific notation ‘e’ means that the base number is multiplied by 10 raised to the given power.

**Supplementary Table 27.** Comparison of the left HV(mm3) between females/males aged 60-75 between groups, acute infections (see Note).

| Test | P-value | 95% Confidence Intervals | HV Estimate (mm^3^) |
| --- | --- | --- | --- |
| **Females, age [60-75], HV (mm^3^) left** |  |  |  |
| Tukey | 8.41e-01 |  |  |
| Infs_APOE4 |  | [4709,5027] | 4861 |
| Infs_noAPOE4 |  | [4832,5058] | 4946 |
| Infs_APOE4 - Infs_noAPOE4 |  | [-346,177] | -85 |
| **Females, age [60-75], HV (mm^3^) left** |  |  |  |
| Tukey | 6.97e-01 |  |  |
| Infs_APOE4 |  | [4709,5027] | 4861 |
| noInfs_APOE4 |  | [4926,4989] | 4957 |
| Infs_APOE4 - noInfs_APOE4 |  | [-322,130] | -96 |
| **Females, age [60-75], HV (mm^3^) left** |  |  |  |
| Tukey | 4.29e-01 |  |  |
| Infs_APOE4 |  | [4709,5027] | 4861 |
| noInfs_noAPOE4 |  | [4973,5014] | 4993 |
| Infs_APOE4 - noInfs_noAPOE4 |  | [-356,92] | -132 |
| **Females, age [60-75], HV (mm^3^) left** |  |  |  |
| Tukey | 9.97e-01 |  |  |
| Infs_noAPOE4 |  | [4832,5058] | 4946 |
| noInfs_APOE4 |  | [4926,4989] | 4957 |
| Infs_noAPOE4 - noInfs_APOE4 |  | [-156,134] | -11 |
| F**emales, age [60-75], HV (mm^3^) left** |  |  |  |
| Tukey | 8.25e-01 |  |  |
| Infs_noAPOE4 |  | [4832,5058] | 4946 |
| noInfs_noAPOE4 |  | [4973,5014] | 4993 |
| Infs_noAPOE4 - noInfs_noAPOE4 |  | [-189,94] | -47 |
| **Females, age [60-75], HV (mm^3^) left** |  |  |  |
| Tukey | 2.58e-01 |  |  |
| noInfs_APOE4 |  | [4926,4989] | 4957 |
| noInfs_noAPOE4 |  | [4973,5014] | 4993 |
| noInfs_APOE4 - noInfs_noAPOE4 |  | [-87,14] | -36 |
| **Males, age [60-75], HV (mm^3^) left** |  |  |  |
| Tukey | 9.50e-01 |  |  |
| Infs_APOE4 |  | [4489,4918] | 4697 |
| Infs_noAPOE4 |  | [4499,4732] | 4620 |
| Infs_APOE4 - Infs_noAPOE4 |  | [-292,447] | 77 |
| **Males, age [60-75], HV (mm^3^) left** |  |  |  |
| Tukey | 9.75e-01 |  |  |
| Infs_APOE4 |  | [4489,4918] | 4697 |
| noInfs_APOE4 |  | [4605,4677] | 4642 |
| Infs_APOE4 - noInfs_APOE4 |  | [-281,391] | 55 |
| **Males, age [60-75], HV (mm^3^) left** |  |  |  |
| Tukey | 9.98e-01 |  |  |
| Infs_APOE4 |  | [4489,4918] | 4697 |
| noInfs_noAPOE4 |  | [4651,4696] | 4672 |
| Infs_APOE4 - noInfs_noAPOE4 |  | [-310,358] | 24 |
| **Males, age [60-75], HV (mm^3^) left** |  |  |  |
| Tukey | 9.86e-01 |  |  |
| Infs_noAPOE4 |  | [4499,4732] | 4620 |
| noInfs_APOE4 |  | [4605,4677] | 4642 |
| Infs_noAPOE4 - noInfs_APOE4 |  | [-189,145] | -22 |
| **Males, age [60-75], HV (mm^3^) left** |  |  |  |
| Tukey | 8.38e-01 |  |  |
| Infs_noAPOE4 |  | [4499,4732] | 4620 |
| noInfs_noAPOE4 |  | [4651,4696] | 4672 |
| Infs_noAPOE4 - noInfs_noAPOE4 |  | [-216,110] | -53 |
| **Males, age [60-75], HV (mm^3^) left** |  |  |  |
| Tukey | 4.76e-01 |  |  |
| noInfs_APOE4 |  | [4605,4677] | 4642 |
| noInfs_noAPOE4 |  | [4651,4696] | 4672 |
| noInfs_APOE4 - noInfs_noAPOE4 |  | [-85,24] | -31 |
|  |  |  |  |

**Note:** Infs and noInfs correspond to the groups of subjects with history of acute infection and without history of acute infection; APOE4 and noAPOE4 correspond to the groups of carriers of APOE e4 allele and non-carriers of APOE e4 allele. Comparisons are considered between the following groups: Infs_APOE4 (subjects who are both Infs and APOE4) and Infs_noAPOE4 (subjects who are in Infs and are not in APOE4), Infs_APOE4 and noInfs_APOE4 (subjects who are not in Infs and are in APOE4), Infs_APOE4 and noInfs_noAPOE4 (subjects who are not in Infs and are not in APOE4), Infs_noAPOE4 and noInfs_APOE4, Infs_noAPOE4 and noInfs_noAPOE4, noInfs_APOE4 and noInfs_noAPOE4. In this table, the sign minus between two groups denotes the difference between the means in two groups (effect size). For instance, Infs_APOE4 - Infs_noAPOE4 equals to the difference between the left/right HV mean value in the group Infs_APOE4 and group Infs_noAPOE4. Scientific notation ‘e’ means that the base number is multiplied by 10 raised to the given power.

**Supplementary Table 28.** Comparison of the right HV(mm3) between females/males aged 60-75 between groups, acute infections (see Note).

| Test | P-value | 95% Confidence Intervals | HV Estimate (mm^3^) |
| --- | --- | --- | --- |
| **Females, age [60-75], HV (mm^3^) right** |  |  |  |
| Tukey | 8.20e-01 |  |  |
| Infs_APOE4 |  | [4817,5183] | 4999 |
| Infs_noAPOE4 |  | [4995,5180] | 5087 |
| Infs_APOE4 - Infs_noAPOE4 |  | [-347,171] | -88 |
| **Females, age [60-75], HV (mm^3^) right** |  |  |  |
| Tukey | 5.67e-01 |  |  |
| Infs_APOE4 |  | [4817,5183] | 4999 |
| noInfs_APOE4 |  | [5079,5144] | 5112 |
| Infs_APOE4 - noInfs_APOE4 |  | [-336,111] | -113 |
| **Females, age [60-75], HV (mm^3^) right** |  |  |  |
| Tukey | 4.23e-01 |  |  |
| Infs_APOE4 |  | [4817,5183] | 4999 |
| noInfs_noAPOE4 |  | [5110,5151] | 5130 |
| Infs_APOE4 - noInfs_noAPOE4 |  | [-353,90] | -131 |
| **Females, age [60-75], HV (mm^3^) right** |  |  |  |
| Tukey | 9.70e-01 |  |  |
| Infs_noAPOE4 |  | [4995,5180] | 5087 |
| noInfs_APOE4 |  | [5079,5144] | 5112 |
| Infs_noAPOE4 - noInfs_APOE4 |  | [-169,119] | -25 |
| F**emales, age [60-75], HV (mm^3^) right** |  |  |  |
| Tukey | 8.55e-01 |  |  |
| Infs_noAPOE4 |  | [4995,5180] | 5087 |
| noInfs_noAPOE4 |  | [5110,5151] | 5130 |
| Infs_noAPOE4 - noInfs_noAPOE4 |  | [-183,96] | -43 |
| **Females, age [60-75], HV (mm^3^) right** |  |  |  |
| Tukey | 7.76e-01 |  |  |
| noInfs_APOE4 |  | [5079,5144] | 5112 |
| noInfs_noAPOE4 |  | [5110,5151] | 5130 |
| noInfs_APOE4 - noInfs_noAPOE4 |  | [-69,31] | -19 |
| **Males, age [60-75], HV (mm^3^) right** |  |  |  |
| Tukey | 1.00e+00 |  |  |
| Infs_APOE4 |  | [4564,5068] | 4815 |
| Infs_noAPOE4 |  | [4697,4959] | 4829 |
| Infs_APOE4 - Infs_noAPOE4 |  | [-397,368] | -14 |
| **Males, age [60-75], HV (mm^3^) right** |  |  |  |
| Tukey | 9.94e-01 |  |  |
| Infs_APOE4 |  | [4564,5068] | 4815 |
| noInfs_APOE4 |  | [4744,4818] | 4781 |
| Infs_APOE4 - noInfs_APOE4 |  | [-314,382] | 34 |
| **Males, age [60-75], HV (mm^3^) right** |  |  |  |
| Tukey | 1.00e+00 |  |  |
| Infs_APOE4 |  | [4564,5068] | 4815 |
| noInfs_noAPOE4 |  | [4808,4854] | 4830 |
| Infs_APOE4 - noInfs_noAPOE4 |  | [-360,332] | -14 |
| **Males, age [60-75], HV (mm^3^) right** |  |  |  |
| Tukey | 8.91e-01 |  |  |
| Infs_noAPOE4 |  | [4697,4959] | 4829 |
| noInfs_APOE4 |  | [4744,4818] | 4781 |
| Infs_noAPOE4 - noInfs_APOE4 |  | [-125,221] | 48 |
| **Males, age [60-75], HV (mm^3^) right** |  |  |  |
| Tukey | 1.00e+00 |  |  |
| Infs_noAPOE4 |  | [4697,4959] | 4829 |
| noInfs_noAPOE4 |  | [4808,4854] | 4830 |
| Infs_noAPOE4 - noInfs_noAPOE4 |  | [-169,168] | 0 |
| **Males, age [60-75], HV (mm3) right** |  |  |  |
| Tukey | 1.27e-01 |  |  |
| noInfs_APOE4 |  | [4744,4818] | 4781 |
| noInfs_noAPOE4 |  | [4808,4854] | 4830 |
| noInfs_APOE4 - noInfs_noAPOE4 |  | [-105,8] | -48 |
|  |  |  |  |

**Note:** Infs and noInfs correspond to the groups of subjects with history of acute infection and without history of acute infection; APOE4 and noAPOE4 correspond to the groups of carriers of APOE e4 allele and non-carriers of APOE e4 allele. Comparisons are considered between the following groups: Infs_APOE4 (subjects who are both Infs and APOE4) and Infs_noAPOE4 (subjects who are in Infs and are not in APOE4), Infs_APOE4 and noInfs_APOE4 (subjects who are not in Infs and are in APOE4), Infs_APOE4 and noInfs_noAPOE4 (subjects who are not in Infs and are not in APOE4), Infs_noAPOE4 and noInfs_APOE4, Infs_noAPOE4 and noInfs_noAPOE4, noInfs_APOE4 and noInfs_noAPOE4. In this table, the sign minus between two groups denotes the difference between the means in two groups (effect size). For instance, Infs_APOE4 - Infs_noAPOE4 equals to the difference between the left/right HV mean value in the group Infs_APOE4 and group Infs_noAPOE4. Scientific notation ‘e’ means that the base number is multiplied by 10 raised to the given power.

**Supplementary Table 29.** Comparison of the left HV(mm3) between females/males aged 65-80 between groups, acute infections (see Note).

| Test | P-value | 95% Confidence Intervals | HV Estimate (mm^3^) |
| --- | --- | --- | --- |
| **Females, age [65-80], HV (mm^3^) left** |  |  |  |
| Tukey | 9.96e-01 |  |  |
| Infs_APOE4 |  | [4603,5077] | 4832 |
| Infs_noAPOE4 |  | [4676,4931] | 4803 |
| Infs_APOE4 - Infs_noAPOE4 |  | [-288,345] | 29 |
| **Females, age [65-80], HV (mm^3^) left** |  |  |  |
| Tukey | 9.92e-01 |  |  |
| Infs_APOE4 |  | [4603,5077] | 4832 |
| noInfs_APOE4 |  | [4823,4897] | 4863 |
| Infs_APOE4 - noInfs_APOE4 |  | [-310,249] | -30 |
| **Females, age [65-80], HV (mm^3^) left** |  |  |  |
| Tukey | 8.91e-01 |  |  |
| Infs_APOE4 |  | [4603,5077] | 4832 |
| noInfs_noAPOE4 |  | [4885,4929] | 4909 |
| Infs_APOE4 - noInfs_noAPOE4 |  | [-353,199] | -77 |
| **Females, age [65-80], HV (mm^3^) left** |  |  |  |
| Tukey | 7.95e-01 |  |  |
| Infs_noAPOE4 |  | [4676,4931] | 4803 |
| noInfs_APOE4 |  | [4823,4897] | 4863 |
| Infs_noAPOE4 - noInfs_APOE4 |  | [-224,106] | -59 |
| F**emales, age [65-80], HV (mm^3^) left** |  |  |  |
| Tukey | 3.28e-01 |  |  |
| Infs_noAPOE4 |  | [4676,4931] | 4803 |
| noInfs_noAPOE4 |  | [4885,4929] | 4909 |
| Infs_noAPOE4 - noInfs_noAPOE4 |  | [-266,55] | -106 |
| **Females, age [65-80], HV (mm^3^) left** |  |  |  |
| Tukey | 1.67e-01 |  |  |
| noInfs_APOE4 |  | [4823,4897] | 4863 |
| noInfs_noAPOE4 |  | [4885,4929] | 4909 |
| noInfs_APOE4 - noInfs_noAPOE4 |  | [-105,12] | -47 |
| **Males, age [65-80], HV (mm^3^) left** |  |  |  |
| Tukey | 9.94e-01 |  |  |
| Infs_APOE4 |  | [4132,4923] | 4509 |
| Infs_noAPOE4 |  | [4435,4691] | 4558 |
| Infs_APOE4 - Infs_noAPOE4 |  | [-531,434] | -49 |
| **Males, age [65-80], HV (mm^3^) left** |  |  |  |
| Tukey | 1.00e+00 |  |  |
| Infs_APOE4 |  | [4132,4923] | 4509 |
| noInfs_APOE4 |  | [4486,4565] | 4524 |
| Infs_APOE4 - noInfs_APOE4 |  | [-468,439] | -14 |
| **Males, age [65-80], HV (mm^3^) left** |  |  |  |
| Tukey | 9.93e-01 |  |  |
| Infs_APOE4 |  | [4132,4923] | 4509 |
| noInfs_noAPOE4 |  | [4533,4581] | 4557 |
| Infs_APOE4 - noInfs_noAPOE4 |  | [-499,404] | -48 |
| **Males, age [65-80], HV (mm^3^) left** |  |  |  |
| Tukey | 9.62e-01 |  |  |
| Infs_noAPOE4 |  | [4435,4691] | 4558 |
| noInfs_APOE4 |  | [4486,4565] | 4524 |
| Infs_noAPOE4 - noInfs_APOE4 |  | [-146,215] | 34 |
| **Males, age [65-80], HV (mm^3^) left** |  |  |  |
| Tukey | 1.00e+00 |  |  |
| Infs_noAPOE4 |  | [4435,4691] | 4558 |
| noInfs_noAPOE4 |  | [4533,4581] | 4557 |
| Infs_noAPOE4 - noInfs_noAPOE4 |  | [-175,177] | 1 |
| **Males, age [65-80], HV (mm^3^) left** |  |  |  |
| Tukey | 4.50e-01 |  |  |
| noInfs_APOE4 |  | [4486,4565] | 4524 |
| noInfs_noAPOE4 |  | [4533,4581] | 4557 |
| noInfs_APOE4 - noInfs_noAPOE4 |  | [-91,25] | -33 |
|  |  |  |  |

**Note:** Infs and noInfs correspond to the groups of subjects with history of acute infection and without history of acute infection; APOE4 and noAPOE4 correspond to the groups of carriers of APOE e4 allele and non-carriers of APOE e4 allele. Comparisons are considered between the following groups: Infs_APOE4 (subjects who are both Infs and APOE4) and Infs_noAPOE4 (subjects who are in Infs and are not in APOE4), Infs_APOE4 and noInfs_APOE4 (subjects who are not in Infs and are in APOE4), Infs_APOE4 and noInfs_noAPOE4 (subjects who are not in Infs and are not in APOE4), Infs_noAPOE4 and noInfs_APOE4, Infs_noAPOE4 and noInfs_noAPOE4, noInfs_APOE4 and noInfs_noAPOE4. In this table, the sign minus between two groups denotes the difference between the means in two groups (effect size). For instance, Infs_APOE4 - Infs_noAPOE4 equals to the difference between the left/right HV mean value in the group Infs_APOE4 and group Infs_noAPOE4. Scientific notation ‘e’ means that the base number is multiplied by 10 raised to the given power.

**Supplementary Table 30.** Comparison of the right HV(mm3) between females/males aged 65-80 between groups, acute infections (see Note).

| Test | P-value | 95% Confidence Intervals | HV Estimate (mm^3^) |
| --- | --- | --- | --- |
| **Females, age [65-80], HV (mm^3^) right** |  |  |  |
| Tukey | 9.29e-01 |  |  |
| Infs_APOE4 |  | [4837,5247] | 5028 |
| Infs_noAPOE4 |  | [4854,5056] | 4954 |
| Infs_APOE4 - Infs_noAPOE4 |  | [-239,387] | 74 |
| **Females, age [65-80], HV (mm^3^) right** |  |  |  |
| Tukey | 9.99e-01 |  |  |
| Infs_APOE4 |  | [4837,5247] | 5028 |
| noInfs_APOE4 |  | [4977,5050] | 5015 |
| Infs_APOE4 - noInfs_APOE4 |  | [-263,289] | 13 |
| **Females, age [65-80], HV (mm^3^) right** |  |  |  |
| Tukey | 9.97e-01 |  |  |
| Infs_APOE4 |  | [4837,5247] | 5028 |
| noInfs_noAPOE4 |  | [5024,5073] | 5050 |
| Infs_APOE4 - noInfs_noAPOE4 |  | [-295,251] | -22 |
| **Females, age [65-80], HV (mm^3^) right** |  |  |  |
| Tukey | 7.68e-01 |  |  |
| Infs_noAPOE4 |  | [4854,5056] | 4954 |
| noInfs_APOE4 |  | [4977,5050] | 5015 |
| Infs_noAPOE4 - noInfs_APOE4 |  | [-225,102] | -62 |
| F**emales, age [65-80], HV (mm^3^) right** |  |  |  |
| Tukey | 4.04e-01 |  |  |
| Infs_noAPOE4 |  | [4854,5056] | 4954 |
| noInfs_noAPOE4 |  | [5024,5073] | 5050 |
| Infs_noAPOE4 - noInfs_noAPOE4 |  | [-255,63] | -96 |
| **Females, age [65-80], HV (mm^3^) right** |  |  |  |
| Tukey | 4.09e-01 |  |  |
| noInfs_APOE4 |  | [4977,5050] | 5015 |
| noInfs_noAPOE4 |  | [5024,5073] | 5050 |
| noInfs_APOE4 - noInfs_noAPOE4 |  | [-92,23] | -35 |
| **Males, age [65-80], HV (mm^3^) right** |  |  |  |
| Tukey | 9.91E-01 |  |  |
| Infs_APOE4 |  | [4314,5042] | 4668 |
| Infs_noAPOE4 |  | [4601,4873] | 4727 |
| Infs_APOE4 - Infs_noAPOE4 |  | [-566,447] | -59 |
| **Males, age [65-80], HV (mm^3^) right** |  |  |  |
| Tukey | 1.00e+00 |  |  |
| Infs_APOE4 |  | [4314,5042] | 4668 |
| noInfs_APOE4 |  | [4633,4710] | 4671 |
| Infs_APOE4 - noInfs_APOE4 |  | [-478,472] | -3 |
| **Males, age [65-80], HV (mm^3^) right** |  |  |  |
| Tukey | 9.97e-01 |  |  |
| Infs_APOE4 |  | [4314,5042] | 4668 |
| noInfs_noAPOE4 |  | [4684,4731] | 4707 |
| Infs_APOE4 - noInfs_noAPOE4 |  | [-512,434] | -39 |
| **Males, age [65-80], HV (mm^3^) right** |  |  |  |
| Tukey | 8.71e-01 |  |  |
| Infs_noAPOE4 |  | [4601,4873] | 4727 |
| noInfs_APOE4 |  | [4633,4710] | 4671 |
| Infs_noAPOE4 - noInfs_APOE4 |  | [-133,245] | 56 |
| **Males, age [65-80], HV (mm^3^) right** |  |  |  |
| Tukey | 9.92e-01 |  |  |
| Infs_noAPOE4 |  | [4601,4873] | 4727 |
| noInfs_noAPOE4 |  | [4684,4731] | 4707 |
| Infs_noAPOE4 - noInfs_noAPOE4 |  | [-164,205] | 20 |
| **Males, age [60-75], HV (mm3) right** |  |  |  |
| Tukey | 4.27e-01 |  |  |
| noInfs_APOE4 |  | [4633,4710] | 4671 |
| noInfs_noAPOE4 |  | [4684,4731] | 4707 |
| noInfs_APOE4 - noInfs_noAPOE4 |  | [-97,25] | -36 |
|  |  |  |  |

**Note:** Infs and noInfs correspond to the groups of subjects with history of acute infection and without history of acute infection; APOE4 and noAPOE4 correspond to the groups of carriers of APOE e4 allele and non-carriers of APOE e4 allele. Comparisons are considered between the following groups: Infs_APOE4 (subjects who are both Infs and APOE4) and Infs_noAPOE4 (subjects who are in Infs and are not in APOE4), Infs_APOE4 and noInfs_APOE4 (subjects who are not in Infs and are in APOE4), Infs_APOE4 and noInfs_noAPOE4 (subjects who are not in Infs and are not in APOE4), Infs_noAPOE4 and noInfs_APOE4, Infs_noAPOE4 and noInfs_noAPOE4, noInfs_APOE4 and noInfs_noAPOE4. In this table, the sign minus between two groups denotes the difference between the means in two groups (effect size). For instance, Infs_APOE4 - Infs_noAPOE4 equals to the difference between the left/right HV mean value in the group Infs_APOE4 and group Infs_noAPOE4. Scientific notation ‘e’ means that the base number is multiplied by 10 raised to the given power.

**Supplementary Table 31.** Age distributions at the time of collecting acute infectious disease data between January 1, 2009 to October 15, 2014 for those attended assessment center during imaging visit between January 15, 2015 and October 31, 2019 at age 60-75 and 65-80. The groups that were compared shown in the Group column. The numbers are rounded to two decimal places.

| Group | min | max | mean | sd | number |
| --- | --- | --- | --- | --- | --- |
|  |  |  |  |  |  |
| **Female, age 60-75** |  |  |  |  |  |
| Infs APOE4 | 60.62 | 74.05 | 67.48 | 3.60 | 101 |
| Infs noAPOE4 | 60.02 | 74.99 | 67.75 | 4.25 | 252 |
| noInfs APOE4 | 60.01 | 74.98 | 67.21 | 4.04 | 1236 |
| noInfs noAPOE4 | 60.00 | 74.99 | 67.20 | 4.14 | 3165 |
| **Male, age 60-75** |  |  |  |  |  |
| Infs APOE4 | 60.02 | 74.67 | 67.02 | 4.45 | 61 |
| Infs noAPOE4 | 60.04 | 74.93 | 68.04 | 4.05 | 220 |
| noInfs APOE4 | 60.01 | 75.00 | 67.95 | 4.02 | 1112 |
| noInfs noAPOE4 | 60.00 | 75.00 | 68.05 | 4.07 | 3003 |
| **Female, age 65-80** |  |  |  |  |  |
| Infs APOE4 | 65.02 | 78.88 | 69.69 | 3.30 | 79 |
| Infs noAPOE4 | 65.05 | 79.75 | 71.29 | 3.52 | 203 |
| noInfs APOE4 | 65.02 | 79.93 | 70.29 | 3.48 | 924 |
| noInfs noAPOE4 | 65.01 | 79.99 | 70.66 | 3.62 | 2430 |
| **Male, age 65-80** |  |  |  |  |  |
| Infs APOE4 | 65.39 | 79.31 | 71.65 | 3.84 | 46 |
| Infs noAPOE4 | 65.05 | 79.42 | 71.17 | 3.67 | 190 |
| noInfs APOE4 | 65.00 | 80.00 | 71.20 | 3.66 | 989 |
| noInfs noAPOE4 | 65.00 | 79.99 | 71.23 | 3.67 | 2715 |
|  |  |  |  |  |  |

**Note:** Infs and noInfs correspond to the groups of subjects with history of infection and without history of infection; APOE4 and noAPOE4 correspond to the groups of carriers of APOE e4 allele and non-carriers of APOE e4 allele. Comparisons are considered between the following groups: Infs_APOE4 (subjects who are both Infs and APOE4) and Infs_noAPOE4 (subjects who are in Infs and are not in APOE4), Infs_APOE4 and noInfs_APOE4 (subjects who are not in Infs and are in APOE4), Infs_APOE4 and noInfs_noAPOE4 (subjects who are not in Infs and are not in APOE4), Infs_noAPOE4 and noInfs_APOE4, Infs_noAPOE4 and noInfs_noAPOE4, noInfs_APOE4 and noInfs_noAPOE4.

**Supplementary Table 32.** Welch Two Sample t-test for age distributions at the time of collecting acute infectious disease data between January 1, 2009 to October 15, 2014 for those attended assessment center during imaging visit between January 15, 2015 and October 31, 2019 at age 60-75 and 65-80. The groups that were compared shown in the Group column. The numbers are rounded to two decimal places.

| Group | P-value | 95% Confidence interval | Estimate | stderr |
| --- | --- | --- | --- | --- |
|  |  |  |  |  |
| **Female, age 60-75** |  |  |  |  |
| Infs APOE4 : Infs noAPOE4 | 5.34e-01 | [-1.16,0.60] | 67.48 : 67.75 | 0.45 |
| Infs APOE4 : noInfs APOE4 | 7.08e-01 | [-0.48,1.01] | 67.48 : 67.21 | 0.38 |
| Infs APOE4 : noInfs noAPOE4 | 7.74e-01 | [-0.44,1.01] | 67.48 : 67.20 | 0.37 |
| Infs noAPOE4 : noInfs APOE4 | 6.20e-02 | [-0.03,1.12] | 67.75 : 67.21 | 0.29 |
| Infs noAPOE4 : noInfs noAPOE4 | 4.38e-02 | [0.02,1.12] | 67.75 : 67.20 | 0.28 |
| noInfs APOE4 : noInfs noAPOE4 | 9.03e-01 | [-0.25,0.28] | 67.21 : 67.20 | 0.09 |
| **Male, age 60-75** |  |  |  |  |
| Infs APOE4 : Infs noAPOE4 | 1.11e-01 | [-2.27,0.24] | 67.02 : 68.04 | 0.63 |
| Infs APOE4 : noInfs APOE4 | 1.16e-01 | [-2.09,0.24] | 67.02 : 67.95 | 0.58 |
| Infs APOE4 : noInfs noAPOE4 | 7.86e-02 | [-2.18,0.13] | 67.02 : 68.05 | 0.57 |
| Infs noAPOE4 : noInfs APOE4 | 7.69e-01 | [-0.50,0.68] | 68.04 : 67.95 | 0.30 |
| Infs noAPOE4 : noInfs noAPOE4 | 9.67e-01 | [-0.57,0.28] | 68.04 : 68.05 | 0.28 |
| noInfs APOE4 : noInfs noAPOE4 | 4.81e-01 | [-0.38,0.19] | 67.95 : 68.05 | 0.14 |
| **Female, age 65-80** |  |  |  |  |
| Infs APOE4 : Infs noAPOE4 | 4.50e-04 | [-2.48,-0.72] | 69.69 : 71.29 | 0.45 |
| Infs APOE4 : noInfs APOE4 | 1.26e-01 | [-1.37,0.17] | 69.69 : 70.29 | 0.39 |
| Infs APOE4 : noInfs noAPOE4 | 1.20e-02 | [-1.73,-0.22] | 69.69 : 70.66 | 0.38 |
| Infs noAPOE4 : noInfs APOE4 | 2.84e-04 | [0.47,1.54] | 71.29 : 70.29 | 0.27 |
| Infs noAPOE4 : noInfs noAPOE4 | 1.55e-02 | [0.12,1.14] | 71.29 : 70.66 | 0.26 |
| noInfs APOE4 : noInfs noAPOE4 | 6.15e-03 | [-0.63,-0.11] | 70.29 : 70.66 | 0.14 |
| **Male, age 65-80** |  |  |  |  |
| Infs APOE4 : Infs noAPOE4 | 4.47e-01 | [-0.77,1.73] | 71.65 : 71.17 | 0.63 |
| Infs APOE4 : noInfs APOE4 | 4.40e-01 | [-0.71,1.61] | 71.65 : 71.20 | 0.58 |
| Infs APOE4 : noInfs noAPOE4 | 4.61e-01 | [-0.72,1.57] | 71.65 : 71.23 | 0.57 |
| Infs noAPOE4 : noInfs APOE4 | 9.22e-01 | [-0.60,0.54] | 71.17 : 71.20 | 0.29 |
| Infs noAPOE4 : noInfs noAPOE4 | 8.42e-01 | [-0.60,0.49] | 71.17 : 71.23 | 0.28 |
| noInfs APOE4 : noInfs noAPOE4 | 8.45e-01 | [-0.29,0.24] | 71.20 : 71.23 | 0.14 |
|  |  |  |  |  |

**Note:** Infs and noInfs correspond to the groups of subjects with history of infection and without history of infection; APOE4 and noAPOE4 correspond to the groups of carriers of APOE e4 allele and non-carriers of APOE e4 allele. Comparisons are considered between the following groups: Infs_APOE4 (subjects who are both Infs and APOE4) and Infs_noAPOE4 (subjects who are in Infs and are not in APOE4), Infs_APOE4 and noInfs_APOE4 (subjects who are not in Infs and are in APOE4), Infs_APOE4 and noInfs_noAPOE4 (subjects who are not in Infs and are not in APOE4), Infs_noAPOE4 and noInfs_APOE4, Infs_noAPOE4 and noInfs_noAPOE4, noInfs_APOE4 and noInfs_noAPOE4.

**Supplementary Table 33**. Regression models, female, age 60-75. Response variable HV=HV (mm^3^) left, independent variables: *Age* – age at the time attending assessment center during imaging visit, *infs*=1 (with history of infections), *infs*=0 (without history of infections), *apoe4*=1 (APOE e4 carrier), *apoe4*=0 (APOE e4 non-carrier). Per the regression set corresponding to age groups 65-80 and left HV values, all 64 models (with linear terms and their pairwise interactions) in each regression set were analyzed and presented in ascending order by AIC value. Signf=1 means that all regression coefficients were significant (P-value<0.05) in a specific model, Signf=0 means the opposite. For regression model a short notation used. For instance, HV ~ 1 + *Age* + *infs* + *apoe4* + *infs***Age* + *apoe4***infs* denotes a standard regression equation HV = Intercept + b_1_**Age* + b_2_**infs* + b_3_**apoe4* + b_12_**Age***infs* + b_31_**apoe4***infs* where Intercept is a constant called the bias term (or intercept term), b_1_, b_2_, b_3_, b_12_, b_31_ are the regression coefficients corresponding to the *Age*, *infs*, *apoe4*, *Age***infs*, *apoe4***infs* terms in the regression model.

| Regression Model | AIC | Signf |
| --- | --- | --- |
|  |  |  |
| HV ~ 1 + *Age* + *apoe4* + *apoe4***Age* | 73554.04 | 0 |
| HV ~ 1 + *Age* + *apoe4***Age* | 73554.24 | 0 |
| HV ~ 1 + *Age* + *apoe4* + *infs***Age* + *apoe4***Age* | 73554.29 | 0 |
| HV ~ 1 + *Age* + *infs* + *apoe4* + *apoe4***Age* | 73554.30 | 0 |
| HV ~ 1 + *Age* + *infs* + *apoe4***Age* | 73554.53 | 0 |
| HV ~ 1 + *Age* + *infs***Age* + *apoe4***Age* | 73554.53 | 0 |
| HV ~ 1 + *Age* + *apoe4* | 73554.56 | 0 |
| HV ~ 1 + *Age* + *infs* + *apoe4* | 73554.86 | 0 |
| HV ~ 1 + *Age* + *apoe4* + *infs***Age* | 73554.86 | 0 |
| HV ~ 1 + *Age* | 73555.50 | 1 |
| HV ~ 1 + *Age* + *infs* + *apoe4* + *apoe4***Age* + *apoe4***infs* | 73555.66 | 0 |
| HV ~ 1 + *Age* + *apoe4* + *infs***Age* + *apoe4***Age* + *apoe4***infs* | 73555.66 | 0 |
| HV ~ 1 + *Age* + *infs* | 73555.80 | 0 |
| HV ~ 1 + *Age* + *infs***Age* | 73555.80 | 0 |
| HV ~ 1 + *Age* + *infs* + *apoe4***Age* + *apoe4***infs* | 73555.90 | 0 |
| HV ~ 1 + *Age* + *infs***Age* + *apoe4***Age* + *apoe4***infs* | 73555.92 | 0 |
| HV ~ 1 + *Age* + *apoe4* + *apoe4***Age* + *apoe4***infs* | 73556.03 | 0 |
| HV ~ 1 + *Age* + *apoe4***Age* + *apoe4***infs* | 73556.23 | 0 |
| HV ~ 1 + *Age* + *infs* + *apoe4* + *apoe4***infs* | 73556.28 | 0 |
| HV ~ 1 + *Age* + *apoe4* + *infs***Age* + *apoe4***infs* | 73556.29 | 0 |
| HV ~ 1 + *Age* + *infs* + *apoe4* + *infs***Age* + *apoe4***Age* | 73556.29 | 0 |
| HV ~ 1 + *Age* + *infs* + *infs***Age* + *apoe4***Age* | 73556.53 | 0 |
| HV ~ 1 + *Age* + *apoe4* + *apoe4***infs* | 73556.56 | 0 |
| HV ~ 1 + *Age* + *infs* + *apoe4* + *infs***Age* | 73556.86 | 0 |
| HV ~ 1 + *Age* + *apoe4***infs* | 73557.30 | 0 |
| HV ~ 1 + *Age* + *infs* + *apoe4* + *infs***Age* + *apoe4***Age* + *apoe4***infs* | 73557.65 | 0 |
| HV ~ 1 + *Age* + *infs* + *apoe4***infs* | 73557.73 | 0 |
| HV ~ 1 + *Age* + *infs***Age* + *apoe4***infs* | 73557.73 | 0 |
| HV ~ 1 + *Age* + *infs* + *infs***Age* | 73557.80 | 0 |
| HV ~ 1 + *Age* + *infs* + *infs***Age* + *apoe4***Age* + *apoe4***infs* | 73557.90 | 0 |
| HV ~ 1 + *Age* + *infs* + *apoe4* + *infs***Age* + *apoe4***infs* | 73558.28 | 0 |
| HV ~ 1 + *Age* + *infs* + *infs***Age* + *apoe4***infs* | 73559.73 | 0 |
| HV ~ 1 + *infs* + *apoe4* + *infs***Age* + *apoe4***Age* | 73663.02 | 1 |
| HV ~ 1 + *infs* + *apoe4* + *infs***Age* + *apoe4***Age* + *apoe4***infs* | 73664.18 | 0 |
| HV ~ 1 + *apoe4* + *infs***Age* + *apoe4***Age* | 73667.82 | 0 |
| HV ~ 1 + *infs* + *apoe4* + *apoe4***Age* | 73668.39 | 0 |
| HV ~ 1 + *apoe4* + *infs***Age* + *apoe4***Age* + *apoe4***infs* | 73668.62 | 0 |
| HV ~ 1 + *apoe4* + *apoe4***Age* | 73669.08 | 1 |
| HV ~ 1 + *infs* + *apoe4* + *apoe4***Age* + *apoe4***infs* | 73669.39 | 0 |
| HV ~ 1 + *apoe4* + *apoe4***Age* + *apoe4***infs* | 73671.08 | 0 |
| HV ~ 1 + *infs* + *infs***Age* + *apoe4***Age* | 73721.75 | 1 |
| HV ~ 1 + *infs* + *infs***Age* + *apoe4***Age* + *apoe4***infs* | 73722.91 | 0 |
| HV ~ 1 + *infs* + *apoe4* + *infs***Age* | 73723.93 | 0 |
| HV ~ 1 + *infs* + *infs***Age* | 73724.88 | 1 |
| HV ~ 1 + *infs* + *apoe4* + *infs***Age* + *apoe4***infs* | 73725.35 | 0 |
| HV ~ 1 + *infs* + *infs***Age* + *apoe4***infs* | 73726.82 | 0 |
| HV ~ 1 + *infs***Age* + *apoe4***Age* | 73732.04 | 0 |
| HV ~ 1 + *infs***Age* + *apoe4***Age* + *apoe4***infs* | 73732.68 | 0 |
| HV ~ 1 + *infs* + *apoe4***Age* | 73732.84 | 0 |
| HV ~ 1 + *apoe4***Age* | 73733.74 | 1 |
| HV ~ 1 + *infs* + *apoe4***Age* + *apoe4***infs* | 73733.77 | 0 |
| HV ~ 1 + *apoe4* + *infs***Age* | 73734.30 | 0 |
| HV ~ 1 + *infs* + *apoe4* | 73735.11 | 0 |
| HV ~ 1 + *infs***Age* | 73735.15 | 0 |
| HV ~ 1 + *apoe4* + *infs***Age* + *apoe4***infs* | 73735.30 | 0 |
| HV ~ 1 + *apoe4***Age* + *apoe4***infs* | 73735.74 | 0 |
| HV ~ 1 + *infs* | 73735.95 | 0 |
| HV ~ 1 + *apoe4* | 73736.02 | 0 |
| HV ~ 1 + *infs* + *apoe4* + *apoe4***infs* | 73736.36 | 0 |
| HV ~ 1 | 73736.86 | 1 |
| HV ~ 1 + *infs***Age* + *apoe4***infs* | 73736.89 | 0 |
| HV ~ 1 + *infs* + *apoe4***infs* | 73737.81 | 0 |
| HV ~ 1 + *apoe4* + *apoe4***infs* | 73737.99 | 0 |
| HV ~ 1 + *apoe4***infs* | 73738.55 | 0 |
|  |  |  |

**Supplementary Table 34.** In this table, regression coefficients for the best significant model (number 10 in the list) among all models that are presented in ascending order by AIC value in the Supplementary Table 33. Scientific notation ‘e’ means that the base number is multiplied by 10 raised to the given power.

| Model/Term | Estimate | Std. Error | P-value |
| --- | --- | --- | --- |
|  |  |  |  |
| **Best model for HV** **(mm^3^) left, female 60-75** |  |  |  |
| Intercept | 6847.92 (mm^3^) | 136.99 | < 1.00e-50 |
| *Age* | -27.80 (mm^3^/year) | 2.03 | 9.40e-42 |
|  |  |  |  |

**Supplementary Table 35**. Regression models, female, age 65-80. Response variable HV=HV (mm^3^) left, independent variables: *Age* – age at the time attending assessment center during imaging visit, *infs*=1 (with history of infections), *infs*=0 (without history of infections), *apoe4*=1 (APOE e4 carrier), *apoe4*=0 (APOE e4 non-carrier). Per the regression set corresponding to age groups 65-80 and left HV values, all 64 models (with linear terms and their pairwise interactions) in each regression set were analyzed and presented in ascending order by AIC value. Signf=1 means that all regression coefficients were significant (P-value<0.05) in a specific model, Signf=0 means the opposite. For regression model a short notation used. For instance, HV ~ 1 + *Age* + *infs* + *apoe4* + *infs***Age* + *apoe4***infs* denotes a standard regression equation HV = Intercept + b_1_**Age* + b_2_**infs* + b_3_**apoe4* + b_12_**Age***infs* + b_31_**apoe4***infs* where Intercept is a constant called the bias term (or intercept term), b_1_, b_2_, b_3_, b_12_, b_31_ are the regression coefficients corresponding to the *Age*, *infs*, *apoe4*, *Age***infs*, *apoe4***infs* terms in the regression model.

| Regression Model | AIC | Signf |
| --- | --- | --- |
|  |  |  |
| HV ~ 1 + *Age* + *infs***Age* + *apoe4***Age* | 56201.10 | 0 |
| HV ~ 1 + *Age* + *infs* + *apoe4***Age* | 56201.13 | 0 |
| HV ~ 1 + *Age* + *apoe4* + *infs***Age* | 56201.38 | 0 |
| HV ~ 1 + *Age* + *infs* + *apoe4* | 56201.41 | 0 |
| HV ~ 1 + *Age* + *infs* + *apoe4***Age* + *apoe4***infs* | 56201.49 | 0 |
| HV ~ 1 + *Age* + *infs***Age* + *apoe4***Age* + *apoe4***infs* | 56201.51 | 0 |
| HV ~ 1 + *Age* + *apoe4***Age* | 56201.71 | 1 |
| HV ~ 1 + *Age* + *infs* + *apoe4* + *apoe4***infs* | 56201.80 | 0 |
| HV ~ 1 + *Age* + *apoe4* + *infs***Age* + *apoe4***infs* | 56201.82 | 0 |
| HV ~ 1 + *Age* + *apoe4* | 56201.97 | 1 |
| HV ~ 1 + *Age* + *apoe4* + *infs***Age* + *apoe4***Age* | 56201.98 | 0 |
| HV ~ 1 + *Age* + *infs* + *apoe4* + *apoe4***Age* | 56202.01 | 0 |
| HV ~ 1 + *Age* + *infs* + *apoe4* + *apoe4***Age* + *apoe4***infs* | 56202.45 | 0 |
| HV ~ 1 + *Age* + *apoe4* + *infs***Age* + *apoe4***Age* + *apoe4***infs* | 56202.47 | 0 |
| HV ~ 1 + *Age* + *apoe4* + *apoe4***Age* | 56202.72 | 0 |
| HV ~ 1 + *Age* + *infs* + *infs***Age* + *apoe4***Age* | 56203.08 | 0 |
| HV ~ 1 + *Age* + *infs* + *apoe4* + *infs***Age* | 56203.36 | 0 |
| HV ~ 1 + *Age* + *infs* + *infs***Age* + *apoe4***Age* + *apoe4***infs* | 56203.49 | 0 |
| HV ~ 1 + *Age* + *apoe4***Age* + *apoe4***infs* | 56203.65 | 0 |
| HV ~ 1 + *Age* + *infs* + *apoe4* + *infs***Age* + *apoe4***infs* | 56203.80 | 0 |
| HV ~ 1 + *Age* + *apoe4* + *apoe4***infs* | 56203.92 | 0 |
| HV ~ 1 + *Age* + *infs* + *apoe4* + *infs***Age* + *apoe4***Age* | 56203.95 | 0 |
| HV ~ 1 + *Age* + *infs* + *apoe4* + *infs***Age* + *apoe4***Age* + *apoe4***infs* | 56204.45 | 0 |
| HV ~ 1 + *Age* + *apoe4* + *apoe4***Age* + *apoe4***infs* | 56204.68 | 0 |
| HV ~ 1 + *Age* + *infs***Age* | 56205.47 | 0 |
| HV ~ 1 + *Age* + *infs* | 56205.49 | 0 |
| HV ~ 1 + *Age* | 56206.05 | 1 |
| HV ~ 1 + *Age* + *infs* + *apoe4***infs* | 56207.20 | 0 |
| HV ~ 1 + *Age* + *infs***Age* + *apoe4***infs* | 56207.20 | 0 |
| HV ~ 1 + *Age* + *infs* + *infs***Age* | 56207.47 | 0 |
| HV ~ 1 + *Age* + *apoe4***infs* | 56207.93 | 0 |
| HV ~ 1 + *Age* + *infs* + *infs***Age* + *apoe4***infs* | 56209.20 | 0 |
| HV ~ 1 + *infs* + *apoe4* + *infs***Age* + *apoe4***Age* | 56305.94 | 1 |
| HV ~ 1 + *infs* + *apoe4* + *infs***Age* + *apoe4***Age* + *apoe4***infs* | 56306.94 | 0 |
| HV ~ 1 + *apoe4* + *infs***Age* + *apoe4***Age* + *apoe4***infs* | 56310.34 | 0 |
| HV ~ 1 + *apoe4* + *infs***Age* + *apoe4***Age* | 56310.70 | 1 |
| HV ~ 1 + *infs* + *apoe4* + *apoe4***Age* + *apoe4***infs* | 56311.06 | 0 |
| HV ~ 1 + *infs* + *apoe4* + *apoe4***Age* | 56311.27 | 1 |
| HV ~ 1 + *apoe4* + *apoe4***Age* | 56313.44 | 1 |
| HV ~ 1 + *apoe4* + *apoe4***Age* + *apoe4***infs* | 56315.40 | 0 |
| HV ~ 1 + *infs* + *infs***Age* + *apoe4***Age* | 56352.52 | 1 |
| HV ~ 1 + *infs* + *infs***Age* + *apoe4***Age* + *apoe4***infs* | 56353.13 | 0 |
| HV ~ 1 + *infs* + *apoe4* + *infs***Age* | 56354.19 | 0 |
| HV ~ 1 + *infs* + *apoe4* + *infs***Age* + *apoe4***infs* | 56355.05 | 0 |
| HV ~ 1 + *infs* + *infs***Age* | 56355.73 | 1 |
| HV ~ 1 + *infs* + *infs***Age* + *apoe4***infs* | 56357.46 | 0 |
| HV ~ 1 + *infs***Age* + *apoe4***Age* + *apoe4***infs* | 56361.61 | 0 |
| HV ~ 1 + *infs* + *apoe4***Age* + *apoe4***infs* | 56362.65 | 0 |
| HV ~ 1 + *infs***Age* + *apoe4***Age* | 56363.35 | 1 |
| HV ~ 1 + *apoe4* + *infs***Age* + *apoe4***infs* | 56363.68 | 0 |
| HV ~ 1 + *infs* + *apoe4***Age* | 56364.09 | 0 |
| HV ~ 1 + *infs* + *apoe4* + *apoe4***infs* | 56364.70 | 0 |
| HV ~ 1 + *apoe4* + *infs***Age* | 56364.98 | 0 |
| HV ~ 1 + *apoe4***Age* | 56365.58 | 1 |
| HV ~ 1 + *infs* + *apoe4* | 56365.72 | 0 |
| HV ~ 1 + *infs***Age* | 56365.83 | 1 |
| HV ~ 1 + *infs***Age* + *apoe4***infs* | 56366.19 | 0 |
| HV ~ 1 + *infs* | 56366.54 | 0 |
| HV ~ 1 + *infs* + *apoe4***infs* | 56367.10 | 0 |
| HV ~ 1 + *apoe4* | 56367.20 | 0 |
| HV ~ 1 + *apoe4***Age* + *apoe4***infs* | 56367.22 | 0 |
| HV ~ 1 | 56368.01 | 1 |
| HV ~ 1 + *apoe4* + *apoe4***infs* | 56368.95 | 0 |
| HV ~ 1 + *apoe4***infs* | 56370.00 | 0 |
|  |  |  |

**Supplementary Table 36.** In this table, regression coefficients for the best significant model (number 7 in the list) among all models that are presented in ascending order by AIC value in the Supplementary Table35. Scientific notation ‘e’ means that the base number is multiplied by 10 raised to the given power.

| Model/Term | Estimate | Std. Error | P-value |
| --- | --- | --- | --- |
|  |  |  |  |
| **Best model for HV** **(mm^3^) left, female 65-80** |  |  |  |
| Intercept | 7349.20 (mm^3^) | 188.09 | < 1.00e-50 |
| *Age* | -34.62 (mm^3^/year) | 2.66 | 6.40e-38 |
| *Age*apoe4* | -0.76 (mm^3^/year) | 0.30 | 1.18e-02 |
|  |  |  |  |

**Supplementary Table 37.** Regression models, male, age 60-75. Response variable HV=HV (mm^3^) left, independent variables: *Age* – age at the time attending assessment center during imaging visit, *infs*=1 (with history of infections), *infs*=0 (without history of infections), *apoe4*=1 (APOE e4 carrier), *apoe4*=0 (APOE e4 non-carrier). Per the regression set corresponding to age groups 65-80 and left HV values, all 64 models (with linear terms and their pairwise interactions) in each regression set were analyzed and presented in ascending order by AIC value. Signf=1 means that all regression coefficients were significant (P-value<0.05) in a specific model, Signf=0 means the opposite. For regression model a short notation used. For instance, HV ~ 1 + *Age* + *infs* + *apoe4* + *infs***Age* + *apoe4***infs* denotes a standard regression equation HV = Intercept + b_1_**Age* + b_2_**infs* + b_3_**apoe4* + b_12_**Age***infs* + b_31_**apoe4***infs* where Intercept is a constant called the bias term (or intercept term), b_1_, b_2_, b_3_, b_12_, b_31_ are the regression coefficients corresponding to the *Age*, *infs*, *apoe4*, *Age***infs*, *apoe4***infs* terms in the regression model.

| Regression Model | AIC | Signf |
| --- | --- | --- |
|  |  |  |
| HV ~ 1 + *Age* + *apoe4* | 68297.01 | 0 |
| HV ~ 1 + *Age* + *apoe4***Age* | 68297.13 | 0 |
| HV ~ 1 + *Age* | 68297.68 | 1 |
| HV ~ 1 + *Age* + *infs* + *apoe4* | 68297.96 | 0 |
| HV ~ 1 + *Age* + *apoe4* + *infs***Age* | 68297.98 | 0 |
| HV ~ 1 + *Age* + *infs* + *apoe4***Age* | 68298.08 | 0 |
| HV ~ 1 + *Age* + *infs***Age* + *apoe4***Age* | 68298.09 | 0 |
| HV ~ 1 + *Age* + *apoe4* + *apoe4***infs* | 68298.37 | 0 |
| HV ~ 1 + *Age* + *apoe4***infs* | 68298.47 | 0 |
| HV ~ 1 + *Age* + *apoe4***Age* + *apoe4***infs* | 68298.47 | 0 |
| HV ~ 1 + *Age* + *apoe4* + *apoe4***Age* | 68298.68 | 0 |
| HV ~ 1 + *Age* + *infs* | 68298.74 | 0 |
| HV ~ 1 + *Age* + *infs***Age* | 68298.76 | 0 |
| HV ~ 1 + *Age* + *infs* + *apoe4* + *apoe4***Age* | 68299.66 | 0 |
| HV ~ 1 + *Age* + *apoe4* + *infs***Age* + *apoe4***Age* | 68299.68 | 0 |
| HV ~ 1 + *Age* + *infs* + *apoe4* + *apoe4***infs* | 68299.83 | 0 |
| HV ~ 1 + *Age* + *apoe4* + *infs***Age* + *apoe4***infs* | 68299.84 | 0 |
| HV ~ 1 + *Age* + *infs* + *apoe4***Age* + *apoe4***infs* | 68299.94 | 0 |
| HV ~ 1 + *Age* + *infs***Age* + *apoe4***Age* + *apoe4***infs* | 68299.94 | 0 |
| HV ~ 1 + *Age* + *infs* + *apoe4* + *infs***Age* | 68299.96 | 0 |
| HV ~ 1 + *Age* + *infs* + *infs***Age* + *apoe4***Age* | 68300.08 | 0 |
| HV ~ 1 + *Age* + *apoe4* + *apoe4***Age* + *apoe4***infs* | 68300.09 | 0 |
| HV ~ 1 + *Age* + *infs* + *apoe4***infs* | 68300.19 | 0 |
| HV ~ 1 + *Age* + *infs***Age* + *apoe4***infs* | 68300.19 | 0 |
| HV ~ 1 + *Age* + *infs* + *infs***Age* | 68300.73 | 0 |
| HV ~ 1 + *Age* + *infs* + *apoe4* + *apoe4***Age* + *apoe4***infs* | 68301.55 | 0 |
| HV ~ 1 + *Age* + *apoe4* + *infs***Age* + *apoe4***Age* + *apoe4***infs* | 68301.55 | 0 |
| HV ~ 1 + *Age* + *infs* + *apoe4* + *infs***Age* + *apoe4***Age* | 68301.66 | 0 |
| HV ~ 1 + *Age* + *infs* + *apoe4* + *infs***Age* + *apoe4***infs* | 68301.83 | 0 |
| HV ~ 1 + *Age* + *infs* + *infs***Age* + *apoe4***Age* + *apoe4***infs* | 68301.94 | 0 |
| HV ~ 1 + *Age* + *infs* + *infs***Age* + *apoe4***infs* | 68302.19 | 0 |
| HV ~ 1 + *Age* + *infs* + *apoe4* + *infs***Age* + *apoe4***Age* + *apoe4***infs* | 68303.55 | 0 |
| HV ~ 1 + *infs* + *apoe4* + *infs***Age* + *apoe4***Age* | 68451.18 | 1 |
| HV ~ 1 + *infs* + *apoe4* + *infs***Age* + *apoe4***Age* + *apoe4***infs* | 68452.81 | 0 |
| HV ~ 1 + *apoe4* + *apoe4***Age* | 68455.84 | 1 |
| HV ~ 1 + *apoe4* + *infs***Age* + *apoe4***Age* | 68456.51 | 0 |
| HV ~ 1 + *infs* + *apoe4* + *apoe4***Age* | 68456.87 | 0 |
| HV ~ 1 + *apoe4* + *apoe4***Age* + *apoe4***infs* | 68457.26 | 0 |
| HV ~ 1 + *apoe4* + *infs***Age* + *apoe4***Age* + *apoe4***infs* | 68458.44 | 0 |
| HV ~ 1 + *infs* + *apoe4* + *apoe4***Age* + *apoe4***infs* | 68458.76 | 0 |
| HV ~ 1 + *infs* + *infs***Age* + *apoe4***Age* | 68489.62 | 0 |
| HV ~ 1 + *infs* + *apoe4* + *infs***Age* | 68491.12 | 0 |
| HV ~ 1 + *infs* + *infs***Age* | 68491.31 | 1 |
| HV ~ 1 + *infs* + *infs***Age* + *apoe4***Age* + *apoe4***infs* | 68491.54 | 0 |
| HV ~ 1 + *infs* + *infs***Age* + *apoe4***infs* | 68492.79 | 0 |
| HV ~ 1 + *infs* + *apoe4* + *infs***Age* + *apoe4***infs* | 68492.97 | 0 |
| HV ~ 1 + *apoe4***Age* | 68499.30 | 0 |
| HV ~ 1 + *infs***Age* + *apoe4***Age* | 68500.20 | 0 |
| HV ~ 1 + *infs* + *apoe4***Age* | 68500.62 | 0 |
| HV ~ 1 | 68500.67 | 1 |
| HV ~ 1 + *apoe4* | 68500.81 | 0 |
| HV ~ 1 + *apoe4***Age* + *apoe4***infs* | 68501.20 | 0 |
| HV ~ 1 + *infs***Age* | 68501.69 | 0 |
| HV ~ 1 + *apoe4* + *infs***Age* | 68501.74 | 0 |
| HV ~ 1 + *infs* | 68502.09 | 0 |
| HV ~ 1 + *infs***Age* + *apoe4***Age* + *apoe4***infs* | 68502.16 | 0 |
| HV ~ 1 + *infs* + *apoe4* | 68502.16 | 0 |
| HV ~ 1 + *apoe4***infs* | 68502.25 | 0 |
| HV ~ 1 + *infs* + *apoe4***Age* + *apoe4***infs* | 68502.61 | 0 |
| HV ~ 1 + *apoe4* + *apoe4***infs* | 68502.65 | 0 |
| HV ~ 1 + *infs***Age* + *apoe4***infs* | 68503.64 | 0 |
| HV ~ 1 + *apoe4* + *infs***Age* + *apoe4***infs* | 68503.73 | 0 |
| HV ~ 1 + *infs* + *apoe4***infs* | 68503.97 | 0 |
| HV ~ 1 + *infs* + *apoe4* + *apoe4***infs* | 68504.16 | 0 |
|  |  |  |

**Supplementary Table 38.** In this table, regression coefficients for the best significant model (number 3 in the list) among all models that are presented in ascending order by AIC value in the Supplementary Table 37. Scientific notation ‘e’ means that the base number is multiplied by 10 raised to the given power.

| Model/Term | Estimate | Std. Error | P-value |
| --- | --- | --- | --- |
|  |  |  |  |
| **Best model for HV** **(mm^3^) left, male 60-75** |  |  |  |
| Intercept | 6839.76 (mm^3^) | 150.62 | < 1.00e-50 |
| *Age* | -32.02 (mm^3^/year) | 2.21 | 1.81e-46 |
|  |  |  |  |

**Supplementary Table 39** Regression models, male, age 65-80. Response variable HV=HV (mm^3^) left, independent variables: *Age* – age at the time attending assessment center during imaging visit, *infs*=1 (with history of infections), *infs*=0 (without history of infections), *apoe4*=1 (APOE e4 carrier), *apoe4*=0 (APOE e4 non-carrier). Per the regression set corresponding to age groups 65-80 and left HV values, all 64 models (with linear terms and their pairwise interactions) in each regression set were analyzed and presented in ascending order by AIC value. Signf=1 means that all regression coefficients were significant (P-value<0.05) in a specific model, Signf=0 means the opposite. For regression model a short notation used. For instance, HV ~ 1 + *Age* + *infs* + *apoe4* + *infs***Age* + *apoe4***infs* denotes a standard regression equation HV = Intercept + b_1_**Age* + b_2_**infs* + b_3_**apoe4* + b_12_**Age***infs* + b_31_**apoe4***infs* where Intercept is a constant called the bias term (or intercept term), b_1_, b_2_, b_3_, b_12_, b_31_ are the regression coefficients corresponding to the *Age*, *infs*, *apoe4*, *Age***infs*, *apoe4***infs* terms in the regression model.

| Regression Model | AIC | Signf |
| --- | --- | --- |
|  |  |  |
| HV ~ 1 + *Age* + *apoe4***Age* | 61143.48 | 0 |
| HV ~ 1 + *Age* + *apoe4* | 61143.59 | 0 |
| HV ~ 1 + *Age* | 61143.69 | 1 |
| HV ~ 1 + *Age* + *apoe4* + *apoe4***Age* | 61145.03 | 0 |
| HV ~ 1 + *Age* + *infs* + *apoe4***Age* | 61145.35 | 0 |
| HV ~ 1 + *Age* + *infs***Age* + *apoe4***Age* | 61145.36 | 0 |
| HV ~ 1 + *Age* + *infs* + *apoe4* | 61145.46 | 0 |
| HV ~ 1 + *Age* + *apoe4* + *infs***Age* | 61145.47 | 0 |
| HV ~ 1 + *Age* + *apoe4***Age* + *apoe4***infs* | 61145.47 | 0 |
| HV ~ 1 + *Age* + *apoe4* + *apoe4***infs* | 61145.58 | 0 |
| HV ~ 1 + *Age* + *infs* | 61145.61 | 0 |
| HV ~ 1 + *Age* + *infs***Age* | 61145.61 | 0 |
| HV ~ 1 + *Age* + *apoe4***infs* | 61145.65 | 0 |
| HV ~ 1 + *Age* + *infs* + *apoe4* + *apoe4***Age* | 61146.91 | 0 |
| HV ~ 1 + *Age* + *apoe4* + *infs***Age* + *apoe4***Age* | 61146.92 | 0 |
| HV ~ 1 + *Age* + *apoe4* + *apoe4***Age* + *apoe4***infs* | 61147.03 | 0 |
| HV ~ 1 + *Age* + *infs* + *apoe4***Age* + *apoe4***infs* | 61147.29 | 0 |
| HV ~ 1 + *Age* + *infs***Age* + *apoe4***Age* + *apoe4***infs* | 61147.30 | 0 |
| HV ~ 1 + *Age* + *infs* + *infs***Age* + *apoe4***Age* | 61147.31 | 0 |
| HV ~ 1 + *Age* + *infs* + *apoe4* + *apoe4***infs* | 61147.40 | 0 |
| HV ~ 1 + *Age* + *apoe4* + *infs***Age* + *apoe4***infs* | 61147.41 | 0 |
| HV ~ 1 + *Age* + *infs* + *apoe4* + *infs***Age* | 61147.41 | 0 |
| HV ~ 1 + *Age* + *infs* + *infs***Age* | 61147.57 | 0 |
| HV ~ 1 + *Age* + *infs* + *apoe4***infs* | 61147.60 | 0 |
| HV ~ 1 + *Age* + *infs***Age* + *apoe4***infs* | 61147.60 | 0 |
| HV ~ 1 + *Age* + *infs* + *apoe4* + *apoe4***Age* + *apoe4***infs* | 61148.84 | 0 |
| HV ~ 1 + *Age* + *apoe4* + *infs***Age* + *apoe4***Age* + *apoe4***infs* | 61148.85 | 0 |
| HV ~ 1 + *Age* + *infs* + *apoe4* + *infs***Age* + *apoe4***Age* | 61148.87 | 0 |
| HV ~ 1 + *Age* + *infs* + *infs***Age* + *apoe4***Age* + *apoe4***infs* | 61149.25 | 0 |
| HV ~ 1 + *Age* + *infs* + *apoe4* + *infs***Age* + *apoe4***infs* | 61149.36 | 0 |
| HV ~ 1 + *Age* + *infs* + *infs***Age* + *apoe4***infs* | 61149.56 | 0 |
| HV ~ 1 + *Age* + *infs* + *apoe4* + *infs***Age* + *apoe4***Age* + *apoe4***infs* | 61150.81 | 0 |
| HV ~ 1 + *infs* + *apoe4* + *infs***Age* + *apoe4***Age* | 61295.69 | 1 |
| HV ~ 1 + *infs* + *apoe4* + *infs***Age* + *apoe4***Age* + *apoe4***infs* | 61297.56 | 0 |
| HV ~ 1 + *apoe4* + *apoe4***Age* | 61298.82 | 1 |
| HV ~ 1 + *apoe4* + *infs***Age* + *apoe4***Age* | 61300.62 | 0 |
| HV ~ 1 + *infs* + *apoe4* + *apoe4***Age* | 61300.72 | 0 |
| HV ~ 1 + *apoe4* + *apoe4***Age* + *apoe4***infs* | 61300.81 | 0 |
| HV ~ 1 + *apoe4* + *infs***Age* + *apoe4***Age* + *apoe4***infs* | 61302.53 | 0 |
| HV ~ 1 + *infs* + *apoe4* + *apoe4***Age* + *apoe4***infs* | 61302.67 | 0 |
| HV ~ 1 + *infs* + *infs***Age* + *apoe4***Age* | 61354.75 | 0 |
| HV ~ 1 + *infs* + *infs***Age* | 61356.18 | 1 |
| HV ~ 1 + *infs* + *apoe4* + *infs***Age* | 61356.26 | 0 |
| HV ~ 1 + *infs* + *infs***Age* + *apoe4***Age* + *apoe4***infs* | 61356.65 | 0 |
| HV ~ 1 + *infs* + *infs***Age* + *apoe4***infs* | 61358.17 | 0 |
| HV ~ 1 + *infs* + *apoe4* + *infs***Age* + *apoe4***infs* | 61358.22 | 0 |
| HV ~ 1 + *apoe4***Age* | 61362.63 | 0 |
| HV ~ 1 | 61364.22 | 1 |
| HV ~ 1 + *apoe4* | 61364.24 | 0 |
| HV ~ 1 + *infs***Age* + *apoe4***Age* | 61364.27 | 0 |
| HV ~ 1 + *infs* + *apoe4***Age* | 61364.46 | 0 |
| HV ~ 1 + *apoe4***Age* + *apoe4***infs* | 61364.63 | 0 |
| HV ~ 1 + *apoe4* + *infs***Age* | 61365.90 | 0 |
| HV ~ 1 + *infs***Age* | 61365.94 | 0 |
| HV ~ 1 + *apoe4***infs* | 61366.06 | 0 |
| HV ~ 1 + *infs* + *apoe4* | 61366.08 | 0 |
| HV ~ 1 + *infs* | 61366.10 | 0 |
| HV ~ 1 + *infs***Age* + *apoe4***Age* + *apoe4***infs* | 61366.21 | 0 |
| HV ~ 1 + *apoe4* + *apoe4***infs* | 61366.22 | 0 |
| HV ~ 1 + *infs* + *apoe4***Age* + *apoe4***infs* | 61366.43 | 0 |
| HV ~ 1 + *apoe4* + *infs***Age* + *apoe4***infs* | 61367.89 | 0 |
| HV ~ 1 + *infs***Age* + *apoe4***infs* | 61367.91 | 0 |
| HV ~ 1 + *infs* + *apoe4***infs* | 61368.03 | 0 |
| HV ~ 1 + *infs* + *apoe4* + *apoe4***infs* | 61368.08 | 0 |
|  |  |  |

**Supplementary Table 40.** In this table, regression coefficients for the best significant model (number 3 in the list) among all models that are presented in ascending order by AIC value in the Supplementary Table 39. Scientific notation ‘e’ means that the base number is multiplied by 10 raised to the given power.

| Model/Term | Estimate | Std. Error | P-value |
| --- | --- | --- | --- |
|  |  |  |  |
| **Best model for HV** **(mm^3^) left, female 65-80** |  |  |  |
| Intercept | 7314.25 (mm^3^) | 183.13 | < 1.00e-50 |
| *Age* | -38.84 (mm^3^/year) | 2.57 | 2.73e-50 |
|  |  |  |  |

**References**

Environmental Exposures Metadata. Resource 2010. UK Biobank (2023). Available online at: https://www.ukbiobank.ac.uk [Accessed October 16, 2023].

Government services and information. What qualification levels mean (2023). Available online at: https://www.gov.uk/what-different-qualification-levels-mean/list-of-qualification-levels/[Accessed September 12, 2023].

Smith, M. S., Alfaro-Almagro, F., and Miller, K. L. (2022). UK Biobank Brain Imaging Documentation, Version 1.9, December 2022. Wellcome Centre for Integrative Neuroimaging (WIN-FMRIB), Oxford University on behalf of UK Biobank. Available online at: https://biobank.ndph.ox.ac.uk.

UK Biobank (2023). Available online at: https://www.ukbiobank.ac.uk/[Accessed September 12, 2023].

Yashin, A. I., Fang, F., Kovtun, M., Wu, D., Duan, M., Arbeev, K., Akushevich, I., Kulminski, A., Culminskaya, I., Zhbannikov, I., Yashkin, A., Stallard, E., & Ukraintseva, S. (2018). Hidden heterogeneity in Alzheimer's disease: Insights from genetic association studies and other analyses. *Experimental gerontology*, *107*, 148–160. https://doi.org/10.1016/j.exger.2017.10.020
